# Mechanistic insight into the active centers of single/dual-atom Ni/Fe-based oxygen electrocatalysts

**DOI:** 10.1038/s41467-021-25811-0

**Published:** 2021-09-22

**Authors:** Wenchao Wan, Yonggui Zhao, Shiqian Wei, Carlos A. Triana, Jingguo Li, Andrea Arcifa, Christopher S. Allen, Rui Cao, Greta R. Patzke

**Affiliations:** 1 Department of Chemistry, University of Zurich, Winterthurerstrasse 190, CH-8057 Zurich, Switzerland; 2 School of Chemistry, Resource and Environment, Leshan Normal University, 614000 Leshan, China; 3 Empa, Swiss Federal Institute for Materials Science and Technology, Ueberlandstrasse 129, CH-8600 Dübendorf, Switzerland; 4 Electron Physical Science Imaging Center, Diamond Light Source Ltd, Didcot, Oxfordshire OX11 0DE UK; 5 Department of Materials, University of Oxford, Oxford, OX1 3HP UK; 6 Stanford Synchrotron Radiation Lightsource, SLAC National Accelerator Laboratory, Menlo Park, CA 94025 USA

**Keywords:** Chemistry, Catalysis, Catalytic mechanisms

## Abstract

Single-atom catalysts with maximum metal utilization efficiency show great potential for sustainable catalytic applications and fundamental mechanistic studies. We here provide a convenient molecular tailoring strategy based on graphitic carbon nitride as support for the rational design of single-site and dual-site single-atom catalysts. Catalysts with single Fe sites exhibit impressive oxygen reduction reaction activity with a half-wave potential of 0.89 V vs. RHE. We find that the single Ni sites are favorable to promote the key structural reconstruction into bridging Ni-O-Fe bonds in dual-site NiFe SAC. Meanwhile, the newly formed Ni-O-Fe bonds create spin channels for electron transfer, resulting in a significant improvement of the oxygen evolution reaction activity with an overpotential of 270 mV at 10 mA cm^−2^. We further reveal that the water oxidation reaction follows a dual-site pathway through the deprotonation of *OH at both Ni and Fe sites, leading to the formation of bridging O_2_ atop the Ni-O-Fe sites.

## Abstract (web summary)

The development of high performance dual-site single-atom catalysts is a promising research direction. Here, the authors report structural dynamics of dual-site nickel-iron single-atom oxygen electrocatalysts under reaction conditions, and proposes a dual-site pathway for the water oxidation reaction.

## Introduction

The exploration of green and high-efficiency techniques to meet the current energy shortage and climate change is extremely urgent^1^. The electrocatalytic oxygen evolution reaction (OER) and oxygen reduction reaction (ORR) are two very important processes for sustainable energy conversion and storage^2,3^. Although massive efforts have been directed on the development of catalysts for these two reactions over the past decades, current studies are still struggling with low activities, instabilities, and a lack of in-depth mechanistic understanding.

Single-atom catalysts (SACs) as a dynamically growing heterogeneous catalyst class keep attracting great research interest due to their combined features of fully exposed active sites and recyclable applications that are derived from both heterogeneous and molecular catalysts, respectively^4–10^. Among all of the current SACs, carbon-based representatives are considered especially promising^4,11–14^. On the one hand, the layered structures of graphene nanosheets provide an ideal platform for mechanistic studies due to their nearly complete exposure of the active centers^15^. This permits access to the actual dynamics of the structure and chemical states of these catalysts under operational conditions. On the other hand, their abundant carbon sources and straightforward syntheses render SACs quite accessible for industrial applications^16^. In-depth studies have revealed that carbon-based SACs, especially those with Fe centers (Fe-SACs), exhibit excellent ORR performance compared with other catalysts containing transition metal centers due to their stronger chemical adsorption ability toward O_2_ molecules^17^.

However, the OER performances of SACs are still less satisfactory compared with their conventional catalyst counterparts, such as oxides, hydroxides, or alloys^18^. Therefore, a complete understanding of their OER mechanisms and rational design of SACs with both high ORR and OER performance remains challenging. Due to their analogies to molecular catalysts, many studies of SACs are widely exploring related principles. For instance, a previous study showed that nonmetal coordinating atoms also participate in the OER, which provides an alternative strategy to improve the OER performance of SACs^19^. The enhancement of SAC activity through regulation of their coordination has been reported in previous works^20,21^. Alternatively, the introduction of a secondary metal type has been emerging as a promising research line to obtain SACs with high OER activities. It is evident that the formation of bridging Co-O-Fe bonds is the key step to improve the OER performance^22^.

To the best of our knowledge, a comprehensive, in-depth understanding of the role of each individual metal species during the OER reaction has not been obtained in previous works. Moreover, as one of the most typical examples, NiFe-based dual-site SACs have not been explored with respect to monitoring the dynamic formation of Ni-O-Fe bonds and understanding their kinetic contribution to the OER. Furthermore, the study of dual-site SACs for OER is still in its infancy, and the isolation of two different metal types on the same support remains challenging, e.g., due to the widespread application of pyrolysis processes with little control over atomic arrangements. As a consequence, the OER mechanisms of dual-site SACs have barely been investigated.

In this work, we present new experimental and theoretical mechanistic insight into the OER pathways of dual-site NiFe SACs with an impact on their performance optimization. To this end, we first introduce a convenient molecular tailoring strategy based on graphitic carbon nitride (g-C_3_N_4_) as support for the design of single- and dual-site SACs with excellent ORR and OER activities. Ni, Co, Fe single sites and Ni-Fe dual sites on N-doped graphene nanosheets were successfully isolated through a controllable pyrolysis protocol, referred to as Ni-CNG, Co-CNG, Fe-CNG, and NiFe-CNG. We systematically studied the chemical interactions of the precursors and proposed a two-step growth mechanism, which provides a widely applicable pathway for the design and synthesis of SACs. A wide range of techniques, such as X-ray absorption spectroscopy (XAS) and high-angle annular dark-field scanning transmission electron microscopy (HAADF-STEM), were applied to identify their compositions and structures. SACs with single Fe sites excelled through remarkable ORR activity with an impressive half-wave potential of 0.89 V vs. RHE.

Most importantly, our systematic mechanistic studies based on in situ XAS measurements of both single-site Ni and Fe SACs together with the dual-site NiFe SAC reveal that the OER-inactive Ni sites are more favorable to promote the structural reconstruction under OER conditions compared to Fe sites, leading to the formation of highly active Ni-O-Fe OER sites with an overpotential of 270 mV at 10 mA cm^−2^. Density functional theory (DFT) calculations further indicate that the spin differences at the Ni and Fe sites in the in situ formed Ni-O-Fe bonds create spin channels for electron transfer during the OER. We newly identified this effect as the ultimate origin of the enhanced OER performance. Further DFT calculations of the reaction pathways combined with the experimental results show that the increased oxidation states of both Ni and Fe species opened up a dual-site OER pathway via the key steps of double *OH deprotonation at both Fe and Ni sites and the formation of a bridge O_2_ atop the Ni-O-Fe sites. Based on a new versatile strategy to synthesize SACs with both single-site and dual sites, our combined approach of in situ XAS monitoring along with DFT calculations offers profound insight into the reaction mechanism of Ni-Fe dual-site OER SACs.

## Results and discussion

### Synthesis and structural characterization

**Fig. 1:**
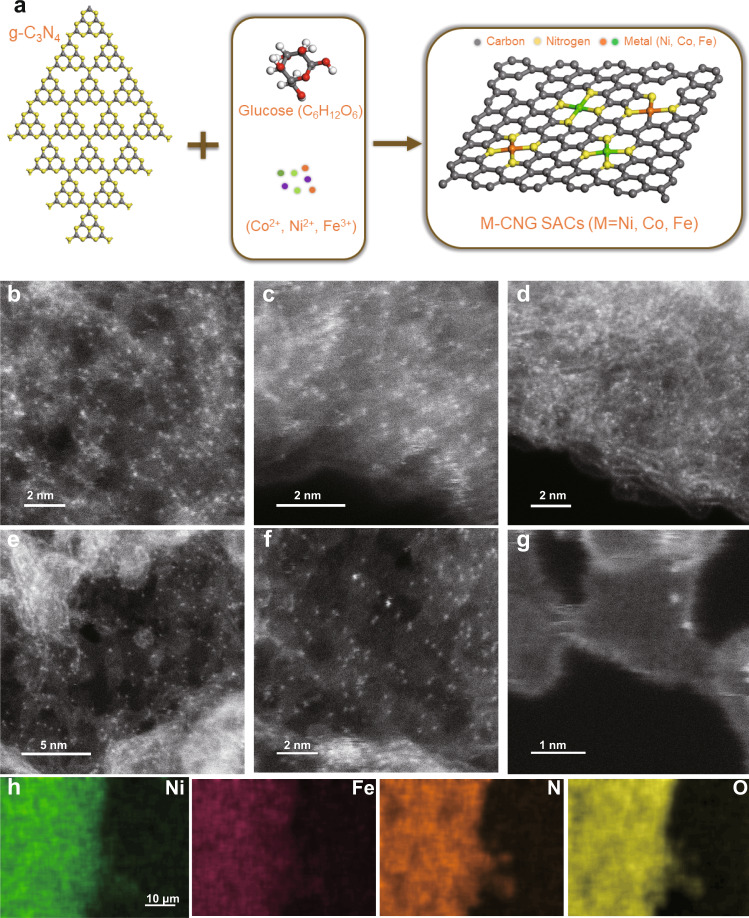
Fabrication route and morphological characterization. **a** Scheme of molecular tailoring synthesis of graphene-based SACs using g-C_3_N_4_ and glucose as precursors. **b**–**d** HAADF-STEM images of **b** Ni-CNG, **c** Co-CNG, and **d** Fe-CNG. **e**–**g** HAADF-STEM images of NiFe-CNG at different magnifications. The uniformly distributed bright dots represent metal atoms. **h** EDS mapping of NiFe-CNG recorded along with the SEM images.

The SACs were fabricated via a simple molecule-based tailoring strategy using glucose and bulk g-C_3_N_4_ as stabilizer and support, respectively (Fig. 1a). The detailed synthetic protocol is presented in the Supporting information. The metal contents of the as-prepared SACs were determined to be 7.5, 7.6, 10.5, and 7.5 wt% for Ni-CNG, Co-CNG, Fe-CNG, and NiFe-CNG (CNG = N-doped graphene nanosheets prepared with glucose), respectively, by inductively coupled plasma triple quadrupole mass spectrometry (ICP-MS). These determined metal loadings are higher than for most of the recently reported SACs. X-ray photoelectron spectroscopy (XPS) indicates that the corresponding surface metal contents are 5.6, 6.1, 6.6, and 5.6 wt% for Ni-CNG, Co-CNG, Fe-CNG, and NiFe-CNG, respectively (Supplementary Table 6). High metal content provides not only more actives sites but also more reliable signals during in situ measurements, such as XAS.

The structure of the catalysts was first investigated with Raman spectroscopy and powder X-ray diffraction (PXRD) measurements (Supplementary Figs. 1–3). Raman spectra reveal that the signals of bulk g-C_3_N_4_ were completely absent after the pyrolysis process and were replaced with two new bands located at around 1350 and 1620 cm^−1^ (Supplementary Fig. 2), which correspond to the D and G bands in graphene-like materials, respectively^23^. A related trend was observed for the corresponding PXRD patterns. The featured peaks of g-C_3_N_4_ completely vanished after the pyrolysis reaction without the emergence of new diffraction signals, which indicates the amorphous and disordered features of the SACs. The amorphous features also suggest that the metal species are atomically dispersed (Supplementary Figs. 1 and 3). The pyrolysis process was further investigated with transmission electron microscopy (TEM) (Supplementary Figs. 4–8), and representative images suggest that the mixture of g-C_3_N_4_, glucose, and metal ions was completely converted into graphene nanosheets without generating metal nanoparticles. To directly identify the atomic dispersion of metal atoms in the graphene nanosheets, as-prepared samples were further inspected with HAADF-STEM. As presented in Fig. 1b–g and Supplementary Figs. 9–11, the metal atoms (bright dots) are homogeneously dispersed on graphene layers. Energy-dispersive X-ray spectroscopy (EDS) further confirmed the uniform composition and dispersion of metal, N, and O atoms in all the samples (Fig. 1h and Supplementary Figs. 12–15). Some graphene layer images show an overlap of a few graphene layers (Supplementary Figs. 16–17). All the above results indicate that the metal atoms were successfully isolated and implanted into graphene lattices.

To achieve an in-depth understanding of the growth process of SACs, a series of control experiments were carried out. The samples prepared without glucose show that metal ions were completely aggregated into nanoparticles after the pyrolysis process at 900 °C (Supplementary Figs. 18–20). Interestingly, the bulk g-C_3_N_4_ was converted into carbon nanotubes (CNTs) with metal nanoparticles encapsulated into CNTs when reacted with Ni and Co ions (Supplementary Figs. 18 and 19), while the mixture containing Fe ions adopted a carbon ball-like morphology throughout with Fe nanoparticles inside (Supplementary Fig. S20). PXRD patterns and Raman spectra demonstrate that all the products obtained in the absence of glucose contain both the respective metal alloys and graphene layers (Supplementary Figs. 21 and 22). Similar results have been reported in a previous study^24^. The above results show that glucose is indispensable for the formation of SACs. The function of glucose can be explained by its chelating effect. Consequently, the glucose molecules chelate the metal ions and anchor them on the surface of g-C_3_N_4_, thereby preventing the metal atoms from aggregation (Supplementary Figs. 23–25)^25,26^. On the other hand, samples prepared with only g-C_3_N_4_ and glucose show perfect graphene features as confirmed by TEM images and Raman spectra (Supplementary Figs. 26 and 27). However, bare g-C_3_N_4_ without any additives was completely decomposed after the pyrolysis reaction under the same conditions, suggesting that glucose also plays a crucial role in the formation of graphene nanosheets. The pyrolysis of bare glucose resulted in a completely sintered product (Supplementary Fig. 28), further indicating that glucose acts not only as a type of chelator to separate the metal atoms and as a stabilizer for the formation of single atoms but also as a “catalyst” for the formation of graphene from g-C_3_N_4_. Based on the above results, we, therefore, propose a two-step formation mechanism. In the first step, the metal ions are chelated by the glucose molecules in water to form a homogeneous dispersion onto g-C_3_N_4_. In the second step, g-C_3_N_4_ anchored with metal atoms chelated by glucose is further converted into N-doped graphene under the high-temperature pyrolysis process conditions, with glucose acting as a “catalyst”. During this step, the metal atoms are further coordinated by the nitrogen atoms of the N-doped graphene. It is worth noting that the formation process and performance of the SACs are very sensitive towards the applied synthesis temperature (Supplementary Fig. 29).

**Fig. 2:**
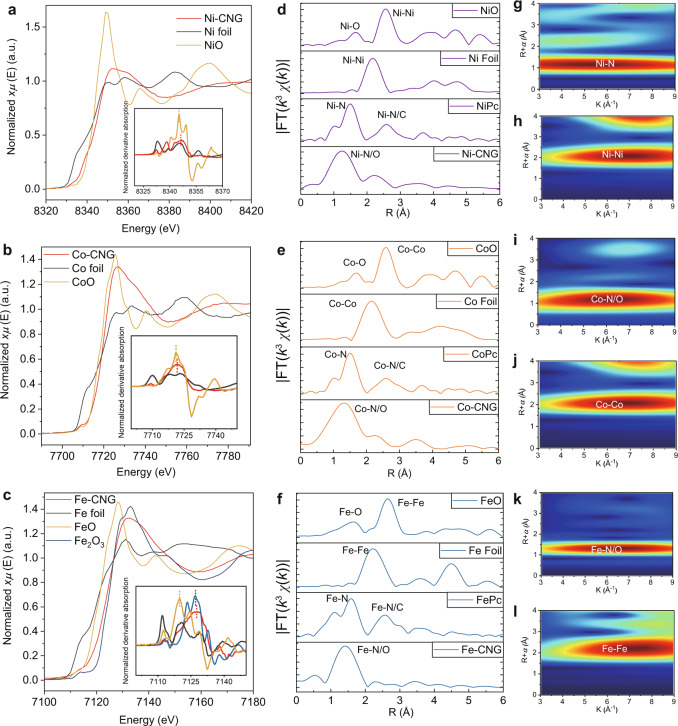
Structural characterizations based on XAS. **a**–**c** K-edge XANES and **d**–**f** Fourier transform K-edge EXAFS spectra of Ni-CNG, Co-CNG, Fe-CNG, and their reference samples. **g**–**l** Wavelet transforms of the *k*^3^-weighted EXAFS signals of Ni-CNG, Co-CNG, and Fe-CNG and of their corresponding metal foil references.

To identify the local structures and oxidation states of the SACs, Ni, Co, and Fe K-edge XAS spectra were recorded. As illustrated in Fig. 2a, the absorption edge of Ni-CNG is very close to NiO, but far away from that of Ni foil, and the first derivative of the X-ray absorption near-edge structure (XANES) spectra also shows that the peak position of Ni-CNG is overlapping with NiO (Fig. 2a, inset). This indicates that the average valence state of Ni species in Ni-CNG is Ni^2+^. Similarly, by comparing the XANES spectra and the first derivative of Co^2+^, the average valence state of Co species in Co-CNG can be assigned as Co^2+^ (Fig. 2b). Both the K-edge XANES spectrum and the first derivative of Fe-CNG demonstrate that the main oxidation state of Fe species in Fe-CNG is Fe^3+^ (Fig. 2c). The oxidation states of the samples were also confirmed with XPS (Supplementary Fig. 31). The Ni 2*p* spectrum of Ni-CNG contains a rather narrow spin–orbit doublet that is characteristic of the Ni 2*p*_3/2_ core level at 855.2 eV. The absence of multiple splitting and of strong satellite peaks indicates that the Ni species are diamagnetic, which is compatible with a square planar geometry, and an oxidation state of +2 of the metal center^27,28^. In comparison, the Fe 2*p* and Co 2*p* signals of Fe- and Co-CNG (3/2 core levels of 710.3 and 780.6 eV, respectively) are substantially broader and exhibit more intense satellite features, suggesting the paramagnetic character of Co and Fe species^29^. Together with the above analyses of XANES spectra (Fig. 2b), we provide convincing evidence that Fe^3+^ is the main oxidation state in Fe-CNG, while Co^2+^ is the dominant state in Co-CNG, although the presence of a slight fraction of Co^3+^ cannot be completely excluded.

The Fourier-transform extended X-ray absorption fine structure (FT-EXAFS) spectra of Ni-CNG, Co-CNG, and Fe-CNG are very similar with respect to the main peaks located at around 1.3–1.45 Å together with the shoulder peaks at around 2.2–2.4 Å (Fig. 2d–f). They can be attributed to M-N/O and M-C (M = Ni, Co, Fe) bonds, respectively. This is also evident from the comparison of the FT-EXAFS spectra with their (Ni, Co, and Fe) phthalocyanine analogs and from previous carbon-based SAC research^30^. The comparison with respective foil and oxide references shows that there are no apparent M-M bonds in all of the samples, suggesting that Ni, Co, and Fe species are atomically isolated by the surrounding N and C atoms. The above results are in line with the analyses of their wavelet transform (WT)-EXAFS spectra. In the WT-EXAFS spectra, only one intensity maximum assigned to the first scattering shell for all samples was observed (Fig. 2g–l), and no further scattering signals were detected beyond 2 Å, while, in their foil references, the typical M-M scattering shells were detected around 2.1 Å. This further confirms the isolated features of the metal species in the samples.

The survey and high-resolution N 1*s* and O 1*s* spectra of Ni-CNG, Co-CNG, Fe-CNG, and NiFe-CNG and of the metal-free reference sample (CNG) are presented in Supplementary Figs. 30 and 32. The results suggest that the samples not only contain nitrogen coordinated metal centers but also a non-negligible amount of oxygen species (Supplementary Table 6). The N 1*s* spectrum of CNG consists of a broad peak envelope, whose shape closely resembles that of previous N-doped graphitic materials^31^. In particular, two major peaks centered at 398.2 and 400.7 eV, respectively, are ascribed to pyridine- and pyrrole-like nitrogen^32,33^, although a wide variety of nitrogen-containing functional groups can also contribute to the N 1*s* peak envelope^34^. All the N 1*s* spectra of metal-containing samples show variations in terms of the relative intensity of the two major peaks, and a concurrent increase of intensity in the valley between these two peaks. This is in line with the coordination of metal ions by N-donor ligands (Supplementary Fig. 32a). The O 1*s* spectrum of the CNG exhibits an unresolved broad signal centered at about 532.2 eV (Supplementary Fig. 32b), which has been observed in previous investigations^35,36^. The addition of Fe species to the sample (Fe-CNG) results in a significant broadening of the O 1*s* envelope toward the lower-BE side of the spectrum. This phenomenon is quite similar, although less pronounced, for Co-CNG, while no obvious changes are observed in Ni-CNG. This suggests that iron and cobalt ions interact with O-donor ligands that are present in the graphitic material^37^. However, interpretation of the O types through XPS only is rather difficult due to the substantial overlap of peaks arising from attached O ligands.

To obtain insight into the origins of the structural signatures of the first coordination shell, calculated EXAFS spectra of M-N4, M-N3-O, M-N2-O2, M-N-O3, M-O4, and M-N4-O2 (O_2_ molecule adsorbed atop the metal center) configurations were employed for comparison with the experimental results. As demonstrated in Supplementary Figs. 33–35, the partial replacement of M-N bonds with M-O bonds leads to the presence of a broad peak in the first coordination shell, while the shoulder peak near the first peak gradually disappears. These results show that the experimental EXAFS spectra of Co-CNG and Fe-CNG atoms are very close to the simulated model for metal centers coordinated by both O and N atoms, while Ni-CNG is closely related to the Ni-N4 model. In combination with the above XPS results and the calculated EXAFS spectra, we, therefore, propose that Ni-CNG, Co-CNG, and Fe-CNG most likely contain the Ni-N4, Co-N3-O, and Fe-N2-O2 moieties, respectively. We further used the proposed models to fit the FT-EXAFS spectra of all these three samples, and all the models agree well with the experimental data (Supplementary Table 8, and Supplementary Figs. 59 and 61).

The local structure and composition of dual-site NiFe-CNG were also identified with XAS and XPS. XPS spectra suggest that the metal oxidation state and coordination environment are very close to bare Ni-CNG and Fe-CNG (Supplementary Figs. 31 and 32). The K-edge XANES and the Ni and Fe 3*p* XPS spectra indicate that the main oxidation states of Ni and Fe species in NiFe-CNG are Ni^2+^ and Fe^3+^, respectively (Supplementary Figs. 31 and 36). The WT-EXAFS spectra and FT-EXAFS fitting results together with the analyses of O 1*s* and N 1*s* spectra further manifest that the main scattering peaks located at around 1.3–1.45 Å account for the Ni-N and Fe-N/O bonds, respectively (Supplementary Figs. 32, 37, and 38).

### Electrocatalytic ORR and OER activities

**Fig. 3:**
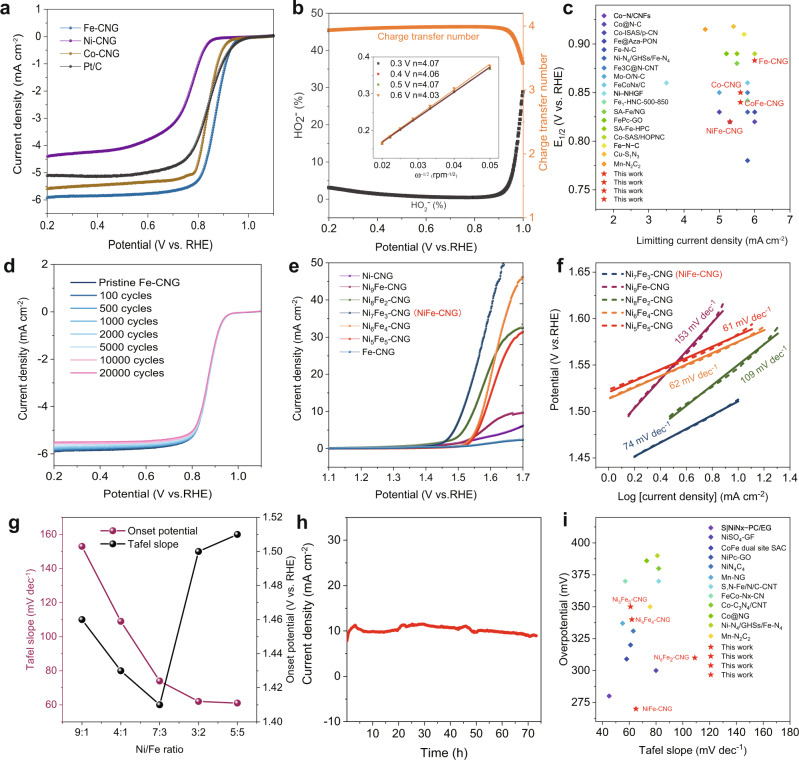
Electrochemical ORR and OER performances. **a** ORR polarization curves in O_2_-saturated 0.1 M KOH at a scan rate of 5 mV s^−1^. **b** HO_2_^−^ yield for ORR and the number of transferred electrons with K–L plots (inset). **c** Summary of ORR performance of recent representative SACs with their corresponding half-wave potentials and limiting current densities at 1600 r.p.m. **d** LSV curves after 100, 500, 1000, 2000, 5000, 10,000, and 20,000 CV cycles. **e** OER polarization curves of NiFe-CNG with different Ni/Fe ratios, and **f** the corresponding Tafel plots. **g** Influence of Ni/Fe ratios on the Tafel slope and onset potential of OER. **h** OER stability test of NiFe-CNG loaded on Ni foam. **i** Recent OER performances of SACs with their corresponding overpotentials at a current density of 10 mA cm^−2^ and Tafel slope values.

It has been widely reported that carbon-based SACs are favorable for electrocatalytic applications^38,39^. To evaluate the performance of the SACs, electrocatalytic ORR was carried out for all the prepared samples. First, measurements were conducted in both O_2_ and Ar-saturated 0.1 M KOH solution (Supplementary Fig. 39a–c). In comparison with the cyclic voltammetry (CV) curves recorded in Ar-saturated electrolyte, well-defined cathodic peaks were observed for all catalysts in O_2_-saturated electrolyte, indicating that all the samples are active toward ORR. The linear sweep voltammetry curves obtained by using the rotating disk electrode (RDE) technique further demonstrate that Fe-CNG exhibits the best ORR activity among all the measured SACs with an extremely high half-wave potential of 0.89 V vs. RHE and a limiting current density close to 6.0 mA cm^−2^ (Fig. 3a). The ORR activity of Fe-CNG outperforms all other reference samples presented in this manuscript (Supplementary Fig. 40) and the commercial 20 wt% Pt/C catalyst as well as many up-to-date graphene-based SACs listed in Fig. 3c and Supplementary Table 10. Interestingly, the ORR performance of Fe-CNG is even superior to the dual-site NiFe-CNG SACs (Supplementary Fig. 39d) and a newly prepared CoFe-CNG reference (Supplementary Fig. 41 shows the atomic dispersion of Co and Fe centers). This implies that the isolated Fe sites are the dominating active species for ORR^40^. The high ORR performance of Fe sites is related to their stronger bonding affinity of Fe centers toward O_2_ molecules^41,42^, which is in line with previous literature^19,43^. To further elucidate the ORR process, Koutecky–Levich (K–L) equation and rotation ring-disk electrode (RRDE) tests were carried out. The number of transferred electrons for Fe-CNG is very close to 4.0, and the corresponding peroxide (H_2_O_2_) yield is strikingly suppressed to less than around 2.0% (Fig. 3b), suggesting a four-electron pathway of the reaction and excellent selectivity for reducing O_2_ to OH^−^. The ORR kinetics of Fe-CNG was evaluated by RRDE tests at different rotation speeds ranging from 400 to 2500 r.p.m. (Supplementary Fig. 42a). The K–L equation shows very good linear and parallel characteristics (Fig. 3b inset), implying that the ORR is a first-order reaction. The corresponding Tafel slopes show that Fe-CNG has the smallest Tafel slope of 57 mV dec^−1^, indicating its fast ORR kinetics (Supplementary Fig. 42b). The stability of Fe-CNG for ORR was further investigated by measuring the RDE polarization curves for 20,000 CV cycles. The result shows an almost negligible decrease in the half-wave potential, and only a slight decrease in the limiting current (Fig. 3d). XANES and FT-EXAFS spectra of Fe-CNG after the long-term stability test demonstrate that the oxidation state and local structure are well preserved (Supplementary Fig. 43), further suggesting excellent stability toward ORR. We also tested the promising Zn–air battery application potential of Fe-CNG and NiFe-CNG (Supplementary Fig. 44).

In stark contrast, the single-site SACs (Ni-CNG and Fe-CNG) exhibit almost no activity toward OER (Supplementary Fig. 45). The fact that many graphene/carbon-based SACs with single transition metal sites are chemically inactive toward OER has been gradually acknowledged by researchers. Massive efforts have thus been made to improve the OER performance of SACs, such as the regulation of coordination environments of the metal centers, interaction of diatomic metal sites, etc.^44^. Inspired by the recent development of NiFe coordination polymers for high OER performance^45^, we here developed the NiFe-CNG dual-site SACs with the goal of notably improving the OER performance of the above single-site SACs. As expected, the addition of Fe species leads to a significant improvement of the OER activity of Ni-CNG (Fig. 3e). Interestingly, the incorporation of Fe species demonstrates a typical “volcano”-type correlation with the OER activity (Fig. 3g), which agrees well with previous works and recent studies on NiFe-based catalysts^45,46^. Specifically, the catalyst with the nominal Ni/Fe molar ratio of 7:3 displays the best activity with an overpotential of 270 mV at the current density of 10 mA cm^−2^ (Fig. 3e and Supplementary Fig. 46), which is superior to the CNG, M-CN (M = Ni, Co, Fe) series and most carbon-based SACs (Fig. 3i and Supplementary Fig. 40). Moreover, the chronoamperometry measurements show that the catalyst retains its pristine catalytic activity for more than 72 h at the current density of 10 mA cm^−2^ without any distinct drop (Fig. 3h and Supplementary Table 11). Further TEM images after the long-term stability test show that the morphology of NiFe-CNG was well preserved and no additional nanoparticles were formed after the OER (Supplementary Fig. 47). The ICP-MS measurements of the electrolyte before and after the stability test show that no metal leaching took place (Supplementary Table 7). However, the Tafel slopes for all catalysts show a continuously decreasing trend from around 150 to 60 mV dec^−1^ with increasing the Ni/Fe molar ratios from 9:1 to 1:1, along with a shift of the reaction order from first to second (Fig. 3f–g). This indicates that the OER kinetics can be regulated by optimizing the Ni/Fe ratio. The Tafel slopes of all the dual metal catalysts are significantly smaller than these of Ni-CNG and Fe-CNG, indicating that the mixture of Ni and Fe species can dramatically boost the OER kinetics. The different changes in onset potential and Tafel slope indicate that the Fe species may play different roles in the reaction thermodynamics and kinetics of OER. To investigate the kinetics during OER, in situ electrochemical impedance spectroscopy (EIS) studies were conducted (Supplementary Fig. 48). The results show that all the catalysts display a charge transfer process related to surface intermediates at a low operating potential range. However, at high potentials, Ni-CNG and Fe-CNG transform to a combination of relaxation components of processes involving double layer and surface intermediates, while NiFe-CNG only undergoes a single charge transfer process related to double-layer capacitance. The difference in the kinetics indicates that the synergistic effect of Ni and Fe atoms is more favorable for the overall OER process (see details in Supplementary Fig. 48). In the following section, we proceed to evaluate in situ XAS measurements along with DFT simulations to further investigate the interaction of Fe and Ni species during OER.

### In situ XAS studies of NiFe-CNG under OER conditions

**Fig. 4:**
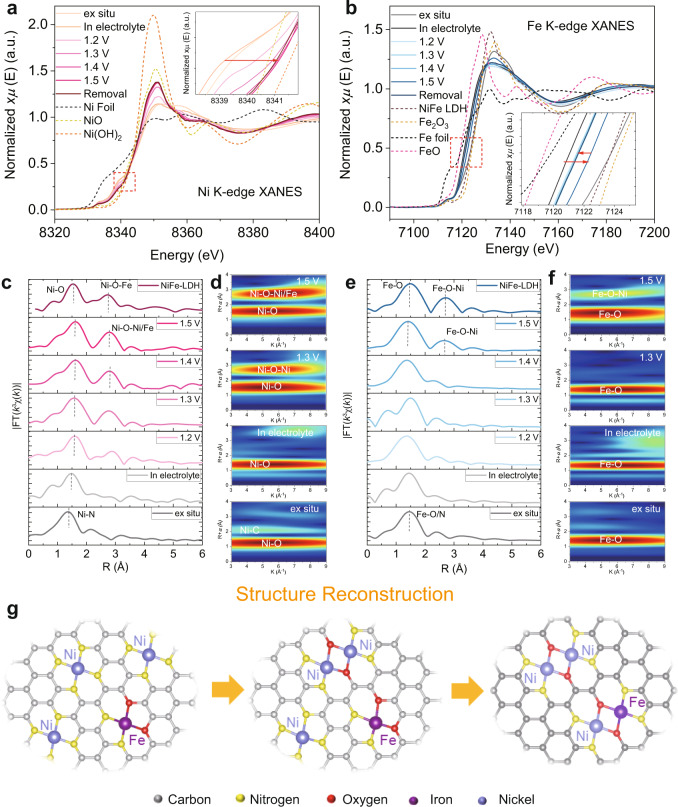
In situ XAS of NiFe-CNG under OER conditions. Ni, Fe K-edge. **a**, **b** XANES and **c**, **e** FT-EXAFS spectra of NiFe-CNG under different applied potentials, and the corresponding XANES and FT-EXAFS spectra of their reference samples. **d**, **f** Extracted wavelet transforms of  the *k*^3^-weighted EXAFS signals of NiFe-CNG under applied potentials. **g** Structural reconstruction of NiFe-CNG during OER from separate Ni and Fe centers to Ni-O-Fe/Ni moieties.

To track the structural evolution and the chemical behavior of Ni and Fe species during OER, in situ XANES and FT-EXAFS spectra were further recorded under various electrochemical conditions, starting with Ni and Fe K-edge XANES spectra of NiFe-CNG and their reference samples. For the Ni K-edge XANES (Fig. 4a), when the applied potentials increased from 1.2 up to 1.5 V (vs. RHE), the rising-edge positions were continuously shifted to higher energy values, implying a distinct increase of the oxidation state of Ni species in NiFe-CNG (Fig. 4a). The shift of the rising-edge position slightly recovered after the removal of all applied potentials, which further indicates the reversibility of the Ni species with respect to changes in the oxidation state. The higher oxidation state is also evident from the increase of the white line intensity^47–49^. The Fe K-edge XANES (Fig. 4b) underwent only a slight energy shift with increasing applied potential, which is similar to previous in situ XAS measurements on Fe-containing OER catalysts^50^. The energy shift of the rising edge caused by the electrolyte may be related to the electrostatic interaction between catalyst and electrolyte or by the adsorption of OH^−^^51–53^. However, the removal of the potential led to a slight recovery of the positions of both absorption edges, implying that the changes in oxidation state are partially reversible (Fig. 4b, inset).

To further probe the structural changes around the Ni and Fe species in the catalyst, FT-EXAFS spectra were recorded at both Ni and Fe K-edges (Fig. 4c, e). For the FT-EXAFS spectra at the Ni K-edge, the distance of the first coordination shell slightly increased from 1.37 to 1.48 Å after immersion into the electrolyte, which may be due to the formation of Ni-OH/H_2_O moieties in contact with the electrolyte^47^. The Ni-OH/H_2_O species are also evident from the FT-infrared (FTIR) spectra in Supplementary Fig. 49, in which the new peaks appearing at 500–800 cm^−1^ after the OER are ascribed to the stretching vibrations of M-O (M = Ni, Fe) groups^54,55^. The distance of the first coordination shell further increased when the applied potential was increasing. This is because a higher extent of Ni-O coordination was formed under reaction conditions as shown in Supplementary Table 9^56^. Interestingly, another new peak appeared at around 2.78 Å when the applied potential crossed the threshold of 1.3 V (vs. RHE). Similar phenomena are also present in the WT-EXAFS spectra, in which new scattering signals emerged at around 2.78 Å at an applied potential >1.3 V vs. RHE (Fig. 4d). We further recorded the in situ EXAFS spectra of NiFe-CNG at the Fe K-edge (Fig. 4e). Similarly, the oxygen coordination around Fe centers steadily increased while exposing the catalyst to the electrolyte and applying potentials (Supplementary Fig. 48 and Table 9), which can be attributed to the formation of Fe-OH/H_2_O species^57^. However, the signal at around 2.78 Å was present only when the potential was raised >1.5 V (vs. RHE) (Fig. 4f), which is in line with the WT-EXAFS spectra (Fig. 4f). This suggests that the new bonds are much more difficult to form at the Fe sites compared to those at the Ni atoms.

The newly arising signals from both Ni and Fe K-edge are very close to the Ni-O-Fe bonds in NiFe-LDH, which are considered as the real active sites for OER. Therefore, we added the FT-EXAFS (Fig. 4c, e) and WT-EXAFS spectra (Supplementary Fig. 50) of newly prepared NiFe-LDH for comparison. The new signals at around 2.78 Å from both Ni and Fe K-edge are in good agreement with the Ni-O-Fe bonds in NiFe-LDH. The formation of M-O-M bonds (M = Ni, Co, Fe) in transition metal-based materials for OER in the alkaline electrolyte has been widely accepted^58,59^. Considering the alkaline environment of the OER, we suppose that the new peaks at 2.78 Å are the Ni-O-Fe bonds. We thus created a series of models and calculated their WT-EXAFS spectra to compare with NiFe-CNG under the applied potential of 1.5 V (vs. RHE) (Supplementary Figs. 51–53). The results show that the new peaks are well corresponding to the Ni-O-Fe bonds created in our models, especially when the OH^−^ species are adsorbed at the Ni and Fe centers (Supplementary Fig. 53). Moreover, the proposed model (Supplementary Fig. 53) was used to fit the  FT-EXAFS spectra of NiFe-CNG at the applied potential of 1.5 V (vs. RHE), and the results show very good consistency (Supplementary Figs. 54 and 55 and Table 9). To exclude the formation of NiFe oxyhydroxides, we further recorded TEM and HAADF-STEM images and Raman and XPS spectra of NiFe-CNG after OER (Supplementary Figs. 77–81). The TEM and HAADF-STEM images show that the atomic dispersion of metal atoms is well preserved, and no apparent phase segregation of larger Fe or Ni species was observed. This is further confirmed by the Raman spectra of the postcatalytic samples, where the characteristic peaks of oxyhydroxides were not observed (Supplementary Fig. 80). Furthermore, the N and O 1*s* XPS spectra of postcatalytic NiFe-CNG indicate that both M-O and M-N bonds are present in the catalyst, which suggests that the newly formed Ni-O-Fe moieties are connected with the carbon lattice through M-N bonds. Based on the above discussion, we propose three possible pathways of the structural reconstruction process. First, metal atoms at the edges and defect-rich areas with unsaturated coordination environments could further bond to OH^−^ species during OER and proceed to form bonds with the adjacent Ni/Fe sites (Supplementary Fig. 82). Second, metal atoms dispersed on the carbon layers are very close to each other, with many of them even within a 3 Å range, so that it is quite reasonable to assume that the metal atoms at defective areas move towards their neighboring metal atoms to establish M-O-M bonds. Third, the catalysts are composed of small carbon fragments as shown in the STEM images (Supplementary Fig. S82). The applied voltage during OER usually leads to the movement of these carbon fragments, which may also promote the formation of M-O-M bonds, especially at the edges of two fragments in the presence of OH^−^ species in the electrolyte. A detailed discussion of these pathways is also presented in Supplementary Note 2. The above results imply that the new signal is assigned to Ni-O-Ni/Fe bonds, and that the local coordination of Ni and Fe centers underwent a structural reconstruction during the water oxidation reaction. However, due to the similar atomic numbers of Ni and Fe, the accurate distinction of Ni-O-Fe, Ni-O-Ni, and Fe-O-Fe bonds from XAS remains challenging.

To further differentiate the types of M-O-M bond and to investigate the origin of the enhanced OER performance, in situ XAS spectra of bare Ni-CNG and Fe-CNG were recorded as references under the same experimental conditions. The oxidation states of Ni and Fe species were investigated through recording of the XANES spectra with the applied potentials raised from open-circuit voltage to 1.6 V (vs. RHE). For Ni-CNG, the spectra show a similar trend as for the Ni K-edge in NiFe-CNG, where the rising edges continuously increased to higher absorption energies with higher applied potentials (Supplementary Fig. 56). It is very impressive that the XANES spectra of Fe-CNG show almost no shift even when the applied potential reached 1.6 V (vs. RHE), with the exception of a slight shift to lower energies when exposed to the electrolyte (Supplementary Fig. 57). The chemical inactivity of the XANES spectrum indicates that the Fe species in bare Fe-CNG are rather stable with respect to both chemical state and local structure during OER compared with the Fe species in NiFe-CNG. The distinction, on the other hand, strongly suggests that the Fe atoms surrounded by Ni species in NiFe-CNG are more dynamic, leading to enhanced OER performance.

The EXFAS spectra were further recorded to investigate this structural evolution. In the Ni K-edge FT-EXAFS and WT-EXAFS spectra of Ni-CNG, an additional Ni-O-Ni bond was observed at around 2.78 Å with the increase of the applied potential. The coordination changes around Ni species in bare Ni-CNG are very similar to those in NiFe-CNG (Supplementary Figs. 58 and 59). On the other hand, Fe K-edge FT-EXAFS and WT-EXAFS spectra of Fe-CNG show completely different results compared with those of NiFe-CNG. Fe-O-Fe bonds were not formed even if the applied potential was increased up to 1.6 V (vs. RHE) (Supplementary Figs. 60 and 61). Therefore, the coordination shell at approximately 2.78 Å in the Fe K-edge FT-EXAFS spectra of NiFe-CNG (Fig. 4e) is mainly assigned to Fe-O-Ni bonds. This means that a reconstruction of the coordination environment is more likely to occur around Ni atoms, leading to the formation of Ni-O-Ni/Fe bonds during the OER (Fig. 4g). The newly formed Ni-O-Fe bonds imply more active Fe species in NiFe-CNG when comparing the XANES spectra with those of bare Fe-CNG. It is worth noting that both bare Ni-CNG and Fe-CNG display almost no significant OER performance. Therefore, it is reasonable to believe that the significant improvement of the OER performance in NiFe-CNG arises from the formation of Ni-O-Fe sites. This contrasting behavior of Ni and Fe sites has not been reported in previous SACs and in other NiFe-based bulk catalysts, and these results provide a new perspective to understand the high performance and application potential of NiFe-based OER catalysts.

### DFT calculations and proposed reaction pathway

**Fig. 5:**
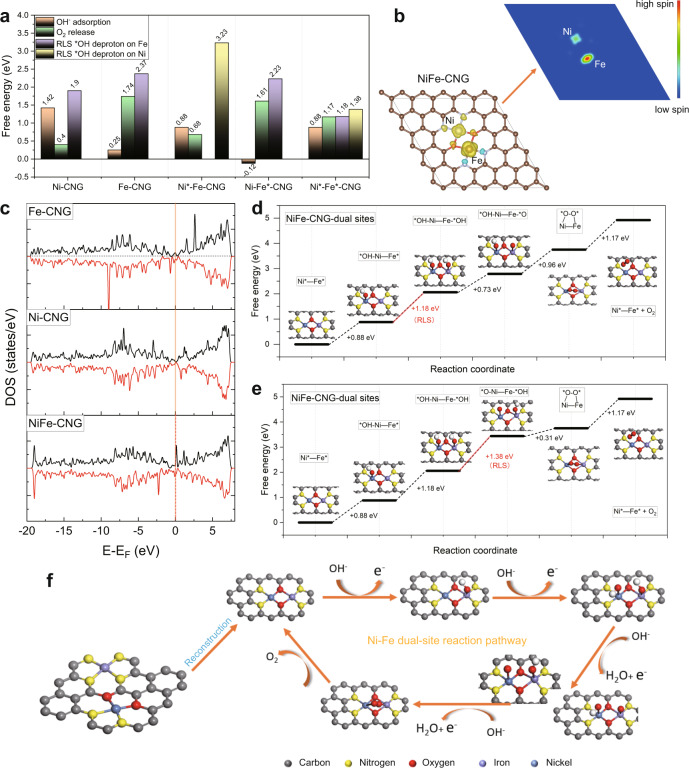
DFT simulations and proposed OER pathways. **a** Free energy comparison of OH^−^ adsorption, O_2_ release, and the RLS between different structural configurations. **b** Spin density pattern (left) and spin channel (right) of Ni and Fe sites in NiFe-CNG. The isosurfaces in yellow and blue represent spin-up and spin-down densities, respectively. **c** Density of states (DOS) of Fe-CNG, Ni-CNG, and NiFe-CNG. **d**, **e** Free energy diagrams of NiFe-CNG models toward OER at the Ni-Fe dual site. **f** Schematic illustration of the proposed overall OER pathway for the NiFe-CNG catalyst.

As mentioned above, the real active site of NiFe-CNG for the OER is the Ni-O-Fe moiety formed during structural reconstruction (Fig. 4g). To shed more light on the reaction pathways and to elucidate the influence of metal types on the electronic nature of the atomic sites for OER, DFT calculations were conducted for Ni-CNG, Fe-CNG, and NiFe-CNG. It is worth noting that all the models used for DFT simulations are based on the postcatalytic structures as shown in Supplementary Fig. 62. Based on the results of in situ XAS monitoring, the postcatalytic structures of NiFe-CNG and Ni-CNG were converted into Ni-Fe and Ni-Ni dual sites bridged with two oxygen atoms (Supplementary Figs. 54 and 59), respectively, while Fe-CNG retains the configuration of a single site due to the absence of a Fe-O-Fe signal in the in situ FT-EXAFS spectra (Supplementary Fig. 61). For all the models, we first considered the conventional single-site reaction pathways, which were widely reported in the previous literature^60^. The results manifest that OH^−^ adsorption at Fe sites is energetically more preferable than at Ni sites among all these three models. However, the deprotonation of *OOH to form O_2_ molecules is thermodynamically more favorable to occur at Ni sites (Fig. 5a). The difference in thermodynamic adsorption energies has been reported to be connected to the number of *d* electrons (N_*d*_) in the metal centers^19,61^. Based on the calculated energy barrier diagrams (Supplementary Figs. 63–66), we found that the free energy of the reaction-limiting step (RLS) for the Ni-CNG model is 1.9 eV, which is the lowest among all the models according to single-site reaction pathways. The unexpected result implies that the NiFe-CNG catalyst may not follow the single-site reaction pathway.

Previous works have reported that the synergy of Fe and Ni or Co atoms in NiFe- and CoFe-based hybrid catalysts could stabilize the OER intermediates (*OH, *O, and *OOH) on the metal sites, which accounts for the low overpotentials in the OER^62^. Moreover, it has been widely reported that catalysts with two different metal sites connected by bridging oxygen atoms follow a dual-site reaction pathway^63,64^. As we found here experimentally that Ni-O-Fe bonds were formed during OER (Fig. 4c, e), we propose that OER in the NiFe-CNG catalyst proceeds via the dual-site reaction mechanism.

DFT calculations for the dual-site process indicate that both Ni and Fe sites participate in the OER (Fig. 5d, e). The results demonstrate that the structure of the Ni-*O-O*-Fe with the bridging O_2_ atop connecting Fe and Ni atoms is thermodynamically more stable than that of O*-Ni-Fe-*O after the second *OH deprotonation process (Supplementary Fig. 67). This indicates that the two *O species originating from the *OH deprotonation step at Ni and Fe sites can immediately convert into Ni-*O-O*-Fe, and further generate O_2_ molecules. This is different from a nucleophilic attack of the second water via the *OOH intermediate, as occurring in the conventional single-site mechanism^65^. The two deprotonation processes of *OH on both Ni and Fe sites are also evident from the FTIR (Supplementary Fig. 49) and in situ XANES (Fig. 4a, b) spectra. The observed signals of Ni-OH and Fe-OH bonds at 500–800 cm^−1^ in FTIR spectra of NiFe-CNG after the OER indicate that the adsorption process of OH^−^ occurs at both Ni and Fe sites. The increase of oxidation states over both Ni and Fe centers evident from the in situ XANES of NiFe-CNG manifests that the deprotonation process took place on both Ni and Fe sites. The above analyzed processes correspond well with the reaction coordinates proposed in Fig. 5d, e.

Given that the *OH deprotonation process could take place on both Ni and Fe sites, we simulated two energy barrier diagrams for NiFe-CNG during OER. The results demonstrate that the free energies of the RLS in both cases are 1.18 eV (deprotonation at the Fe center) and 1.38 eV (deprotonation at the Ni center), respectively, which are drastically lower than for the single-site pathway: Fe-CNG (2.37 eV), Ni-CNG (1.90 eV), Ni-Fe*-CNG (2.23 eV), and Ni*-Fe-CNG (3.23 eV) (Fig. 5d, e and Supplementary Figs. 63–66). The in situ XANES analysis of NiFe-CNG (Fig. 4a, b) shows that the oxidation state of Fe is more reversible than that of Ni. This may explain that the deprotonation step of OH is more favorable to occur at the Fe center in Ni-O-Fe moieties (Fig. 5d, e). Moreover, the adsorption energies over other intermediates in the dual-site mechanism were significantly optimized towards moderate values, i.e., neither too high nor too low compared with the single-site mechanism (Fig. 5a). The adsorption of reaction intermediates at Fe sites is much stronger than on Ni and Co sites; however, the hybrid compounds with both Fe and Ni or Co atoms usually display significantly improved OER performances^62^. In our case, almost all the intermediates chemisorbed at Fe sites through a single-site mechanism are more strongly bonded than that at Ni sites (Supplementary Figs. 68 and 69). This indicates that the Fe site is energetically more preferential for the adsorption of intermediates. To achieve a deeper understanding of the respective adsorption behavior of Ni and Fe sites, the spin density of the three catalysts was calculated, and the results show that Ni atoms adopt low spin configurations, while Fe atoms are high spin in all samples (Fig. 5b and Supplementary Fig. 70), which implies the presence of unpaired Fe electrons in *t*_2g_ and *e*_g_ orbitals. Based on the XPS measurements, the Ni species in the catalyst are diamagnetic. The diamagnetic property may be attributed to the square planar structure in SACs. The slight spin evident from calculated results indicates that the oxidation state of Ni species increased during OER, leading to the removal of electrons from *d* orbitals. It is worth noting that the increase in the oxidation state of Ni species is also evident from in situ XANES and CV measurements (Fig. 4a and Supplementary Fig. 45), which differ from the XANES and XPS data for fresh catalysts (Supplementary Figs. 31 and 36 and Fig. 2). The possible orbital interactions show that the Fe site has a higher bond order toward *OH, *O, *OOH, and O_2_ compared with Ni sites (Supplementary Figs. 71–73), which indicates that Fe sites are favorable for bonding to OER intermediates. However, based on the Sabatier principle, an ideal catalyst should bind the reaction intermediates neither too strongly nor too weakly^66^. Therefore, the high OER performance of NiFe-CNG should also be related to the Ni sites. Based on previous studies, the magnetic coupling of two metal atoms Ni-O-Fe species may lead to quantum spin-exchange interactions^67,68^, which established an electron transfer channel along the lattice and further facilitates charge transport inside the catalyst. Therefore, the newly formed Ni-O-Fe bonds in the catalysts may provide an electron channel for the OER. This is also evident from the calculated DOS (Fig. 5c), in which the catalysts containing paramagnetic Fe species (NiFe-CNG) show quite different DOS structures. Their spin-up and spin-down components around the Fermi level are strongly fluctuant after connecting with the bridging oxygen atoms, leading to remarkable electronic perturbation^69^. The above discussion implies that the synergistic effect of Ni and Fe through the presence of Fe-O-Ni bonds in NiFe-CNG is a key prerequisite for high OER activity^70,71^.

Through combining our experimental evidence with DFT calculations, we propose an overall reaction pathway for the NiFe-CNG catalyst to rationalize its enhanced OER activity as depicted in Fig. 5f. The OER starts with the interatomic electronic interplay of the newly constructed Ni-O-Fe active sites. Through benefitting from the unblocked spin channel in the Ni-O-Fe bond and from the optimization of the local electronic structure, the adsorption energies of the intermediates are greatly enhanced, leading to a dual-site reaction pathway with the participation of both Ni and Fe atoms.

**In summary**, we present new mechanistic insight into the synergisms of Ni and Fe sites during the OER with dual-site NiFe SACs. To start from high-performance model SACs, we successfully developed a novel and straightforward strategy based on g-C_3_N_4_ as the precursor for the construction of graphene-based single-site and dual-site SACs with high metal contents of up to 10 wt%. We studied the formation mechanism of the emerging SACs and identified the synergetic functionalities of g-C_3_N_4_, glucose, and metal ions during the growth process. This insight provides a universal strategy for the isolation of single atoms on the carbon support. The atomistic dispersions and local structures of the so obtained SACs were analyzed with a wide range of techniques, such as HAADF-STEM and XAS.

The as-prepared Fe-CNG SAC exhibits excellent electrocatalytic ORR activity with an extremely high half-wave potential of 0.89 V vs. RHE and stable long-term operation over 20,000 CV cycles. Interestingly, the OER performance of the as-prepared inactive single-site SACs (Ni/Fe-CNG) was significantly promoted through the introduction of both Ni and Fe atoms into the same graphitic matrix with an impressive OER performance of 270 mV and excellent stability over 72 h at the current density of 10 mA cm^−2^, which is superior to most of the current SACs for OER.

Most importantly, in situ XAS measurements of single-site Ni, Fe and dual-site NiFe SACs provide unprecedented experimental insight into the behavior of the Ni and Fe species in the hybrid NiFe SAC. Our monitoring data show for the first time that the dual Ni/Fe centers differ notably with respect to changes in their coordination environments and oxidation states during OER from the chemical transformations of their analog single site (Ni and Fe) SACs. The environment of the Ni sites preferably undergoes structural reconstruction processes, which promotes the formation of Ni-O-Fe moieties as true active species for the OER. DFT simulations further confirm that the OER pathway of NiFe-CNG follows a dual-site reaction mechanism via the newly formed Ni-O-Fe moieties. Both Ni and Fe sites participate in the *OH deprotonation process, leading to the formation of bridging O_2_ atop the Ni-O-Fe bonds during the OER. The different spin states of Ni and Fe centers in the Ni-O-Fe bonds create spin channels, which facilitate charge transport inside the catalyst and optimize the local electronic structure along with the adsorption behavior of the OER intermediates.

All in all, we provide combined and unique experimental and theoretical insights into the OER mechanisms of NiFe dual-site SACs. This was furthermore enabled through our versatile, low-cost, and convenient strategy to access complementary single and dual-site SACs with high activity. Such comprehensive insight is essential and impactful for optimizing dual-site SACs mechanisms towards OER applications.

## Supplementary information

Supplementary Information

## Data Availability

The data that support the findings of this study are available from the corresponding author upon request.
