# Supplementary material for: Mechanistic insight into the active centers of single/dual-atom Ni/Fe-based oxygen electrocatalysts
Source: Nat Commun. 2021 Sep 22;12:5589. doi: 10.1038/s41467-021-25811-0 (PMC8458471; doi:10.1038/s41467-021-25811-0)
Supplement: Supplementary file 1 — Supplementary Information [file 41467_2021_25811_MOESM1_ESM.pdf]

Supporting Information

Mechanistic insight into the active centers of single/dual-atom Ni/Fe-based oxygen electrocatalysts

Table of Contents

Methods 6

Materials. 6

Synthesis of the catalysts. 6

Electrochemical Measurements 6

Density functional theory (DFT) calculations. 9

Figure S1. Raman spectrum and PXRD (MoK_α radiation) of bulk g-C_3N_4. 12

Figure S2. Raman spectra of bulk NiFe-CNG, Fe-CNG, Co-CNG and Ni-CNG SACs.................. 12

Figure S3. PXRD patterns of NiFe-CNG, Fe-CNG, Co-CNG and Ni-CNG SACs. 13

Figure S4. TEM images of bulk g-C_3N_4. 13

Figure S5. TEM images of NiFe-CNG 14

Figure S6. TEM images of Fe-CNG 14

Figure S7. TEM images of Co-CNG 15

Figure S8. TEM images of Ni-CNG 15

Figure S9. HAADF-STEM images of Ni-CNG 16

Figure S10. HAADF-STEM images and of Co-CNG 16

Figure S11. HAADF-STEM images of Fe-CNG 17

Figure S12. SEM images and EDS elemental mappings of NiFe-CNG. 17

Figure S13. SEM images and EDS elemental mappings of Ni-CNG 18

Figure S14. SEM images and EDS elemental mappings of Co-CNG.. 18

Figure S15. SEM images and EDS elemental mappings of Fe-CNG. 19

Figure S16. HAADF-STEM (left) and BF-STEM (right) images of NiFe-CNG 19

Figure S17. HAADF-STEM (left) and BF-STEM (right) images of NiFe-CNG 20

Figure S18. TEM image of Ni-CN. 20

Figure S19. TEM image of Co-CN. 21

Figure S20. TEM image of Fe-CN. 21

Figure S21. PXRD patterns of Ni-CN, Co-CN, and Fe-CN 22

Figure S22. Raman spectra of Ni-CN, Co-CN, and Fe-CN 22

Figure S23. TEM image and PXRD pattern of the sample using nickel acetylacetonate 23

Figure S24. TEM image and PXRD pattern of the sample using nickel acetylacetonate, glucose and g-C_3N_4 as the precursor. 23

Figure S25. PXRD patterns of glucose-Ni complex, $Ni(NO_3)_2$, and glucose. 24

Figure S26. TEM image of Glucose-CN 25

Figure S27. Raman spectrum of Glucose-CN 25

Figure S28. Representative photos of the samples before and after pyrolysis at 900 °C 26

Figure S29. TEM images of NiFe-CNG-1000 26

Figure S30. XPS survey spectra of CNG, Ni-CNG, Co-CNG, Fe-CNG, and NiFe-CNG. 27

Figure S31. High-resolution XPS 2p and 3p spectra 28

Figure S32. High-resolution XPS spectra 28

Figure S33. Experimental and calculated FT-EXAFS spectra of Ni-CNG. 29

Figure S34. Experimental and calculated FT-EXAFS spectra of Co-CNG. 30

Figure S35. Experimental and calculated FT-EXAFS spectra of Fe-CNG. 31

Figure S36. Ni and Fe K-edge XANES spectra of NiFe-CNG. 32

Figure S37. Fitting of Ni and Fe K-edge FT-EXAFS spectra of NiFe-CNG. 32

Figure S38. WT-EXAFS spectra of NiFe-CNG 33

Figure S39. Cyclic voltammetry curves 33

Figure S40. Electrocatalytic performance of CNG, Fe-CN, Co-CN and Ni-CN 34

Figure S41. HAADF-STEM images of CoFe-CNG 34

Figure S42. LSV curves of Fe-CNG 35

Figure S43. XANES and FT-EXAFS spectra of Fe-CNG 35

Figure S44. Zn-air battery measurements: 36

Figure S45. OER polarization curves of Ni-CNG and Fe-CNG 37

Figure S46. Cyclic voltammetry curves of NiFe-CNG, Ni-CNG and Fe-CNG 37

Figure S47. TEM images of NiFe-CNG 38

Figure S48. Electrochemical impedance spectra and Bode plots 39

Figure S49. FTIR spectra of the dry samples 40

Figure S50. Wavelet transforms for the k^3-weighted EXAFS signals of NiFe-CNG. 41

Figure S51. Wavelet transforms for the k^3-weighted EXAFS signals of NiFe-CNG. 41

Figure S52. Wavelet transforms for the k^3-weighted EXAFS signals of NiFe-CNG. 42

Figure S53. Wavelet transforms for the k^3-weighted EXAFS signals of NiFe-CNG 42

Figure S54. Fitting of the experimental Ni K-edge EXAFS spectra of NiFe-CNG 43

Figure S55. Fitting of the experimental Fe K-edge EXAFS spectra of NiFe-CNG. 44

Figure S56. In situ Ni K-edge XANES spectra of Ni-CNG 44

Figure S57. In situ Fe-K edge XANES spectra of Fe-CNG 45

Figure S58. FT-EXAFS spectra and corresponding WT-EXAFS spectra of Ni-CNG 45

Figure S59. Fitting of the experimental Ni K-edge EXAFS spectra of Ni-CNG. 46

Figure S60. FT-EXAFS spectra and corresponding WT-EXAFS spectra of Fe-CNG 47

Figure S61. Fitting of the experimental Fe K-edge EXAFS spectra of Fe-CNG 48

Figure S62. DFT models for the NiFe-CNG, Ni-CNG, and Fe-CNG catalysts. 48

Figure S63. Free energy diagram of OER cycling at the Fe site of the Fe-CNG model. 49

Figure S64. Free energy diagram of OER cycling at the Ni site of the Ni-CNG model. 49

Figure S65. Free energy diagram of OER cycling at the Fe site of the NiFe-CNG model. 50

Figure S66. Free energy diagram of OER cycling at the Ni site on the NiFe-CNG model. 50

Figure S67. Free energy comparison of bridging O-O and *O on the Ni-O-Fe site 51

Figure S68. Free energy comparison of *OH, *O, *OOH adsorption, and *OOH deprotonation... 51

Figure S69. Free energy comparison of *OH, *O, *OOH adsorption and *OOH deprotonation.... 52

Figure S70. Spin density pattern and spin channels of the Ni and Fe sites 52

Figure S71. Diagram of of d-electron configurations 53

Figure S72. Diagram of the orbital interactions 53

Figure S73. Diagram of the orbital interactions 54

Table S1. Entropic contributions to free energies. 54

Table S2. Zero-point energy corrections to free energies (single site). 55

Table S3. Zero-point energy corrections to free energies (dual sites). 55

Table S4. Free energies of *OH, *O and *OOH 55

Table S5. Free energies of *OH, *O and *O-O 56

Table S6. Mass and atomic content of the metallic elements in Ni-CNG, Co-CNG, FeCNG, and NiFe-CNG. 56

Table S7. ICP-MS measurements of the electrolytes before and after the long-term OER tests. 56

Table S8. Main interatomic distances, atomic coordination numbers (*N*) and Debye-Waller factors (σ^2) of Ni-CNG, Co-CNG, and Fe-CNG 57

Table S9. Main interatomic distances, atomic coordination numbers (*N*) and Debye-Waller factors (σ^2) of NiFe-CNG. 58

Table S10. Comparison of ORR performance and stability 59

Table S11. Comparison of OER performance and stability 60

Table S12. Comparison of Zn-air battery performance of the as-prepared catalysts with recent representative SACs. 61

Supplementary Note 1 62

Figure S74. Ni LMN Auger signal of Ni-CNG (red) and Fe 2p signal (black) of NiFe-CNG......... 63

Figure S75. Ni and Fe 3p signals of NiFe-CNG compared with Ni-CNG and Fe-CNG. 64

Supplementary Note 2 64

Figure S76. Ni K-edge k^3-weighted EXAFS of pristine NiFe-CNG, NiFe-CNG at 1.5 V vs. RHE vs. $Ni(OH)_2$. 64

Figure S77. TEM images of NiFe-CNG after OER 65

Figure S78. TEM images of Ni-CNG after 1000 CV cycles at the potential range of 0-1.7 V vs. RHE.. 65

Figure S79. HAADF-STEM measurement of NiFe-CNG after OER 66

Figure S80. Raman spectra of post-catalytic NiFe-NG, Ni-CNG and commercial $Ni(OH)_2$ 66

Figure S81. High-resolution XP spectra of CNG and post-catalytic NiFe-CNG. 67

Figure S82. Representative HAADF-STEM images of a fresh NiFe-CNG sample. 68

References 69

Methods

Materials.

Dicyandiamide ($C_2H_4N_4$) (>99 %), $Ni(NO_3)_2·6H_2O$ (≥98.0 %), $Co(NO_3)_2·6H_2O$ (≥98.0 %), $Fe(NO_3)_2·9H_2O$ (≥98.0 %)), and α-D-glucose ($C_6H_{12}O_6$) (MW=180.16, 96.0 %) were purchased from Sigma-Aldrich. Ar gas used in the synthesis was purchased from PanGas AG, Switzerland, ⩾99.999%.

Synthesis of the catalysts.

g-C_3N_4. g-C_3N_4 was obtained from a thermopolymerization procedure, in which $C_2H_4N_4$ was placed into a crucible and heated at 550 °C for 2 h under air atmosphere with a ramp rate of about 2.3 °C/min.

SACs. The obtained yellow bulk g-C_3N_4 was then ground into a fine powder with a mortar for further use. 1000 mg of the above prepared g-C_3N_4, 120 mg $C_6H_{12}O_6$ and certain amounts of metal salts (0.06 mmol, corresponding to 18 mg $Ni(NO_3)_2·6H_2O$, 18 mg $Co(NO_3)_2·6H_2O$ and 24 mg $Fe(NO_3)_2·9H_2O$ were mixed into 40 ml in deionized water. After 3 h of ultrasonication, followed by 24 h stirring treatment, the mixture was further subjected to an evaporation procedure to remove the water and to achieve a dried mixture. The above solid was further ground into a fine powder and subsequently treated with a pyrolysis procedure in a tube furnace at temperatures 900 °C under protective Ar atmosphere. Different ratios of the Ni/Fe in the dual-site NiFe-CNG catalysts were obtained by adjusting the molar ratio of Ni and Fe ions, while keeping the total molar mass of the metal salts at 0.06 mmol, and the mass of g-C_3N_4 and glucose at 1000 mg and 120 mg, respectively. Fe SACs with different metal contents were prepared via the same procedure while adding different amounts of $Fe(NO_3)_2·9H_2O$.

CNG. The reference sample (CNG) was fabricated with the same procedure, except for the absence of metal salts.

Ni/Co/Fe-CN. Metal alloys encapsulated into CNTs and graphene shells were achieved with the same protocol except for adding glucose.

Electrochemical Measurements

ORR. The electrochemical oxygen reduction measurements were conducted in 0.1 M KOH electrolyte at room temperature with a standard three-electrode system connected with the Metrohm Autolab PGSTAT302N potentiostat. A graphite rod was used as counter electrode and a Ag/AgCl electrode (3 M KCl) was employed as reference electrode, and the working electrodes were the RDE and a RRDE (GC disc with r = 2.5 mm and 375 µm gap, Pt ring with width = 375 µm, r_{inner} = 2.875 mm, and r_{outer} = 3.250 mm). To prepare the working electrode, 3.0 mg of catalysts were dispersed in 600 µL ethanol and 20 µL of 5 wt % Nafion solution and sonicated for 30 min to form a homogeneous ink. Then, 3.00 µL of homogeneous ink was dropped onto the working electrode to achieve a 0.200 mg cm^{-2} loading amount. Before each ORR measurement, the 0.1 M KOH electrolyte was bubbled with high purity of O_2 for 30 min and maintained over the electrolyte surface during the ORR test to sustain the O_2 saturation. Prior to measurements, the working electrode was subjected to at least 10 CV cycles in the potential range of 0.2 V-1.2 V *vs.* RHE at 100 mV s^{-1} to reach a stable state. LSV was carried out at

a rotation speed of 1600 rpm with a scan rate of 5.0 mV s^{-1} for the acquisition of polarization curves. The RRDE measurement was performed at a constant ring potential of 1.3 V *vs.* RHE.

The number of electrons transferred and the H_2O_2 yield were evaluated from RRDE measurements using the following equations:

Number of electron transfer (n) during ORR:

$$n=4\times\frac{I_d}{\left(I_d+\frac{I_r}{N}\right)} \quad (1)$$

Percentage of peroxide (% HO_2^-) during the ORR tests:

$$\%\ HO_2^-=200\times\frac{I_r}{(N\times I_d+I_r)} \quad (2)$$

where I_d and I_r are disk and ring current densities, respectively, and N is the current collection efficiency of the Pt ring which is 0.25.

The electron transfer number and kinetics of ORR from RDE measurements were evaluated using the following Koutecky–Levich equation:

$$\frac{1}{j}=\frac{1}{j_l}+\frac{1}{j_k}=\frac{1}{B\omega^{0.5}}+\frac{1}{j_k} \quad (3)$$

$$B=0.64nFAC_0D_0^{2/3}V^{-1/6} \quad (4)$$

$$j_k=nFkC_0 \quad (5)$$

where j is the measured current density, j_l and j_k are the limiting and kinetic current densities, B is the slope of K-L plots, ω is the rotation rate of the disk electrode, n is the number of transferred electrons in ORR, F is the Faraday constant (96485 C mol^{-1}), A is the geometric area of the electrode (A = 0.07065 cm^2), C_0 is the concentration of O_2 which is 1.2×10^{-6} mol cm^{-3} in 0.1 M KOH, D_0 is the O_2 diffusion coefficient (1.9×10^{-5} cm^2 s^{-1}), V is the kinematic viscosity of the solution (0.01 cm^2 s^{-1}), and k is the electron transfer rate constant.

OER. Electrochemical water oxidation measurements were carried out on an Autolab PGSTAT204 electrochemistry workstation with the standard three-electrode system in 1 M KOH electrolyte. Glassy carbon (GC) electrode loaded with catalyst was used as working electrode. 2.5 mg of catalyst were dispersed in 0.5 mL water and 0.5 mL ethanol. After 30 min of ultrasonication, 50 µL of 5 wt % Nafion solution was further added into the above solution with another 60 min of sonication to form a homogeneous ink. Then 5 µL of the catalyst ink (containing 12.5 µg of catalyst) was loaded onto a glassy-carbon electrode (2 mm in diameter). Ag/AgCl with saturated KCl filling solution and graphite rod were used as reference electrode and counter electrode, respectively. All catalyst electrodes were

continuously scanned for 10 times CV before measuring polarization curves. The scan rates for all the LSV and CV measurements were set to 10 mV s^{-1} to minimize the capacitive current.

Zn-air battery. Zn-air battery tests were performed at room temperature in an aqueous solution of 6.0 M KOH and 0.2 M zinc acetate dihydrate. A standard two-electrode system was used (Metrohm Autolab PGSTAT302N potentiostat), with a polished Zn rod as the anode and reference electrode, and the as-prepared materials as the cathode, respectively. The cathode was prepared by dropping ink of the catalysts on a pieces of carbon paper (0.5×1 cm^{-2}) with a loading mass of 2.0 mg cm^{-2}. As a reference, commercial 20 wt.% Pt/RuO_2 (mass ratio 1:1) was prepared as cathode following the same procedure. The galvanostatic discharge test was conducted at a current density of 10 mA/cm^2.

Materials characterization. Powder X-ray diffraction (PXRD) patterns were recorded on a STOE STADI P diffractometer (transmission mode, Ge monochromator) with Mo K_α radiation. Raman spectra were measured with a Renishaw Ramascope as pristine powder samples on quartz glass slides. Inductively coupled plasma mass spectrometry (ICP-MS) measurements were carried out on an Agilent 8800 instrument. All samples were first annealed under air atmosphere and then dissolved in aqua regia. Annular dark-field scanning transmission electron microscopy (ADF-STEM) was performed on aberration-corrected JEOL ARM300CF STEM equipped with a JEOL ETA corrector operated at an accelerating voltage of 80 kV located in the electron Physical Sciences Imaging Centre (ePSIC) at Diamond Light Source.

XPS analysis was carried out with a Quantum 2000 X-ray photoelectron spectrometer (Physical Electronics, Minnesota, United States), equipped with an Al K_α monochromatic source (1486.6 eV), a hemispherical capacitor electron-energy analyzer, and a 16-channel plate detector. All the spectra were acquired with an emission angle of 45° in fixed analyzer transmission mode using a nominal X-ray beam-spot size of 150 μm or 200 μm. Survey and high-resolution spectra were acquired at a pass energy of 117.40 eV (energy step of 0.5 eV) and 23.5 eV (energy step of 0.05 eV), respectively. Samples were prepared by pressing the material between thin indium foils, to embed the powder in the soft metal, and to avoid contact with other surfaces once separating the two foils to expose the surface of interest. The atomic fractions of the detectable elements were estimated by using the formula:[1]

$$x_{\mathrm{a}}=\frac{I_{\mathrm{a}}/RSF_{\mathrm{a}}}{\sum_{\mathrm{i}}^{\mathrm{n}} I_i/RSF_{\mathrm{i}}} \tag{6}$$

The relative sensitivity factors (RSF_{i}) were taken from the analysis software of the XP-spectrometer. A Shirley background was subtracted from the area of the peaks to estimate their intensity (I_{i}). The formula provides estimates for the atomic fraction of the elements within the volume probed by the technique, under the assumption that the analyzed samples are homogenous in depth. Deviations occur if the assumption is not valid. In addition, hydrogen is not included in the quantitative analysis, as the element

is not detectable by XPS. In this work, the main component of the C 1*s* signal was used as an internal reference, which was centered at 284.7 eV.[2]

X-ray absorption spectroscopy. The ex situ and in situ XANES and EXAFS experiments were carried out at beamline 4-3 at SLAC National Accelerator Laboratory (California, United States) in fluorescence mode using the Lytle detector. The X-ray beam was collimated with a Si-coated mirror, and the energy was scanned with a channel-cut Si [111] monochromator. All the ex situ measurements were carried out on solid powder samples dispersed on carbon paper. The in situ measurements were conducted by using an in-house designed electrochemical cell filled with 1 M KOH electrolyte. A platinum mesh and Ag/AgCl in saturated KCl electrode were used as counter and reference electrode, respectively. The working electrodes were fabricated by loading the catalyst ink on carbon paper using the same routine described in the electrochemical measurements section. The post-catalytic XAS spectra after OER were measured after 20000 CV cycles scanned at the potential range of 0−1.8 V vs RHE. The measured EXAFS spectra were obtained by standard data reduction, absorption edge energy calibration and background subtraction as implemented in ATHENA.[3] The spectra were then reduced into the range $\Delta k \approx 3\text{-}9$ Å^{-1} and Fourier-Transform to $|FT(k^3\chi(k))|$ into the real-space interval $\Delta R \approx 0\text{-}6$ Å. To obtain the main values for interatomic distances, coordination numbers (N), and Debye-Waller factors (σ^2), nonlinear least-squares fitting of the $FT|(k^3\chi(k))|$ spectra was carried out with ARTEMIS. Amplitude and phase shift for single and multiple scattering paths were computed using FEFF6 code.[4] The amplitude reduction factor S_0^2 was calculated from fitting the $|FT(k^3\chi(k))|$ spectra of the respective standard Ni, Co or Fe metal foils and kept constant for fitting all the $|FT(k^3\chi(k))|$ spectra. All $|FT(k^3\chi(k))|$ spectra were fitted without phase corrections using the Hanning-window function into the range k_{min}-k_{max}=3-9 Å^{-1}; R_{min}-R_{max}=1.0-3.2 Å. All the fitting models are included in the supporting materials. WT-EXAFS spectra were calculated with MATLAB 2020 using the Matlab script written by Manuel Muñoz & François Farges.[5–7]

Density functional theory (DFT) calculations.

All spin-polarized density functional theory (DFT) calculations were completed using the projector augmented-wave (PAW) pesudopotentials as implemented in the Vienna Ab-initio Simulation Package (VASP)[8,9] with a cut-off energy of 450 eV. The generalized gradient approximation of Perdew-Burke-Ernzerhof functional (GGA/PBE) was used to describe the exchange and correlation potential.[10] The convergence criteria of energy and force were 1×10^{-5} eV and 0.05 eV/Å, respectively. The Brillouin-zone was sampled with 5×5×1 and 7×7×1 K-point grids for the structure optimizations and electron property calculations, respectively. Grimme dispersion corrected method was applied to properly describe the van der Waals interactions in the systems.[11] The periodical monolayer slabs based on graphene (001) 3×3 units were separated by a vacuum zone of 15 Å.

To simulate the OER pathway and to determine the reaction energy barrier, the computational hydrogen electrode (CHE) model described by Nørskov et al. was applied.[12,13] In this CHE model, the chemical potential of the proton-electron pair (i.e. the free energy per H) is determined simply by the chemical potential of gas-phase H_2 as shown in equation (7), which can be adjusted as a function of the applied potential:

$$\mu_{(\mathrm{H}^+)}+\mu_{(\mathrm{e}^-)}=1/2\,\mu_{\mathrm{H_2(g)}}-eU \tag{7}$$

The Gibbs free energy is calculated using

$$\Delta G=E_{\mathrm{DFT}}+\Delta ZPE-T\Delta S \tag{8}$$

where G is the Gibbs free energy, E_{DFT} is the electronic energy from DFT calculations, ZPE is zero-point energy, T is temperature of 298 K, and ΔS is the change in entropy. They were calculated from DFT modeling of vibrational frequencies and using standard tables for gas phase molecules. The potential dependence of reaction free energies in elementary steps involving proton−electron transfers was evaluated using the computational hydrogen electrode approach.

The conventional single-site reaction pathway was calculated from the following equations:

$$* + \mathrm{OH}^- \rightarrow *\mathrm{OH} + e^- \tag{9}$$

$$*\mathrm{OH} + \mathrm{OH}^- \rightarrow *\mathrm{O} + \mathrm{H_2O}(l) + e^- \tag{10}$$

$$*\mathrm{O} + \mathrm{OH}^- \rightarrow *\mathrm{OOH} + e^- \tag{11}$$

$$*\mathrm{OOH}+ \mathrm{OH}^- \rightarrow * + \mathrm{O_2}(g) + \mathrm{H_2O}(l)+ e^- \tag{12}$$

The Gibbs free energy changes for each step can be calculated through equations (13-15):

$$\Delta G_1 = \Delta G_{*\mathrm{OH}} - eU + \Delta G_{\mathrm{pH}} \tag{13}$$

$$\Delta G_2 = \Delta G_{*\mathrm{O}} - \Delta G_{*\mathrm{OH}} - eU + \Delta G_{\mathrm{pH}} \tag{14}$$

$$\Delta G_3 = \Delta G_{*\mathrm{OOH}} - \Delta G_{*\mathrm{O}} - eU + \Delta G_{\mathrm{pH}} \tag{15}$$

$$\Delta G_4 = 4.92\ \mathrm{eV} - \Delta G_{*\mathrm{OOH}} - eU + \Delta G_{\mathrm{pH}} \tag{16}$$

The Gibbs free energy differences of various intermediates were determined by equations (17-19):

$$\Delta E_{*\mathrm{OH}} = E_{*\mathrm{OH}} - E_{*} - \left(\mu_{\mathrm{H_2O}(l)} - 1/2\mu_{\mathrm{H_2}(g)}\right) \tag{17}$$

$$\Delta E_{*\mathrm{O}} = E_{*\mathrm{O}} - E_{*} - \left(\mu_{\mathrm{H_2O}(l)} - \mu_{\mathrm{H_2}(g)}\right) \tag{18}$$

$$\Delta E_{*\mathrm{OOH}} = E_{*\mathrm{OOH}} - E_{*} - \left(2\mu_{\mathrm{H_2O}(l)} - 3/2\mu_{\mathrm{H_2}(g)}\right) \tag{19}$$

For the dual-site reaction pathway, Gibbs free energy differences of various intermediates can be determined by following equations:[14]

$$\Delta E_{(\mathrm{Fe}^* + \mathrm{Ni}^*\text{-OH})} = E_{(\mathrm{Fe}^* + \mathrm{Ni}^*\text{-OH})} - E_{(\mathrm{Fe}^* + \mathrm{Ni}^*)} - \left(\mu_{\mathrm{H_2O}(l)} - 1/2\mu_{\mathrm{H_2}(g)}\right) \tag{20}$$

$$\Delta E_{(\mathrm{Fe}^*\text{-OH} + \mathrm{Ni}^*)} = E_{(\mathrm{Fe}^*\text{-OH} + \mathrm{Ni}^*)} - E_{(\mathrm{Fe}^* + \mathrm{Ni}^*)} - \left(\mu_{\mathrm{H_2O}\,(l)} - 1/2\mu_{\mathrm{H_2}(g)}\right) \tag{21}$$

$$\Delta E_{(\mathrm{Fe}^*\text{-OH} + \mathrm{Ni}^*\text{-OH})} = E_{(\mathrm{Fe}^*\text{-OH} + \mathrm{Ni}^*\text{-OH})} - \mathrm{E}_{(\mathrm{Fe}^* + \mathrm{Ni}^*)} - \left(2\mu_{\mathrm{H_2O}(l)} - \mu_{\mathrm{H_2}(g)}\right) \tag{22}$$

$$\Delta E_{(\mathrm{Fe}^*\text{-O} + \mathrm{Ni}^*\text{-OH})} = E_{(\mathrm{Fe}^*\text{-O} + \mathrm{Ni}^*\text{-OH})} - E_{(\mathrm{Fe}^* + \mathrm{Ni}^*)} - \left(2\mu_{\mathrm{H_2O}(l)} - 3/2\mu_{\mathrm{H_2}(g)}\right) \tag{23}$$

$$\Delta E_{(\mathrm{Fe}^*\text{-OH} + \mathrm{Ni}^*\text{-O})} = E_{(\mathrm{Fe}^*\text{-OH} + \mathrm{Ni}^*\text{-O})} - E_{(\mathrm{Fe}^* + \mathrm{Ni}^*)} - \left(2\mu_{\mathrm{H_2O}(l)} - 3/2\mu_{\mathrm{H_2}(g)}\right) \tag{24}$$

$$\Delta E_{(\mathrm{Fe}^*\text{-O} + \mathrm{Ni}^*\text{-O})} = E_{(\mathrm{Fe}^*\text{-O} + \mathrm{Ni}^*\text{-O})} - E_{(\mathrm{Fe}^* + \mathrm{Ni}^*)} - \left(2\mu_{\mathrm{H_2O}(l)} - 2\mu_{\mathrm{H_2}(g)}\right) \tag{25}$$

$$\Delta E_{(\mathrm{Fe}^*\text{-O-O-}^*\mathrm{Ni})} = E_{(\mathrm{Fe}^*\text{-O-O-}^*\mathrm{Ni})} - E_{(\mathrm{Fe}^*+\mathrm{Ni}^*)} - \left(2\mu_{\mathrm{H_2O}(l)} - 2\mu_{\mathrm{H_2}(g)}\right) \tag{26}$$

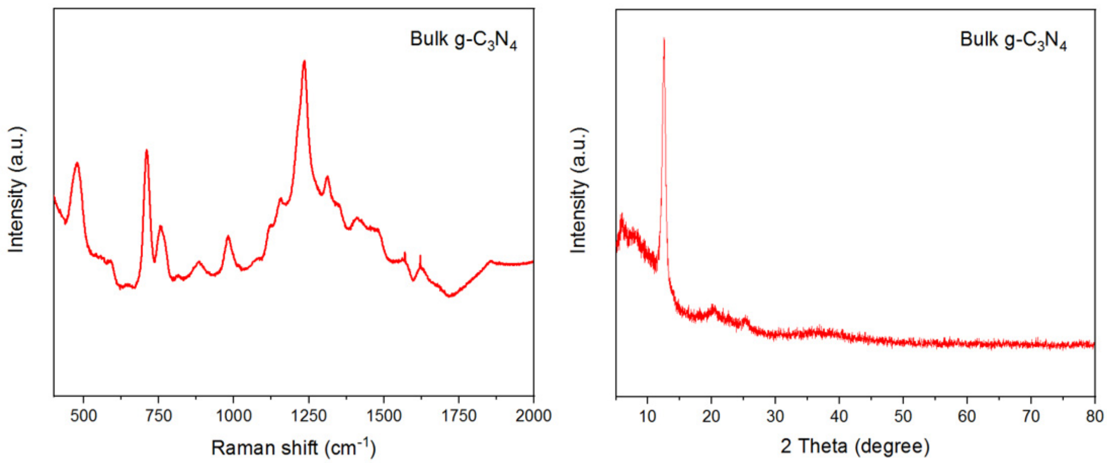

Figure S1. Raman spectrum and PXRD (MoK_α radiation) of bulk g-C_3N_4.

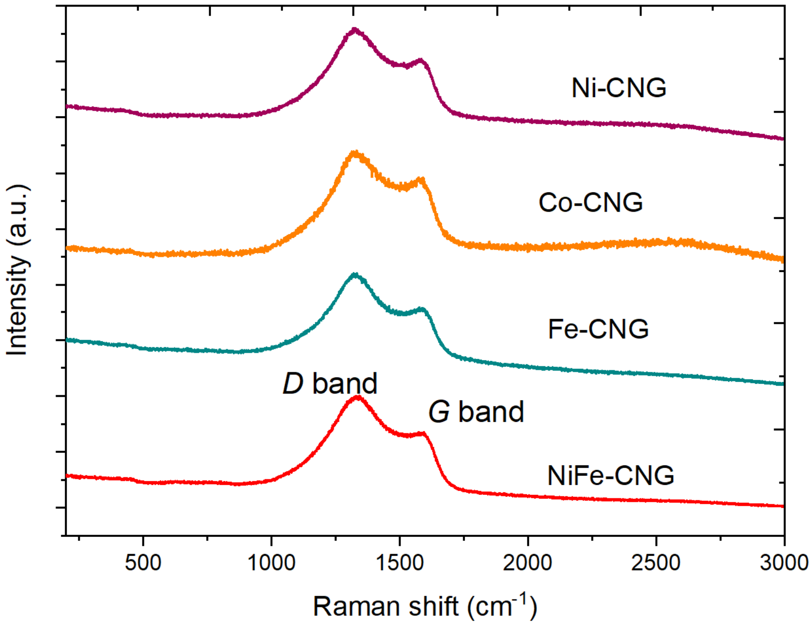

Figure S2. Raman spectra of bulk NiFe-CNG, Fe-CNG, Co-CNG and Ni-CNG SACs.

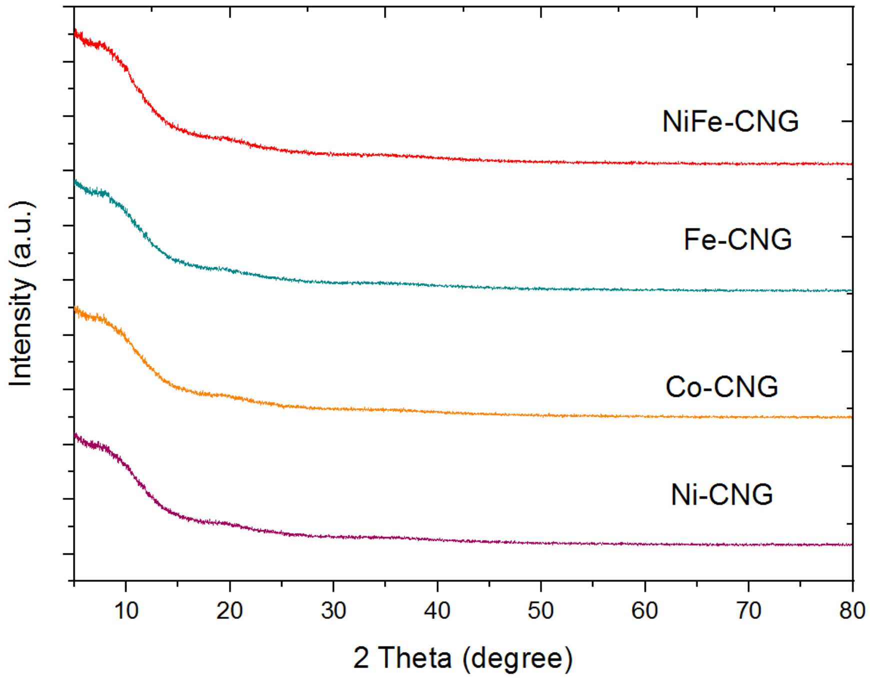

Figure S3. PXRD patterns of NiFe-CNG, Fe-CNG, Co-CNG and Ni-CNG SACs.

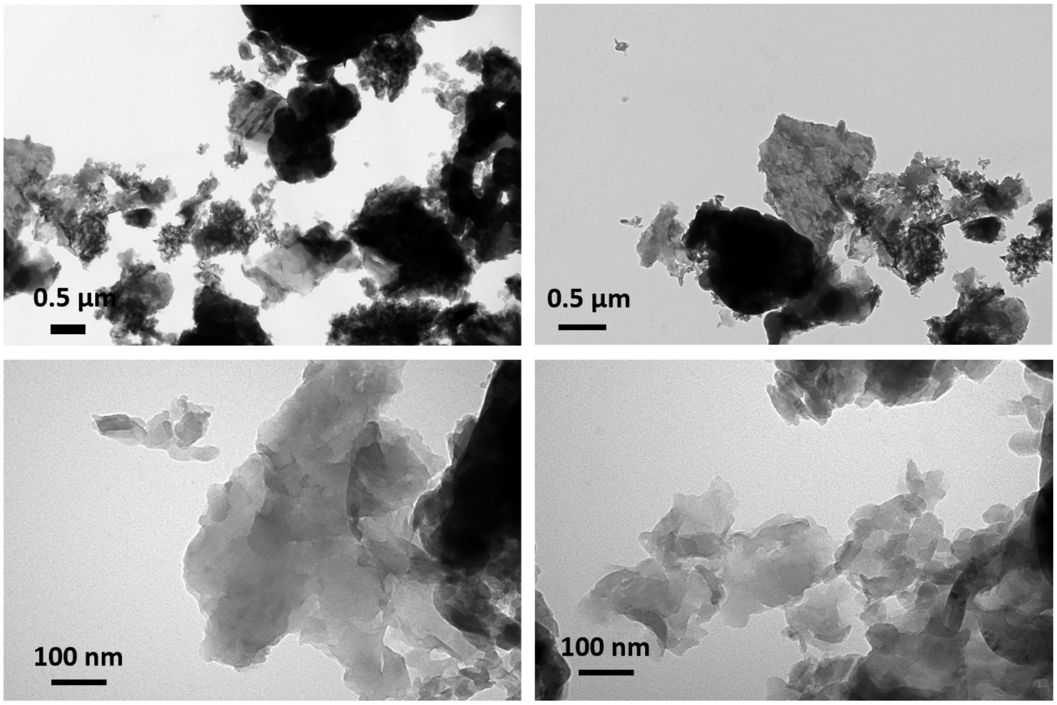

Figure S4. TEM images of bulk g-C_3N_4.

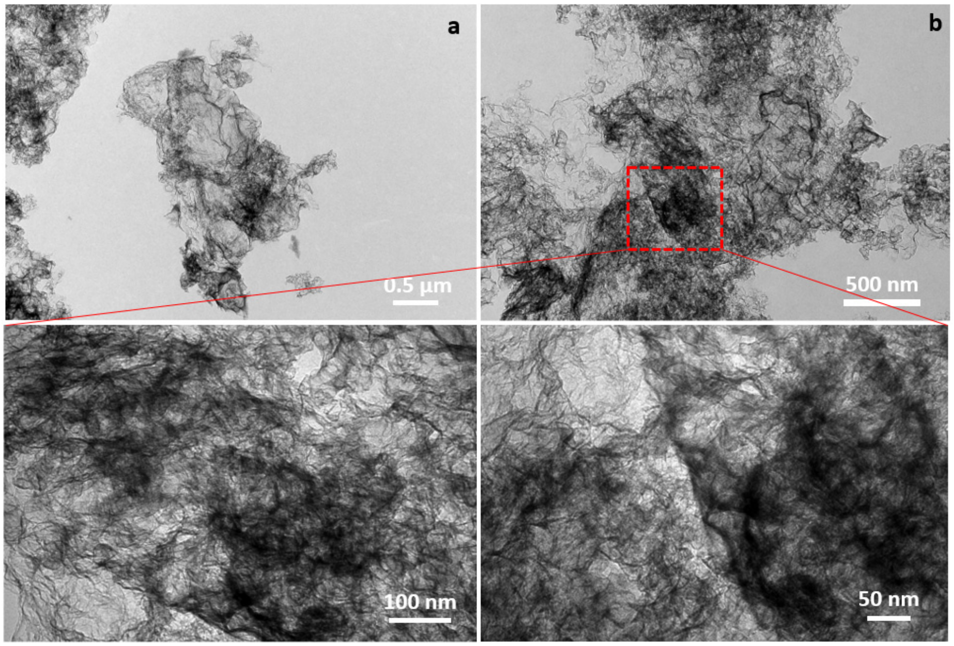

Figure S5. TEM images of NiFe-CNG at different magnifications. The dark positions are the overlapping graphene layers.

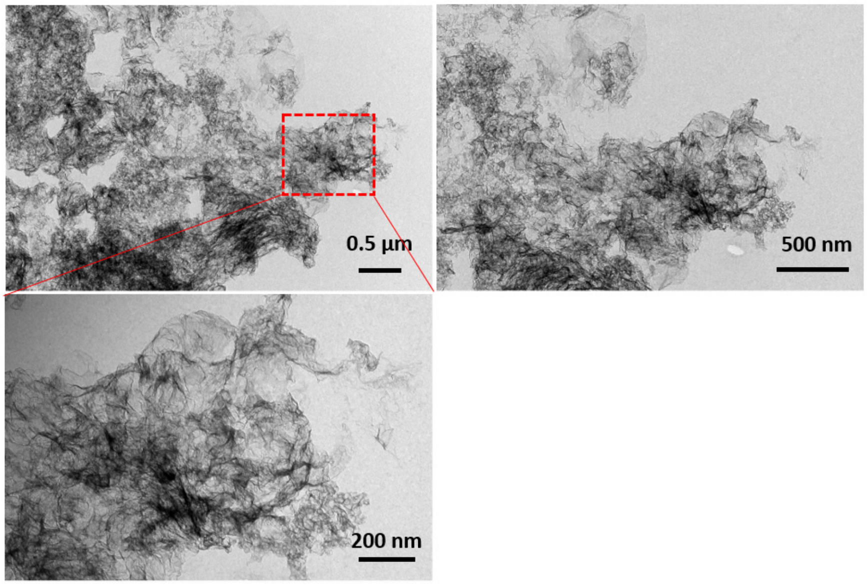

Figure S6. TEM images of Fe-CNG at different magnifications.

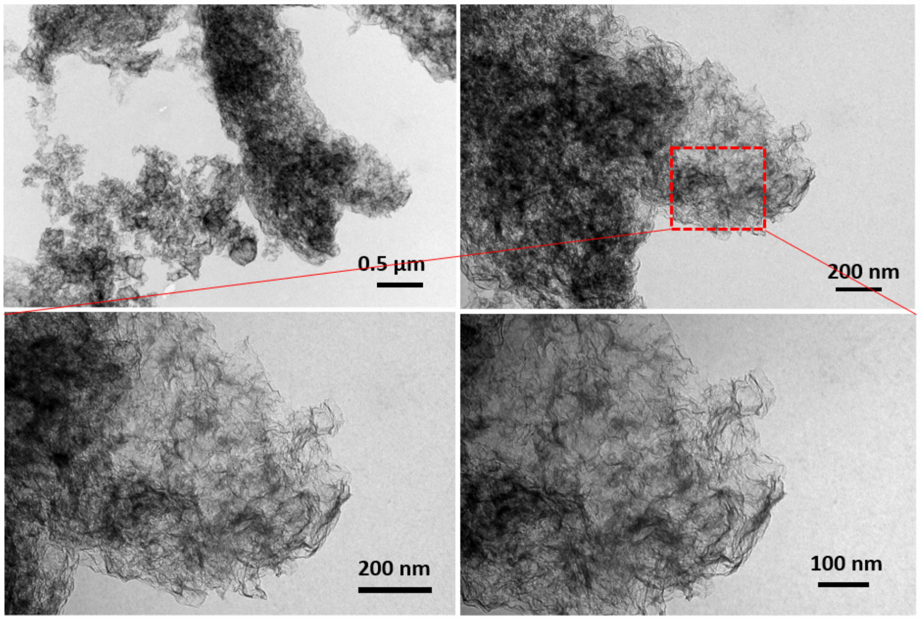

Figure S7. TEM images of Co-CNG at different magnifications.

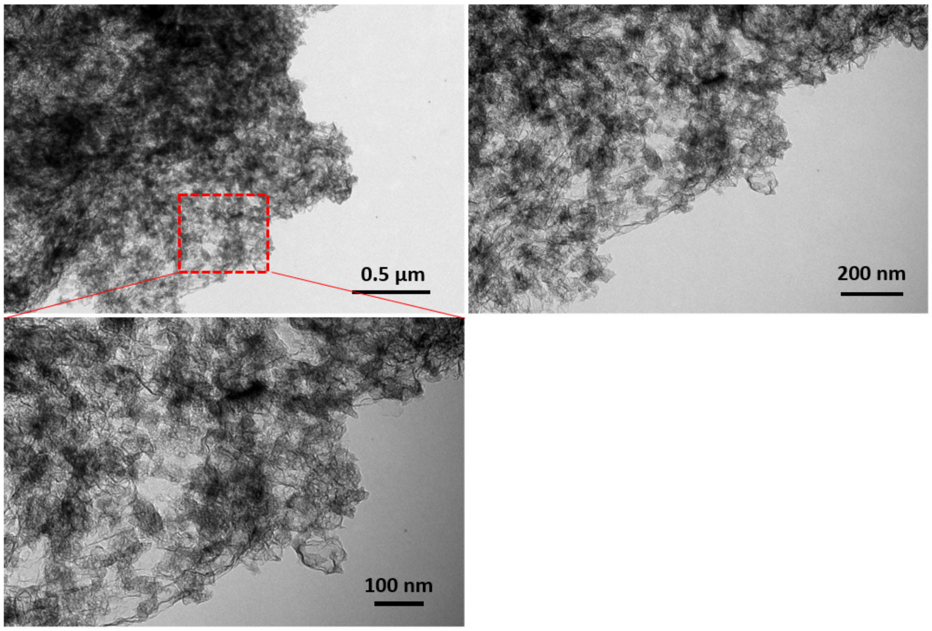

Figure S8. TEM images of Ni-CNG at different magnifications.

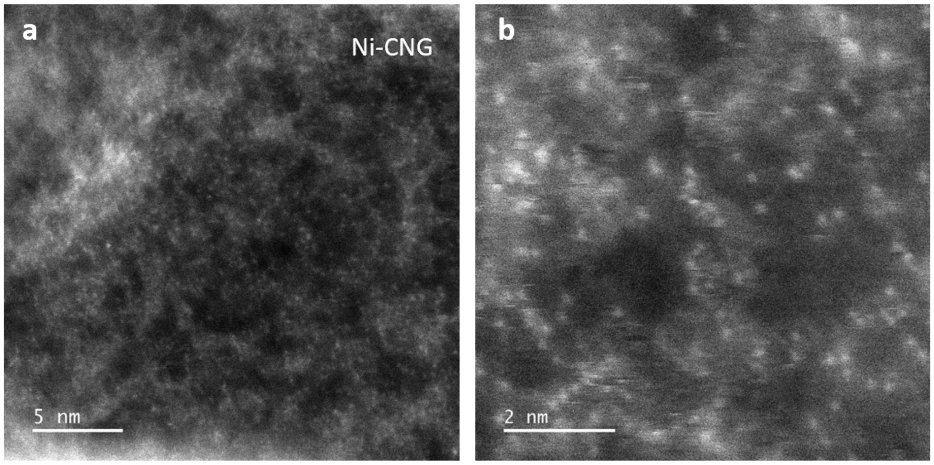

Figure S9. HAADF-STEM images of Ni-CNG at different magnifications.

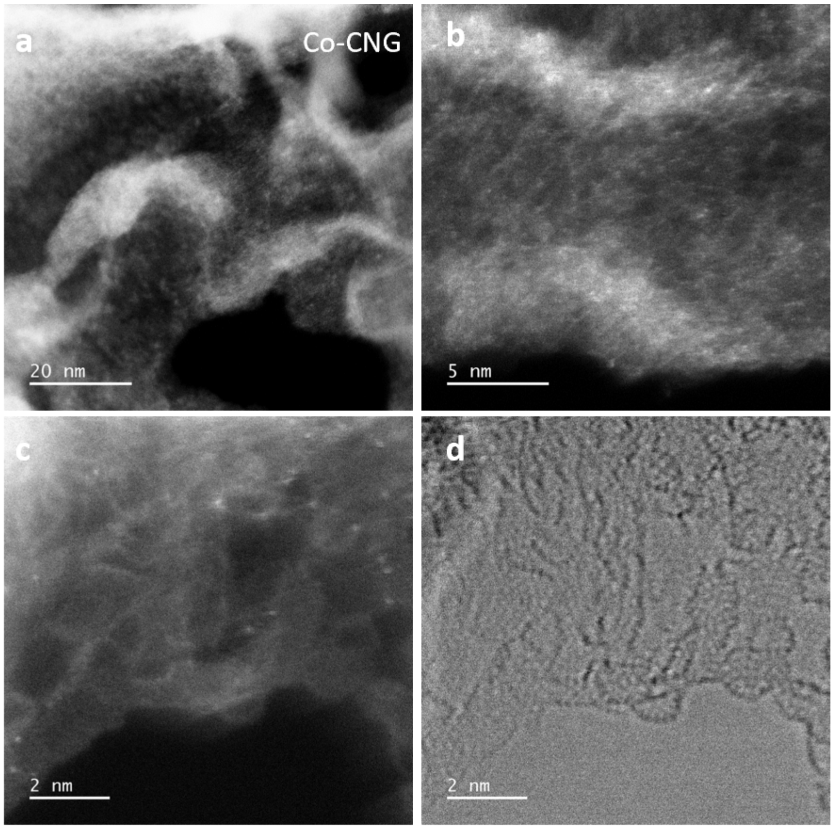

Figure S10. (a-c) HAADF-STEM images and of Co-CNG at different magnifications, where the bright dots are the Co atoms. (d) Corresponding BF-STEM image of (c), which shows the graphitic structure of the support material, the dark dots are the Co atoms.

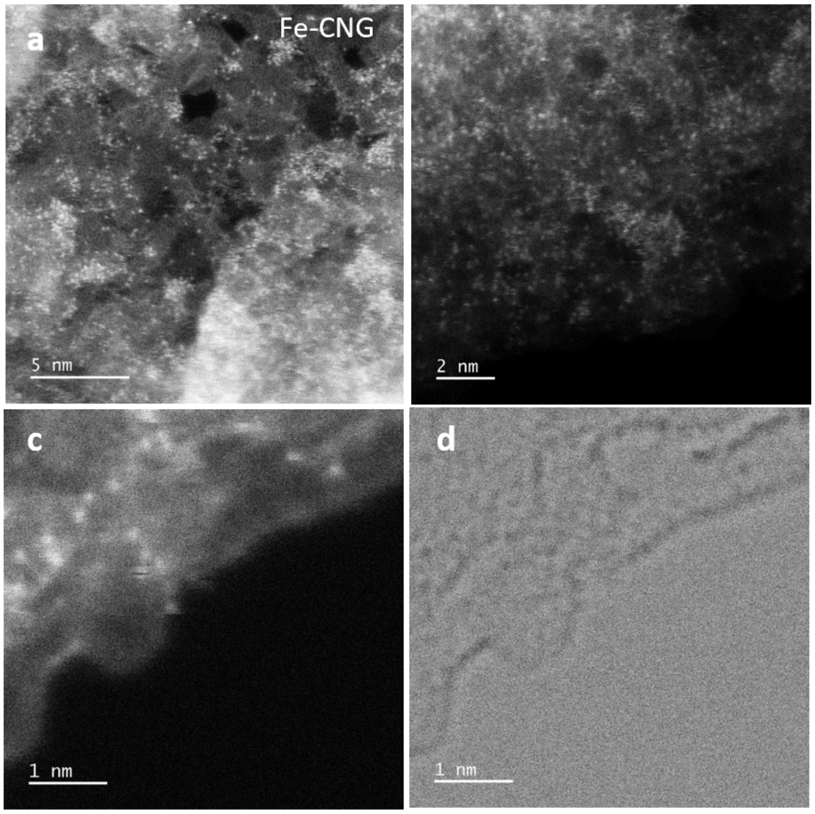

Figure S11. (a-c) HAADF-STEM images of Fe-CNG at different magnifications, where the bright dots are the Fe atoms. (d) Corresponding BF-STEM images of (c), which shows the graphitic structure of the support material, the dark dots are the Fe atoms.

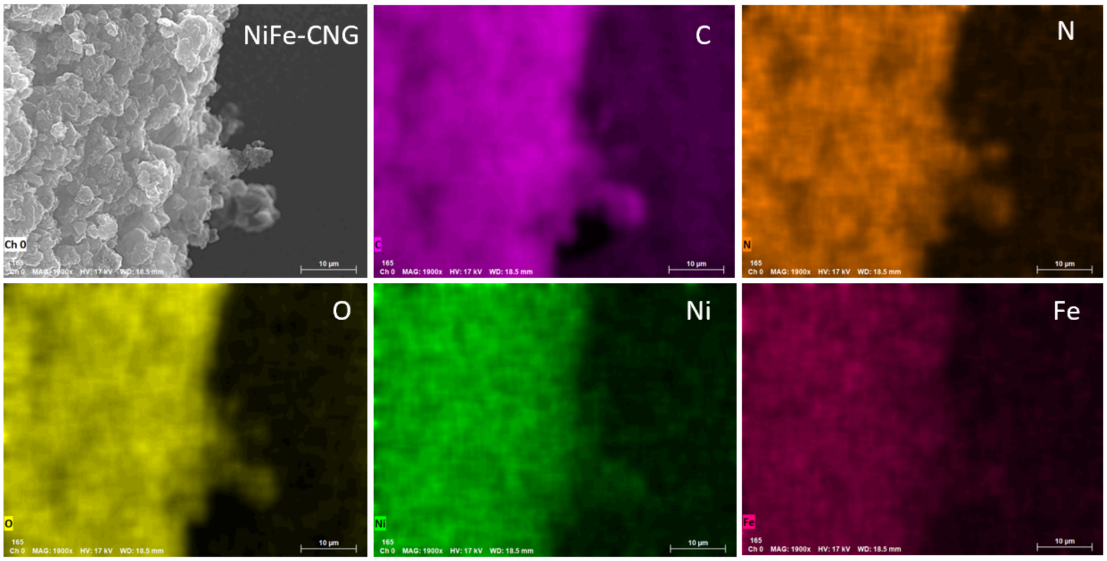

Figure S12. SEM images and EDS elemental mappings of NiFe-CNG.

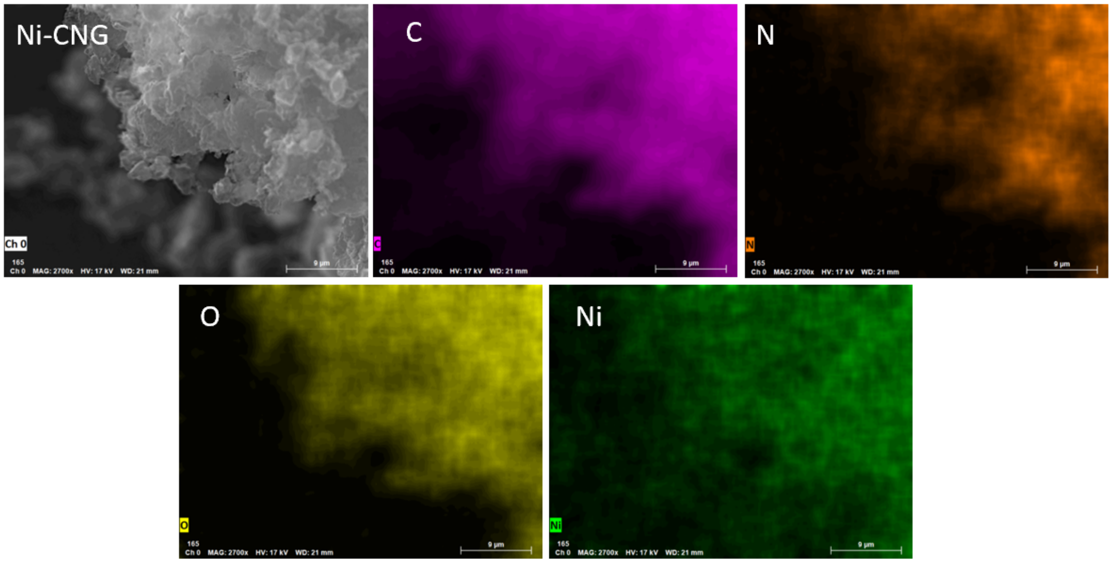

Figure S13. SEM images and EDS elemental mappings of Ni-CNG.

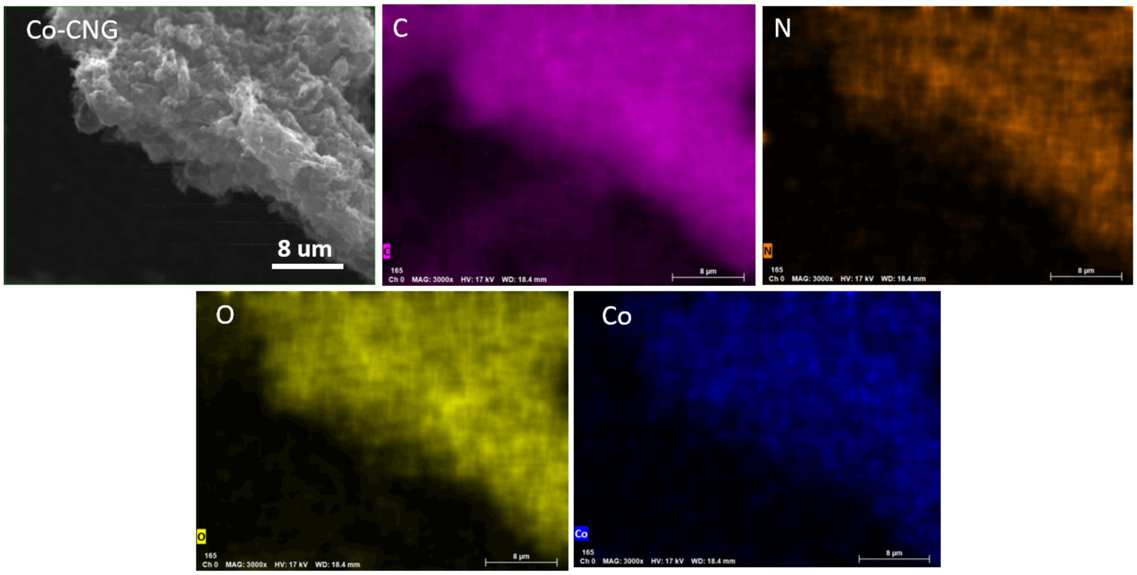

Figure S14. SEM images and EDS elemental mappings of Co-CNG.

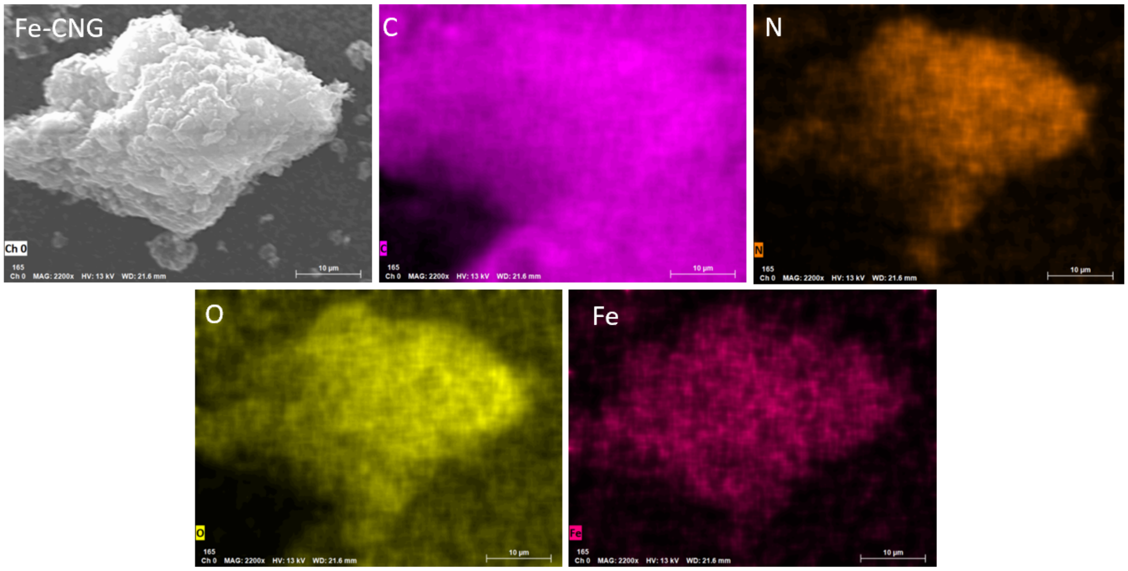

Figure S15. SEM images and EDS elemental mappings of Fe-CNG.

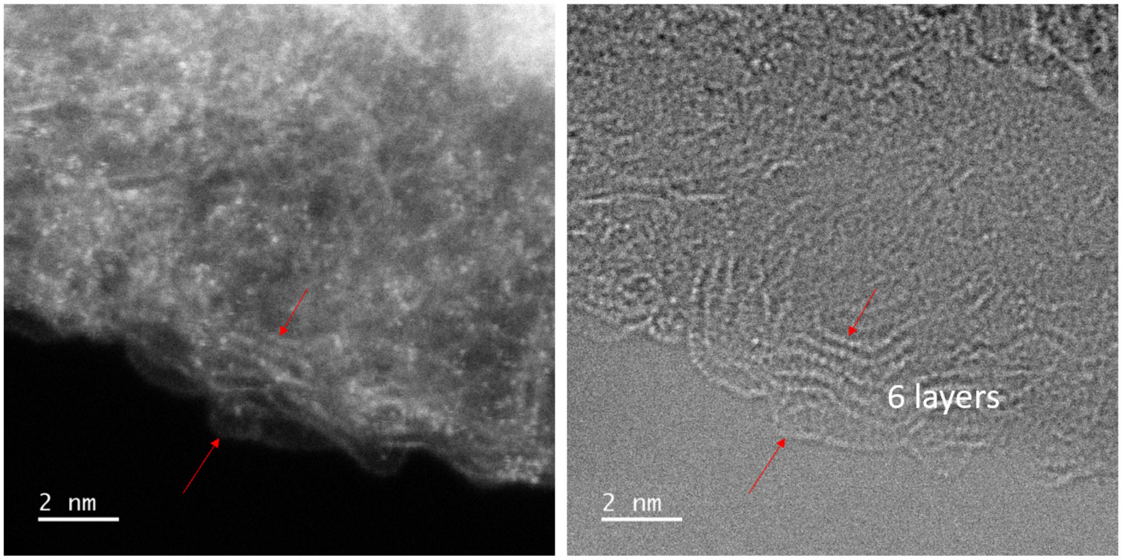

Figure S16. HAADF-STEM (left) and BF-STEM (right) images of NiFe-CNG showing 6 graphene layers. The BF-STEM image shows the graphitic structure of the support material, the dark dots are the Ni and Fe atoms.

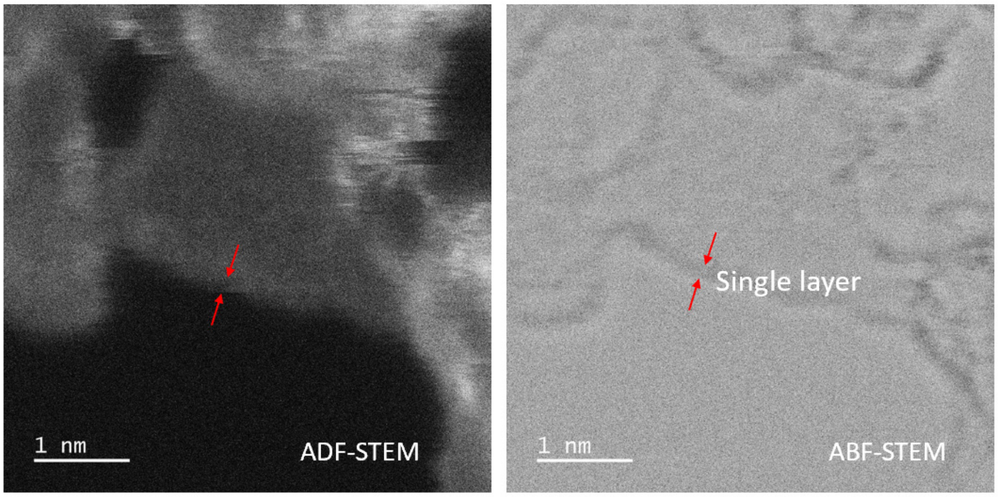

Figure S17. HAADF-STEM (left) and BF-STEM (right) images of NiFe-CNG, which potentially shows a single layer. The BF-STEM image shows the graphitic structure of the support material, the dark dots are the Ni and Fe atoms.

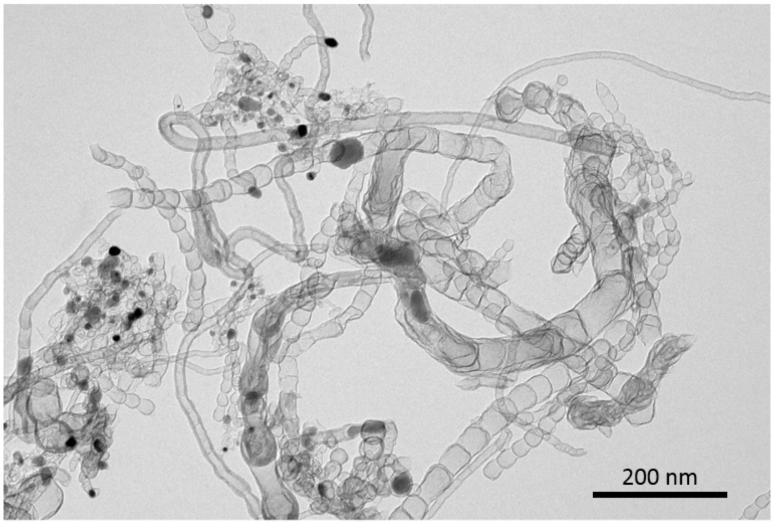

Figure S18. TEM image of Ni-CN. Ni nanoparticles are encapsulated into carbon nanotubes after the pyrolysis process in the absence of glucose.

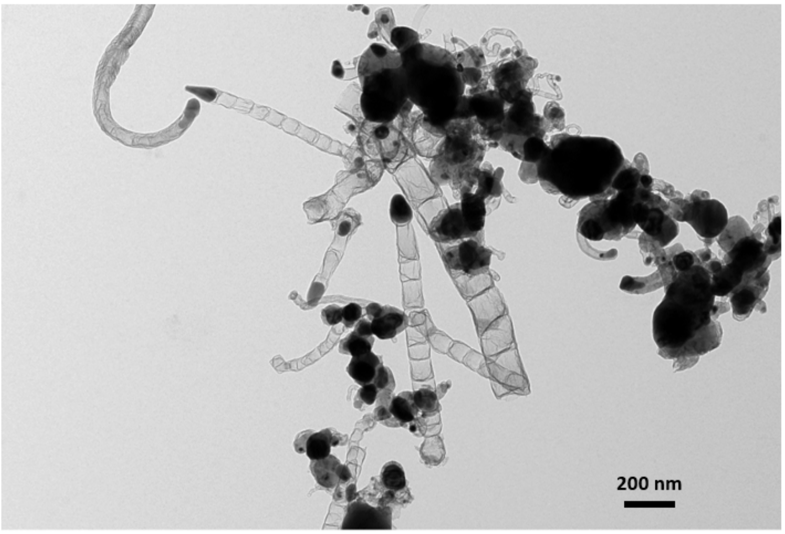

Figure S19. TEM image of Co-CN. Co nanoparticles are encapsulated into carbon nanotubes after the pyrolysis process in the absence of glucose.

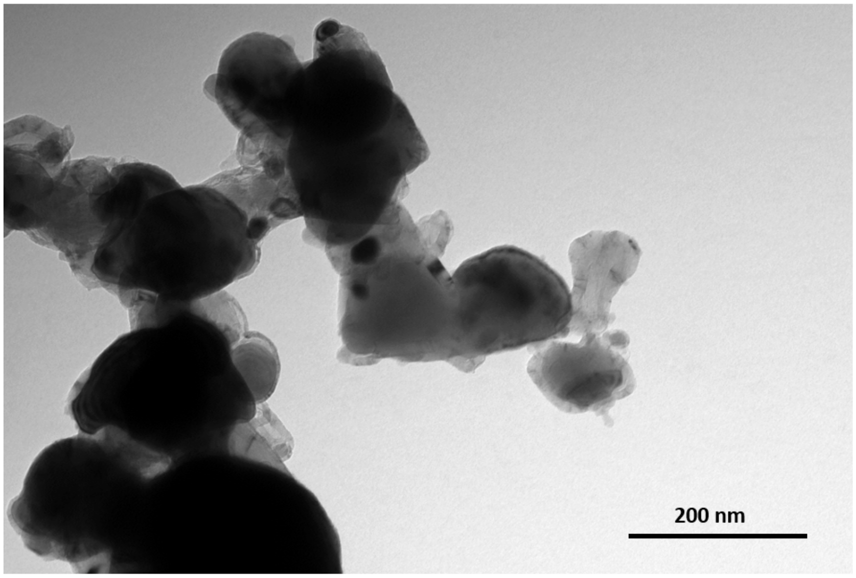

Figure S20. TEM image of Fe-CN. Fe nanoparticles are encapsulated into carbon layers after the pyrolysis process in the absence of glucose.

The formation of N-doped CNT encapsulated structures follows a "reduction–nucleation–growth" mechanism. When the temperature is raised above 600 ºC, the release of N atoms in g-C_3N_4 starts and further defective carbon structures are produced. Meanwhile, the transition metal cations are reduced as well into metallic NPs under an inert atmosphere. The defective carbon is then adsorbed on the surface of these NPs and starts to nucleate, while the as-formed metallic NPs can subsequently act as catalysts for CNT growth. The diameters of the final CNTs are dominated to a large extent by the size of the NPs. As can be seen from TEM images (Figures S18-20), the sizes of Ni and Co NPs are much smaller than those of Fe NPs. Therefore, we suppose that the reasons why Fe^{3+} ions cannot convert g-C_3N_4 into CNTs may be linked to the formation of larger Fe NPs during the pyrolysis, which hinders the nucleation process.

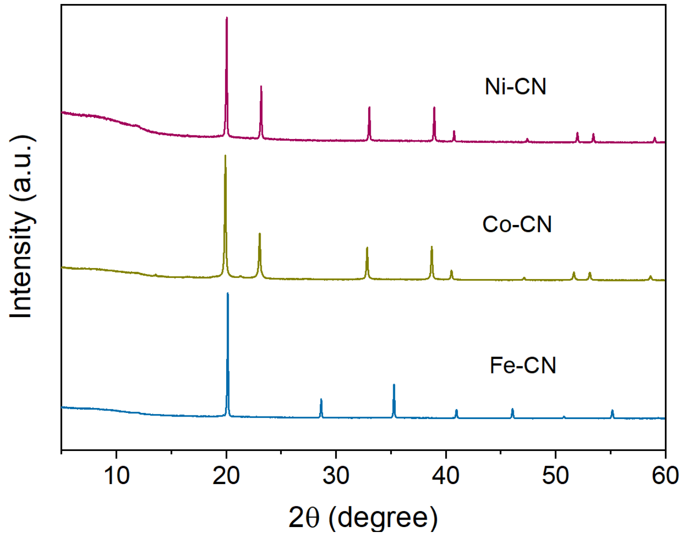

Figure S21. PXRD patterns of Ni-CN, Co-CN, and Fe-CN in the absence of glucose during the synthesis. The results show that Ni, Co, and Fe alloys were formed without adding glucose.

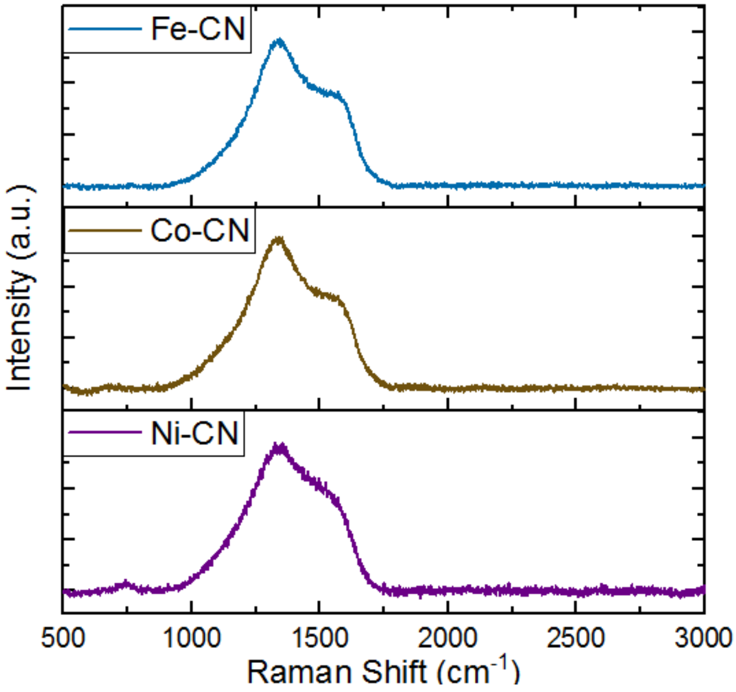

Figure S22. Raman spectra of Ni-CN, Co-CN, and Fe-CN without glucose being present during the synthesis. The results show the formation of a graphene-like structure after pyrolysis.

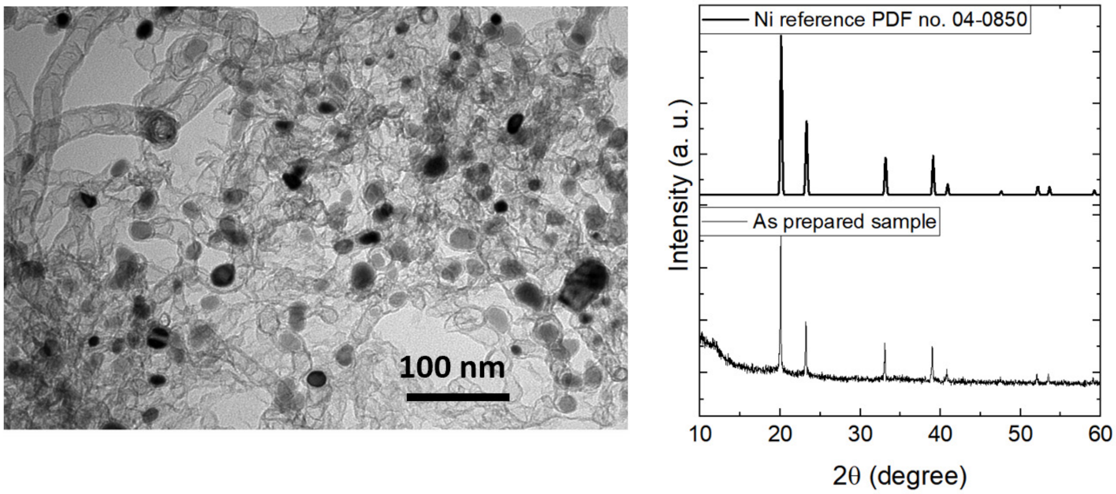

Figure S23. TEM image (left) and PXRD pattern (right) of the sample using nickel acetylacetonate and g-C_3N_4 as precursors to exclude the influence of glucose. The results show that metal nanoparticles are formed.

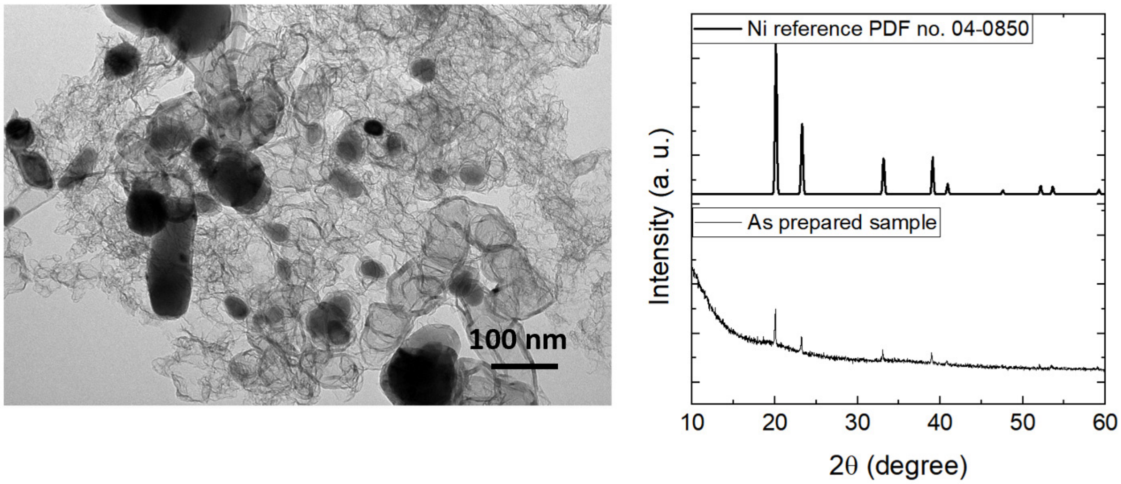

Figure S24. TEM image (left) and PXRD pattern (right) of the sample using nickel acetylacetonate, glucose and g-C_3N_4 as the precursor to exclude the influence of primary chelating effect of glucose.

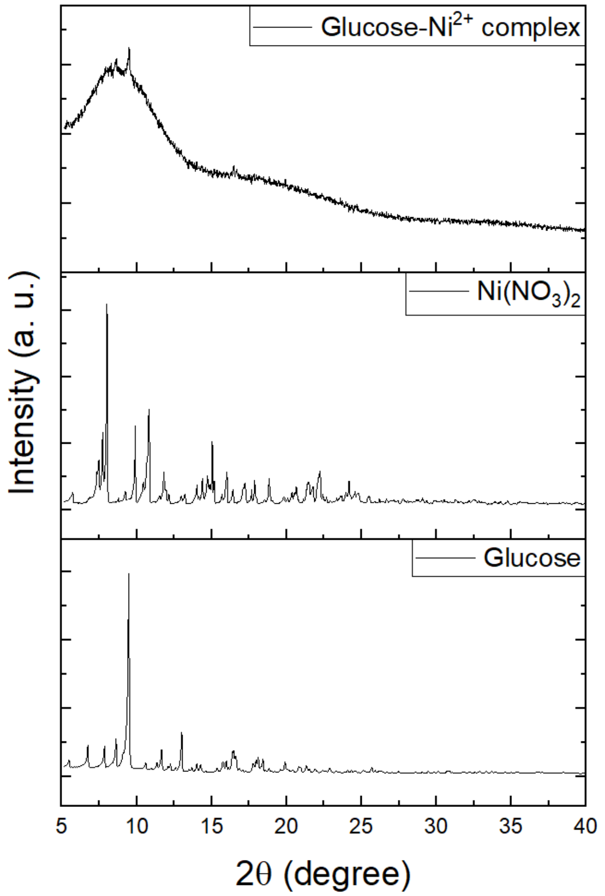

Figure S25. PXRD patterns of glucose-Ni complex, $Ni(NO_3)_2$, and glucose.

In order to investigate the chelating effect of glucose, two control experiments were conducted based on the general synthetic route (see experimental part). In the first experiment, we substituted nickel salt and glucose with nickel acetylacetonate. In the second experiment, we replaced only the nickel salt with nickel acetylacetonate. In nickel acetylacetonate, nickel ions are strongly coordinated to acetylacetone, which can replace glucose as a ligand and thereby eliminate the interaction between glucose and nickel ions. The TEM images and PXRD patterns show that both experiments generated considerable amounts of metal nanoparticles (Figures S23, S24). To further confirm the indispensable role of the chelation of metal ions by glucose, we prepared the glucose-Ni ion complex in the absence of g-C_3N_4, and compared the PXRD pattern with bare glucose and $Ni(NO_3)_2$ (Figure S25). The results show that an amorphous complex was formed after evaporation of water, which corroborates the interaction between glucose and Ni ions.

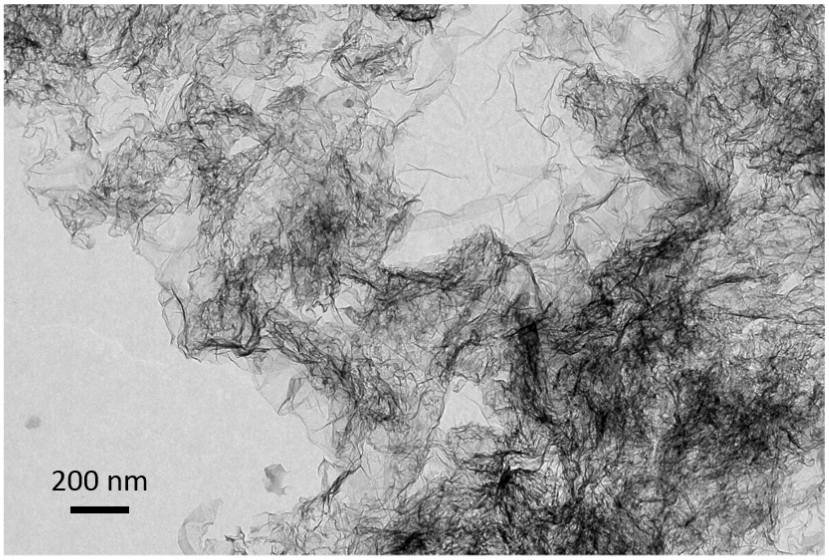

Figure S26. TEM image of glucose-CN showing a typical graphene layer morphology.

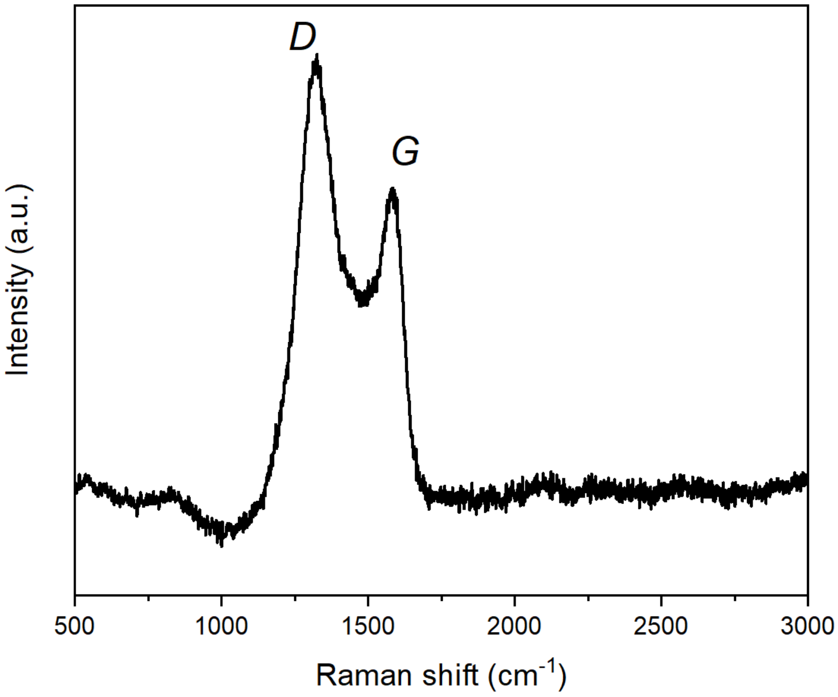

Figure S27. Raman spectrum of glucose-CN showing typical D and G bands of a graphene material.

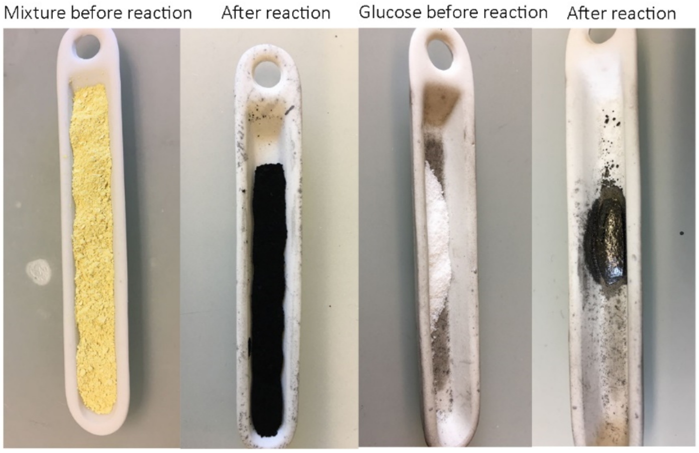

Figure S28. Representative photos of the samples before and after pyrolysis at 900 ºC; left: mixture of g-C_3N_4 powder, glucose and Ni^{2+}; right: pure glucose.

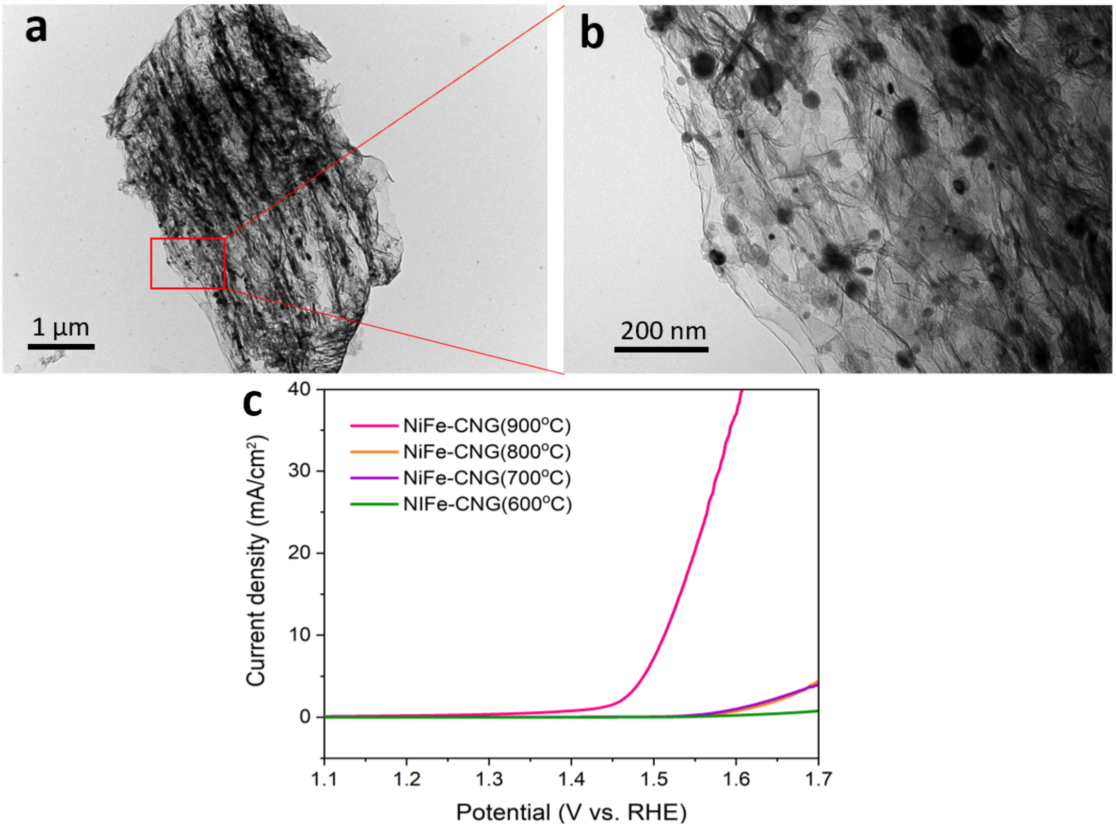

Figure S29. (a-b) TEM images of NiFe-CNG-1000 after treatment at 1000 ºC shows metal nanoparticles attached to graphene layers. (c) OER performance of NiFe-CNG prepared at different temperatures. Metal atoms aggregate into particles when the temperature is increased to 1000 ºC as shown in Figure S29a-b. The activities of the SACs are also significantly influenced by the synthesis temperatures. This is due to the low synthetic temperature leading to poor conductivity as a result of incomplete carbonization of g-C_3N_4 (Figure S29c).

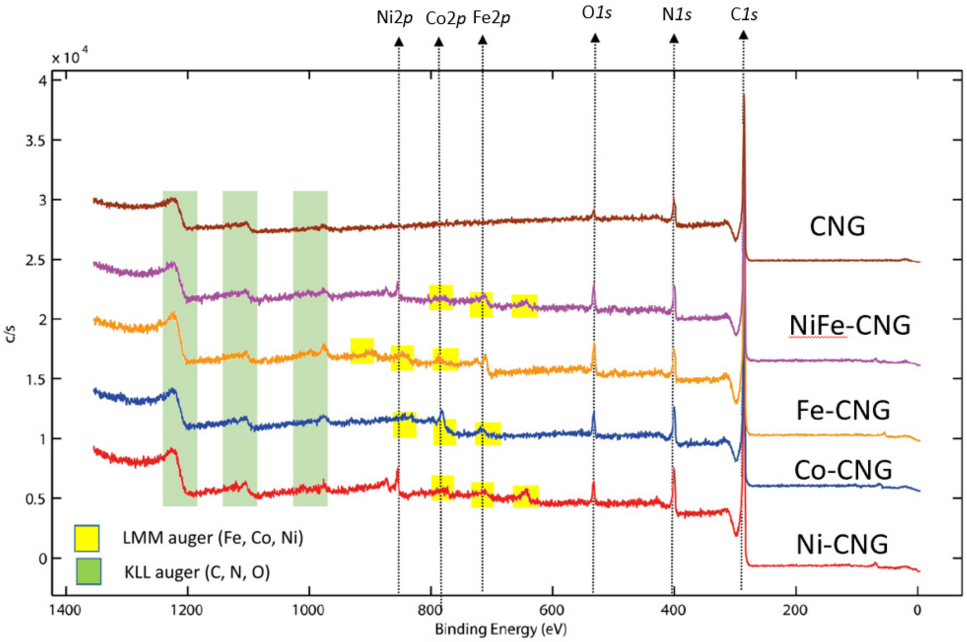

Figure S30. XPS survey spectra of CNG, Ni-CNG, Co-CNG, Fe-CNG, and NiFe-CNG.

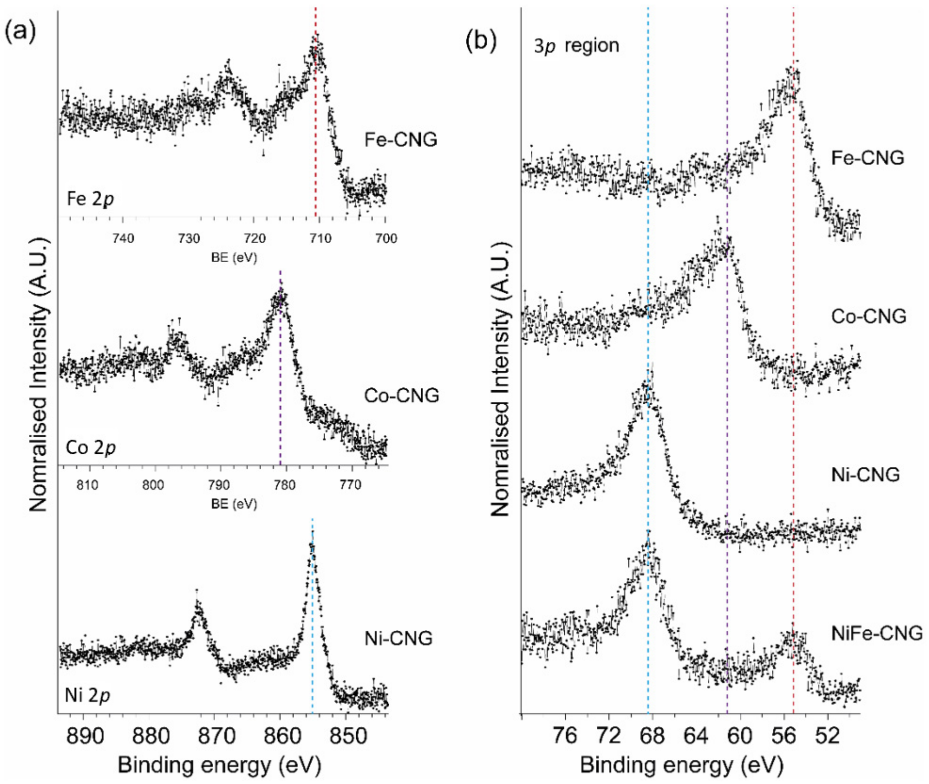

Figure S31. High-resolution XPS 2*p* and 3*p* spectra of Ni-CNG, Co-CNG, Fe-CNG and NiFe-CNG.

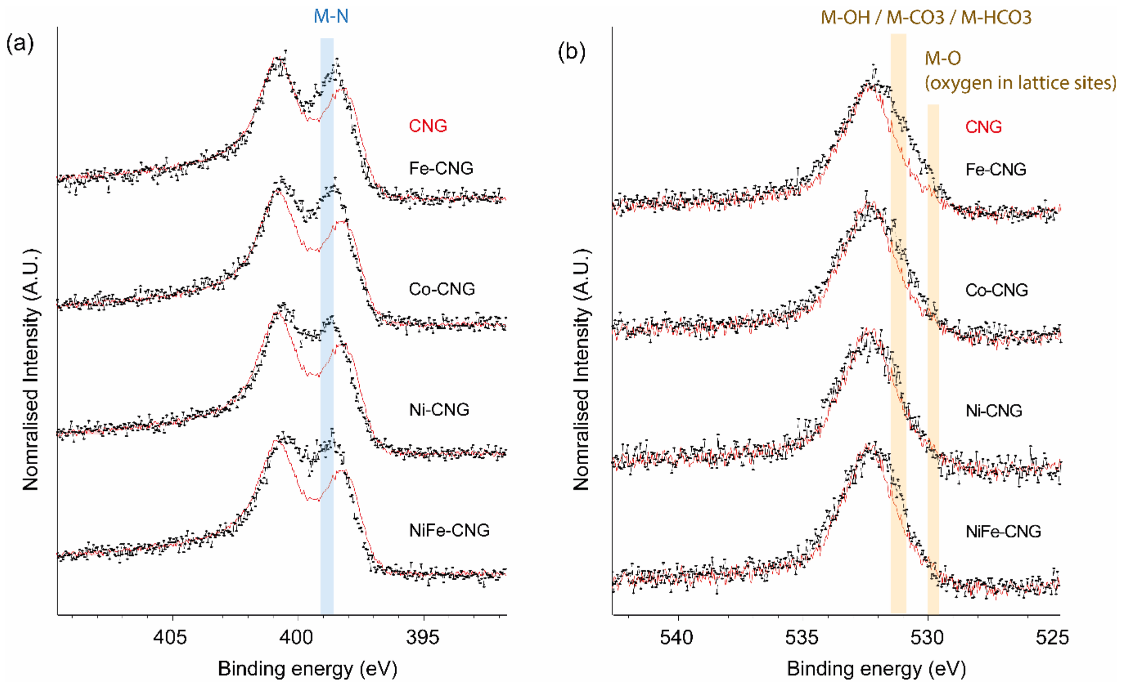

Figure S32. High-resolution XPS spectra of CNG, Ni-CNG, Co-CNG, Fe-CNG and NiFe-CNG: (a) N 1*s* spectra and (b) O 1*s* spectra. Information on the BE ranges highlighted in the N 1*s* and O 1*s* spectral regions are discussed in Note 1.

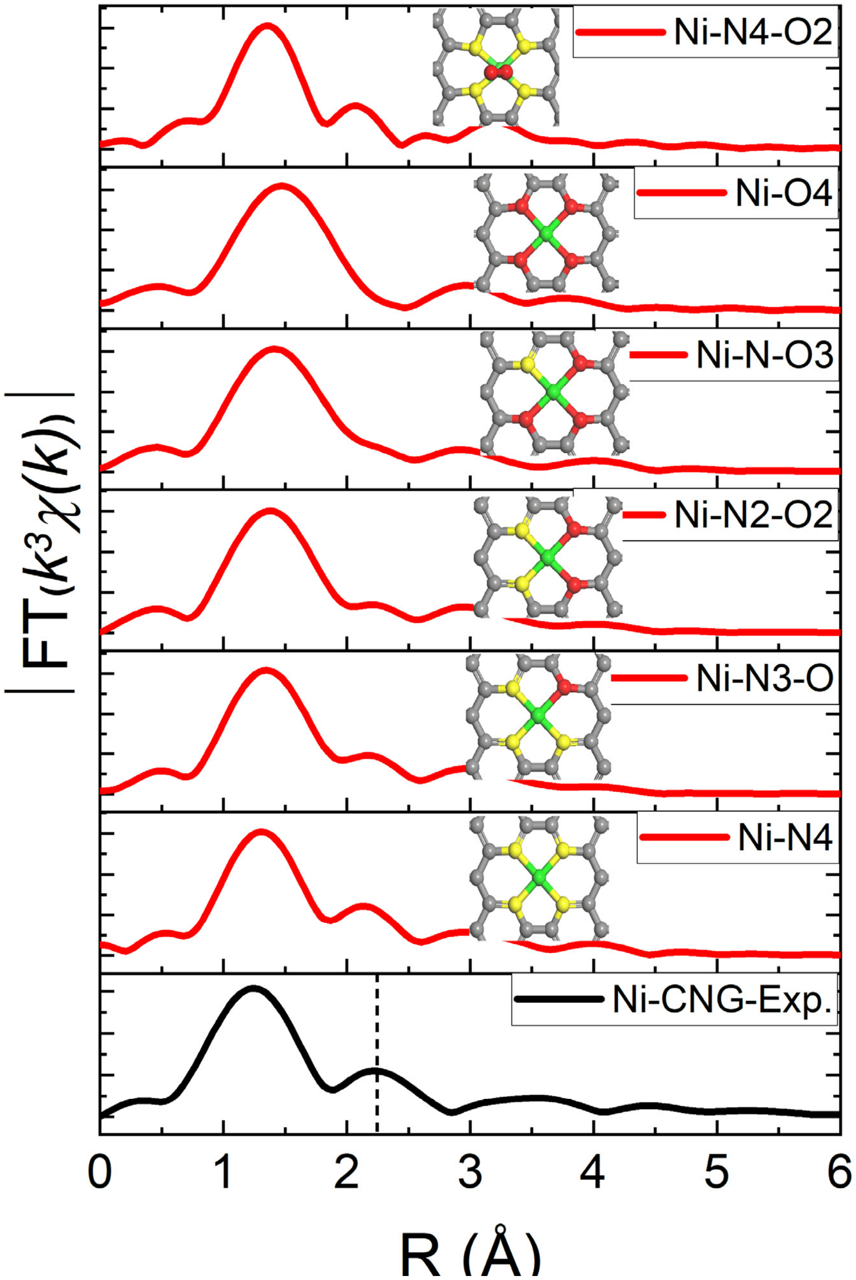

Figure S33. Experimental and calculated FT-EXAFS spectra of Ni-CNG.

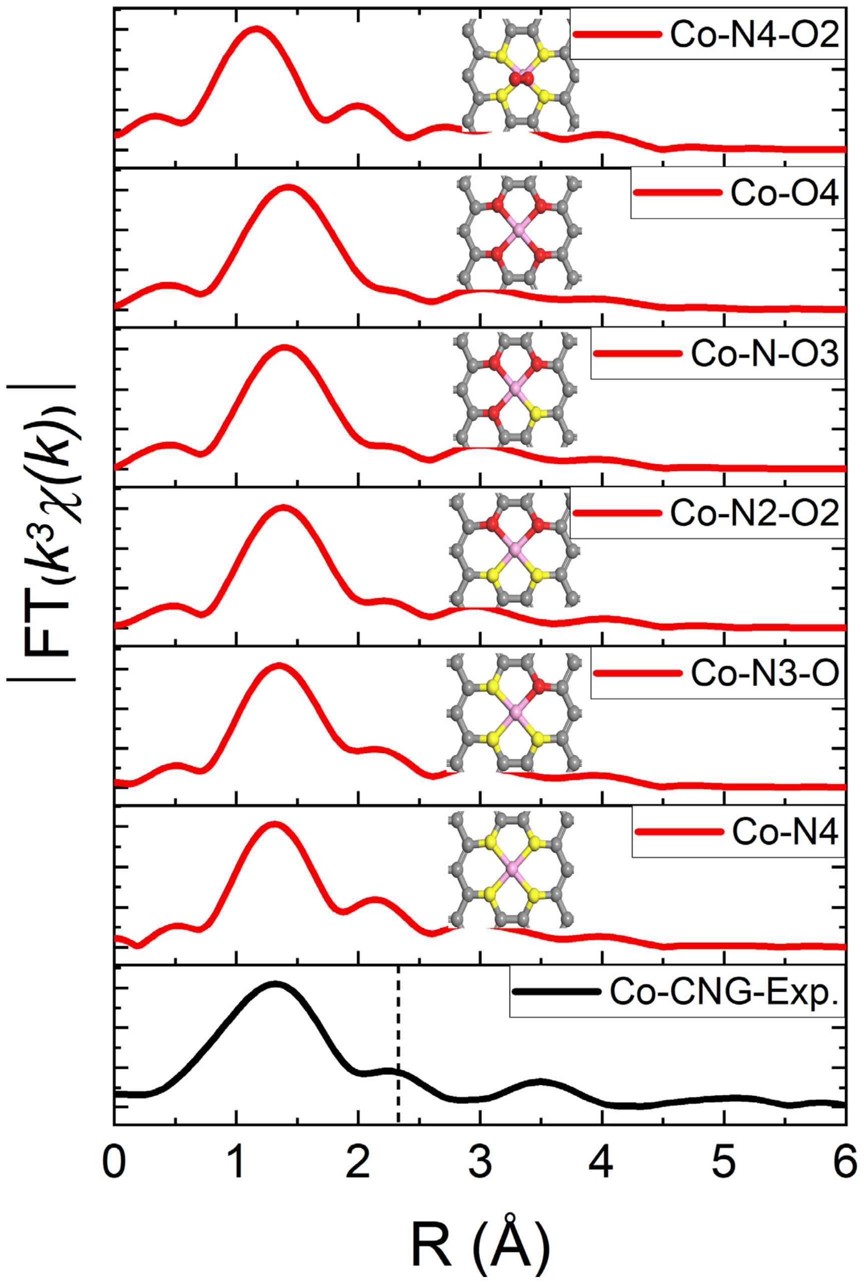

Figure S34. Experimental and calculated FT-EXAFS spectra of Co-CNG.

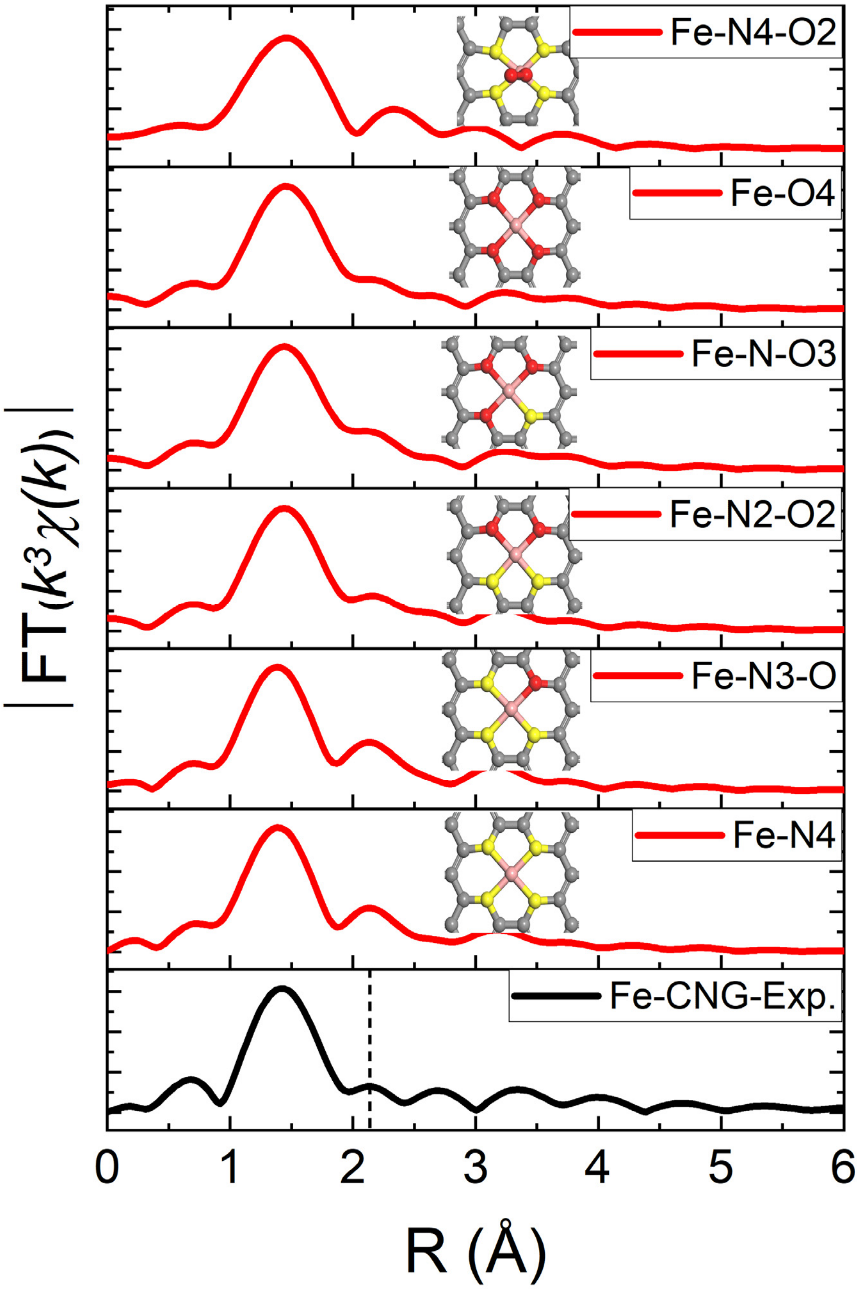

Figure S35. Experimental and calculated FT-EXAFS spectra of Fe-CNG.

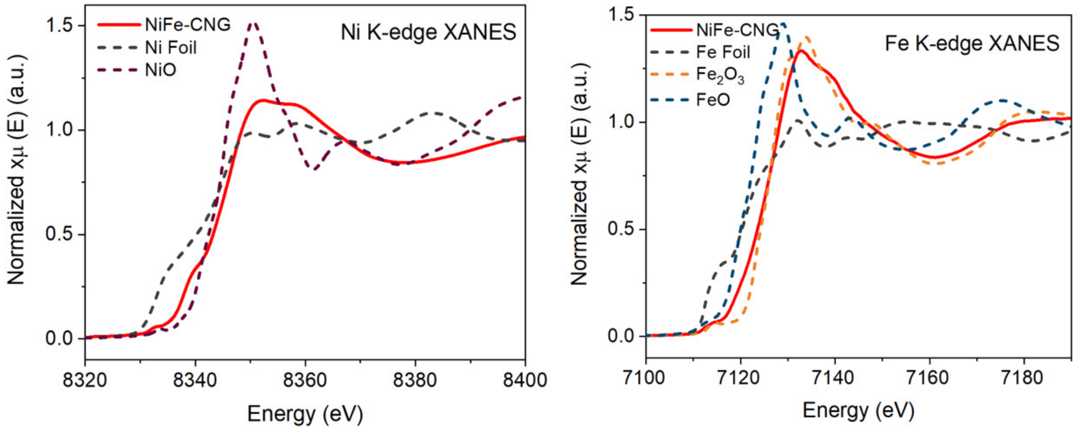

Figure S36. Ni (left) and Fe (right) K-edge XANES spectra of NiFe-CNG.

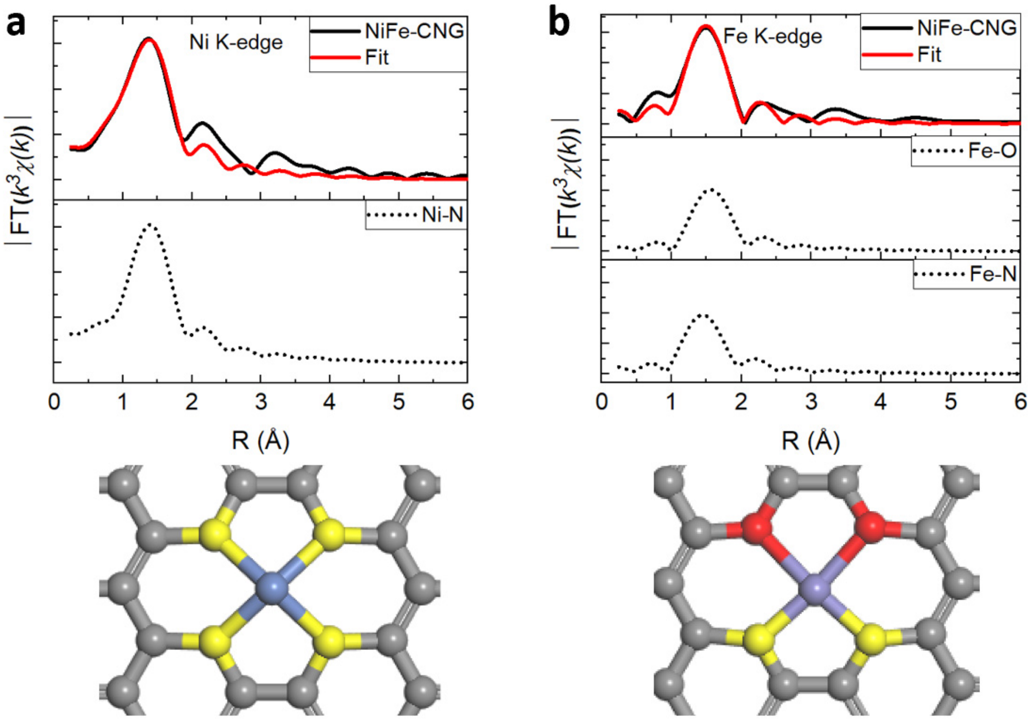

Figure S37. Fitting of (a) Ni and (b) Fe K-edge FT-EXAFS spectra of NiFe-CNG (top) and the corresponding models used for the fitting (bottom: grey, red, yellow, light blue and purple spheres represent C, O, N, Ni and Fe, respectively).

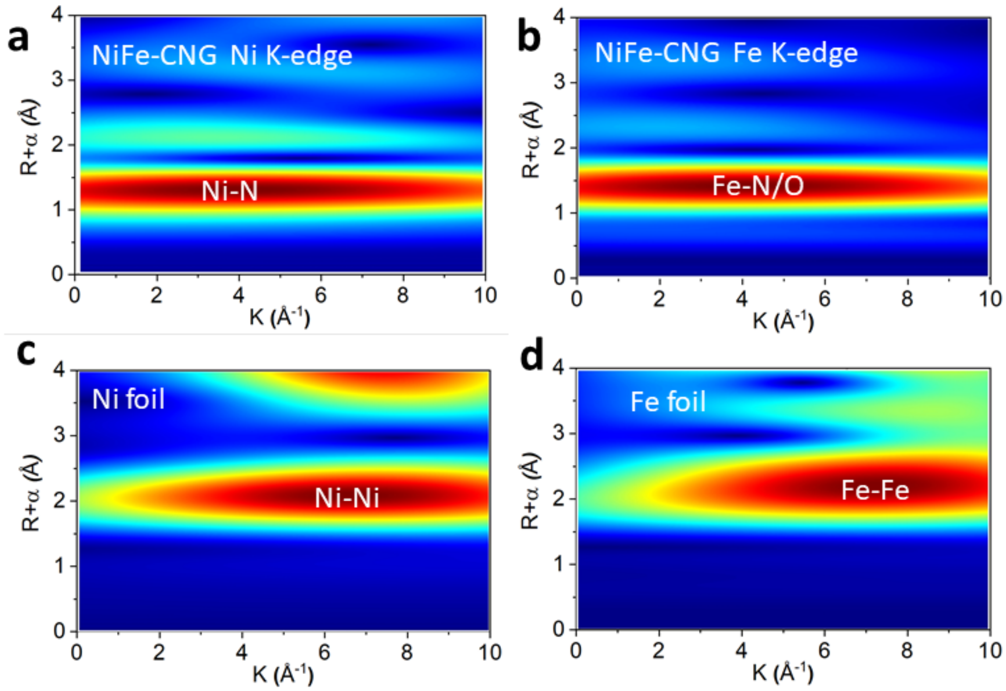

Figure S38. WT-EXAFS spectra of NiFe-CNG: (a) Ni K-edge and (b) Fe K-edge, (c) Ni foil and (d) Fe foil.

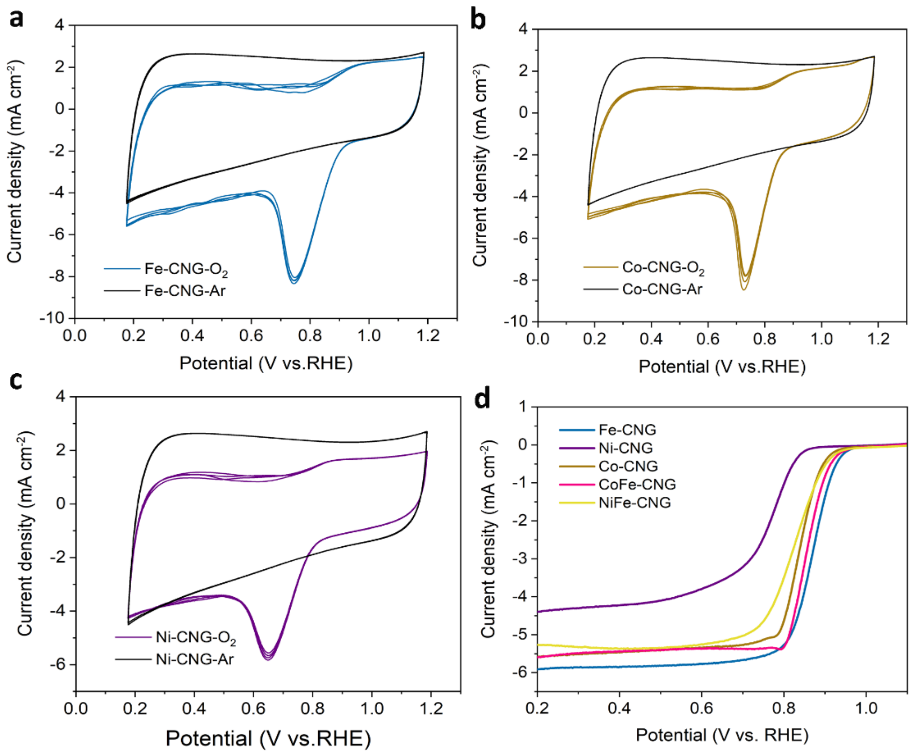

Figure S39. (a-c) Cyclic voltammetry curves of Fe-CNG, Co-CNG, and Ni-CNG in Ar and O_2 saturated 0.1 M KOH solutions. (d) LSV curves of Fe-CNG, Co-CNG, Ni-CNG, CoFe-CNG, and NiFe-CNG.

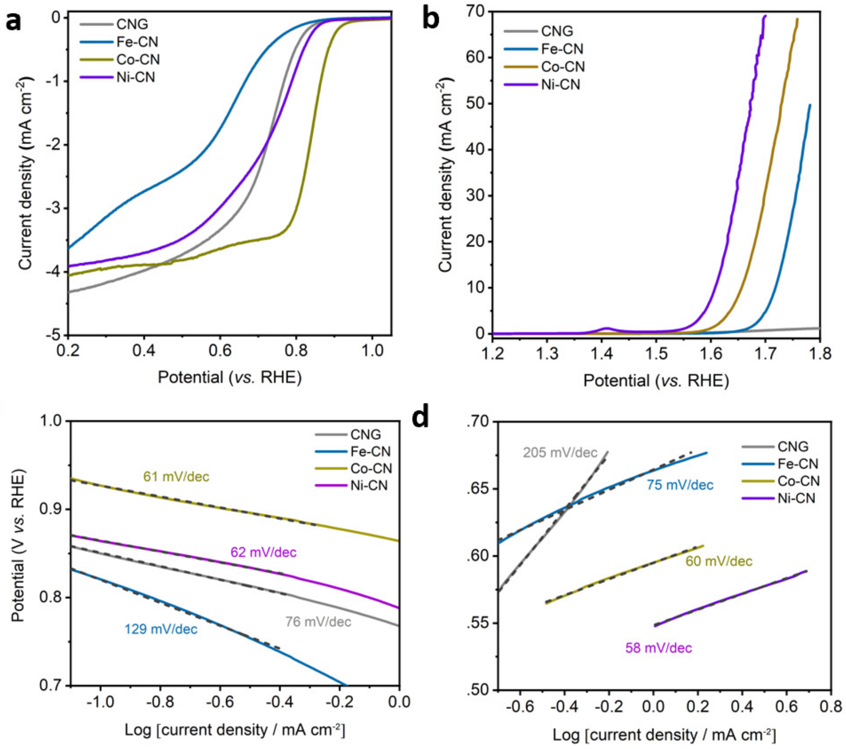

Figure S40. Electrocatalytic performance of CNG, Fe-CN, Co-CN and Ni-CN: (a-b) ORR and OER performance, (c-d) corresponding Tafel slopes.

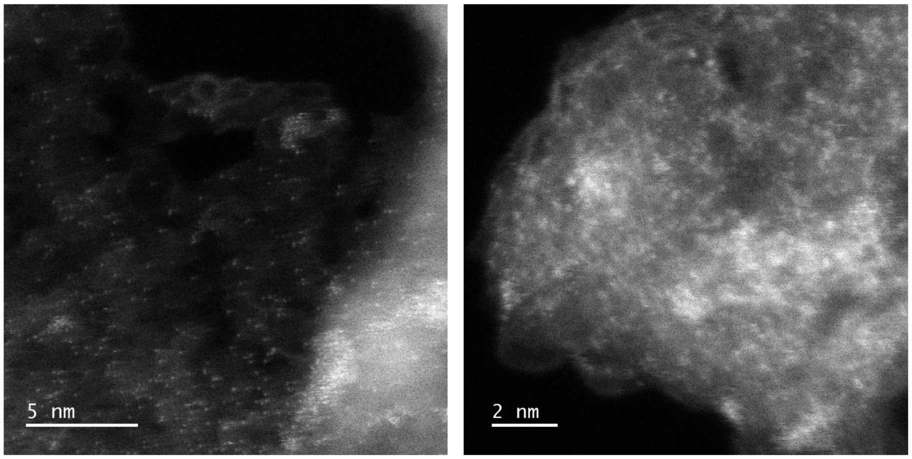

Figure S41. HAADF-STEM images of CoFe-CNG at different magnifications. The uniformly distributed bright dots represent metal atoms.

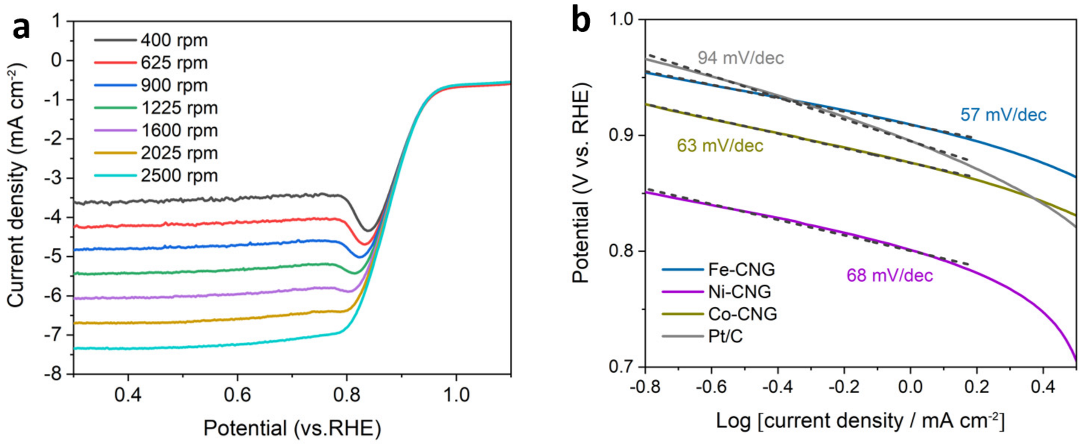

Figure S42. (a) LSV curves of Fe-CNG with a scan rate of 5 mV s^{-1} at different rotation rates. (b) Tafel plots of Fe-CNG, Ni-CNG, Co-CNG and Pt/C extracted from the corresponding ORR polarization curves.

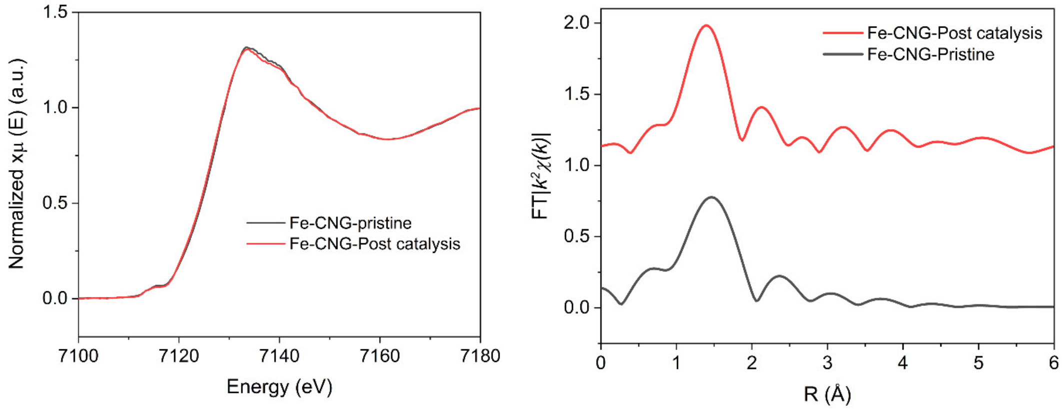

Figure S43. XANES (left) and FT-EXAFS (right) spectra of Fe-CNG before and after 20000 CV cycles.

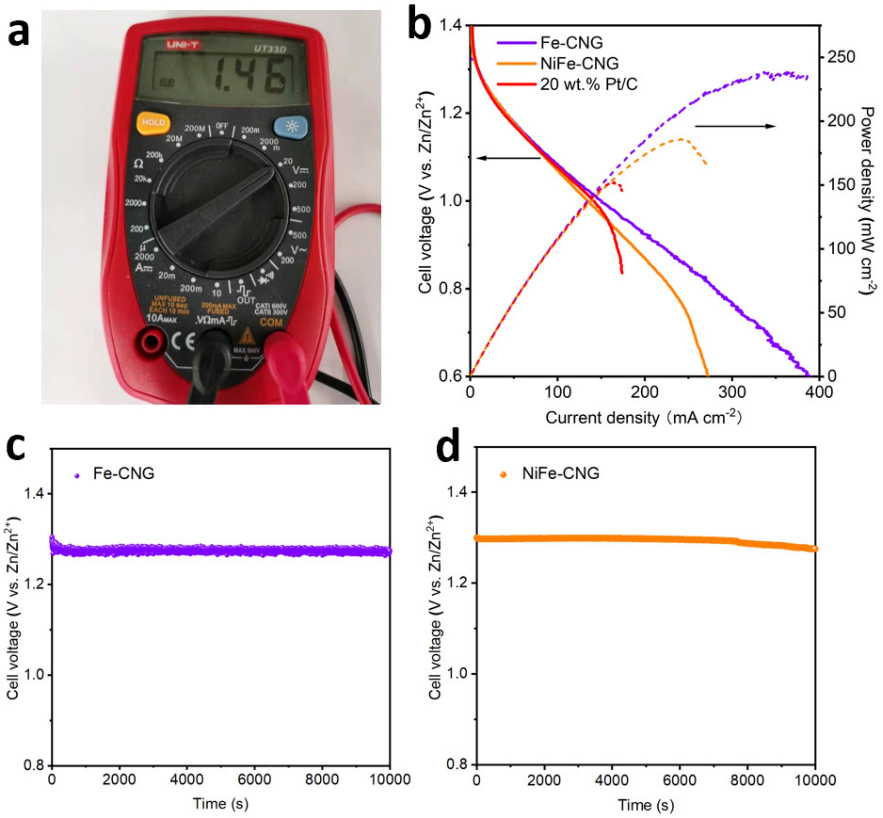

Figure S44. Zn-air battery measurements: (a) Photograph of the open-circuit voltage of the Fe-CNG based Zn-air battery measured with a multimeter. (b) Discharge polarization curves and power density plots of Fe-CNG, NiFe-CNG and Pt/C-based Zn-air batteries. (c-d) Long-term stability of the primary Zn-air battery with Fe-CNG and NiFe-CNG cathode at a current density of 10 mA cm^{-2}.

Zn-air battery application options were investigated using Fe-CNG, NiFe-CNG and commercial 20 wt.% Pt/C as the cathode materials (Figure S44). The open-circuit potential of the as-prepared Fe-CNG is 1.46 V (Figure S44a). The maximum power densities of Fe-CNG and NiFe-CNG are 239.1 mW cm^{-2} and 185.9 mW cm^{-2}, respectively, which outperform those of commercial 20 wt.% Pt/C (152.1 mW cm^{-2}) and most of the recent SAC electrocatalysts (Figure S44b and Table 1). The long-term durability tests for the Fe-CNG and NiFe-CNG-based Zn-air batteries indicated that the catalysts could retain the discharged current density of 10 mA cm^{-2} for over 10,000 s without any obvious voltage decline (Figure S44c-d). The above results demonstrate that Fe-CNG is a potential candidate for practical Zn-air battery applications.

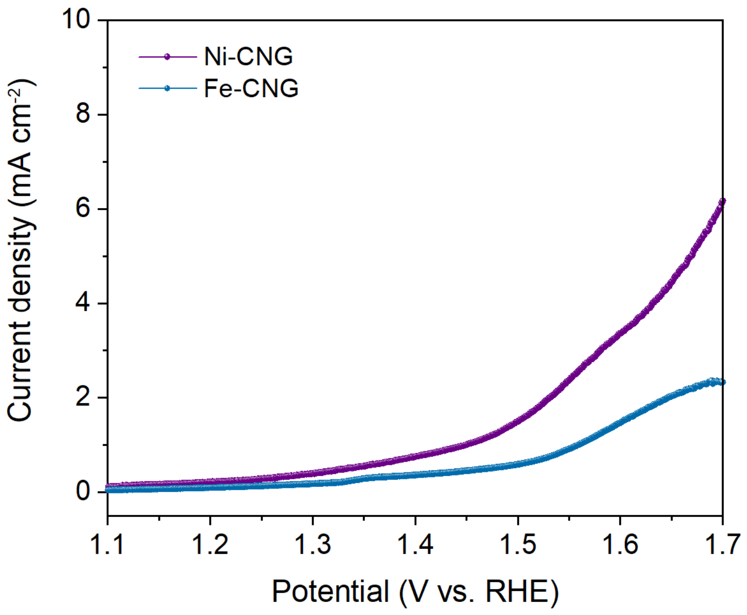

Figure S45. OER polarization curves of Ni-CNG and Fe-CNG showing almost no activity.

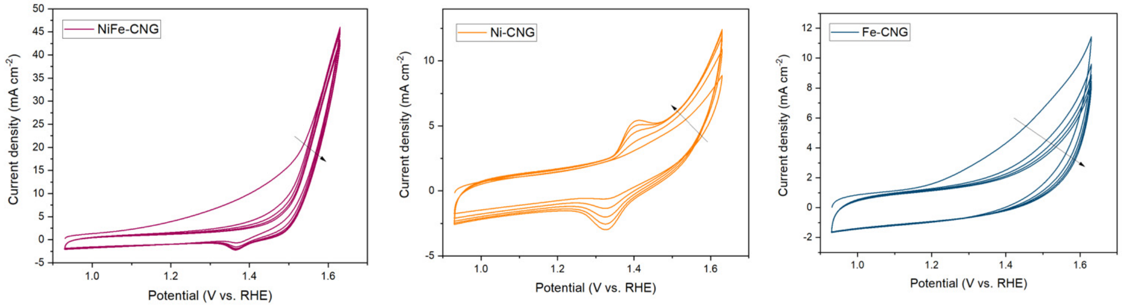

Figure S46. Cyclic voltammetry curves of NiFe-CNG, Ni-CNG and Fe-CNG before LSV measurements.

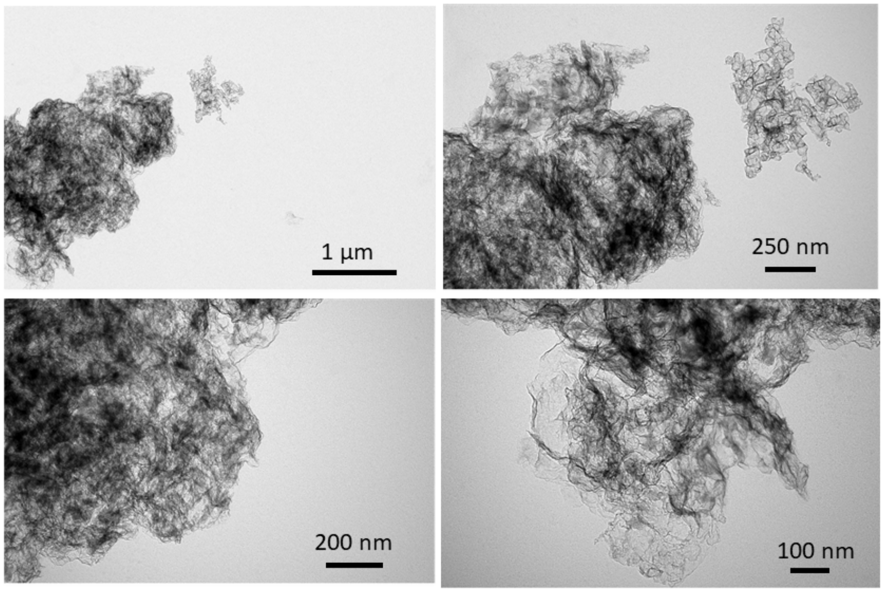

Figure S47. TEM images of NiFe-CNG after the chronoamperometry measurement over 72 h at a current density of 10 mA cm^{-2}.

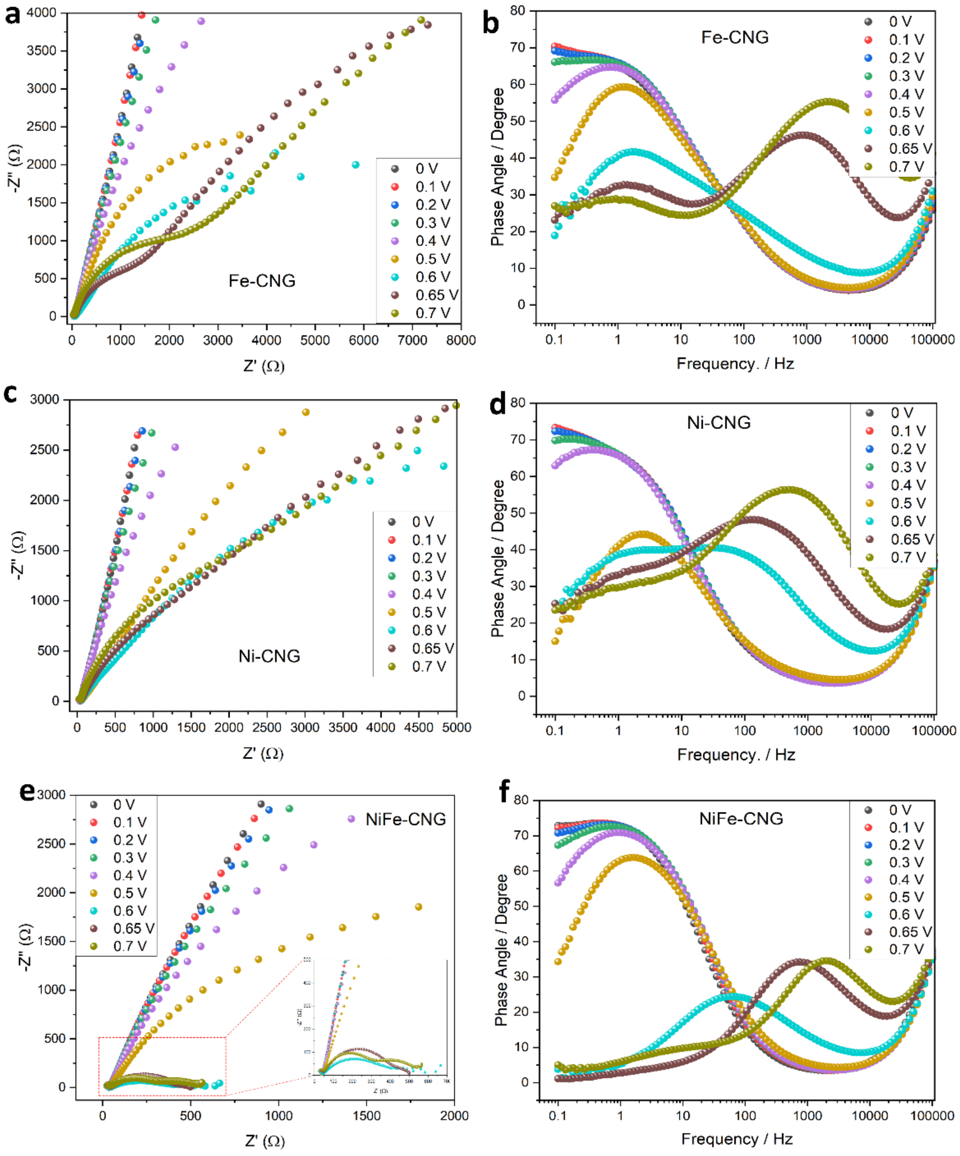

Figure S48. (a, c, e) Electrochemical impedance spectra (Nyquist plots) and (b, d, f,) Bode plots of Fe-CNG, Ni-CNG, and NiFe-CNG

To gain an in-depth understanding of the reaction kinetics, in situ electrochemical impedance spectroscopy (EIS) studies of Fe-CNG, Ni-CNG and NiFe-CNG under different external bias were conducted. First, the Nyquist plots of NiFe-CNG possess the smallest resistance at all the operating potentials (Figure S48a, c, e), indicating the improved kinetics at the electrode-electrolyte interface during OER. Corresponding Bode phase plots show that the phase angle relaxation of NiFe-CNG dramatically decreased with the applied external bias above 0.6 V compared with that of Fe-CNG and Ni-CNG (Figure S48 b, d, f), which further reveals that the electron transfer in NiFe-CNG is more favorable especially at high potentials.

To further qualitatively analyze the charge transfer mechanisms of the OER process, we recorded the Bode phase plots of the catalysts for increasing applied potentials. There are two distinct peaks observed in the frequency window of 0.1-10,000 Hz (Figure S48b, d, f), where the peak in the frequency window of 100-10,000 Hz can be assigned to charge transfer due to double-layer capacitance and the frequency window of 0.1-100 Hz corresponds to the surface intermediates involved in Faradaic processes.[15]

With the increase in the externally applied bias, the new electrochemical process associated with the double layer capacitance appears at high frequency in all catalysts. At high applied potential, the electrochemical process at low frequency (0.1-100 Hz) disappeared, while it is still preserved for Ni-CNG and Fe-CNG, revealing that NiFe-CNG undergoes a transition from charge transfer involving surface intermediates to double-layer capacitance related charge transfer. However, the charge transfer mechanism of Ni-CNG and Fe-CNG is a combination of relaxation components of charge transfer processes involving double layer and surface intermediates. In NiFe-CNG, the main electrochemical process is based on the relaxation components of charge transfer processes involving double layer at high frequency with applying higher applied bias in the system. The Nyquist plots (Figure S48 a, c, e) clearly show a significant decrease in overall resistance of NiFe-CNG compared with Ni-CNG and Fe-CNG indicating that the Faradaic process involved in double-layer capacitance is more strongly contributing to the overall OER process.[16]

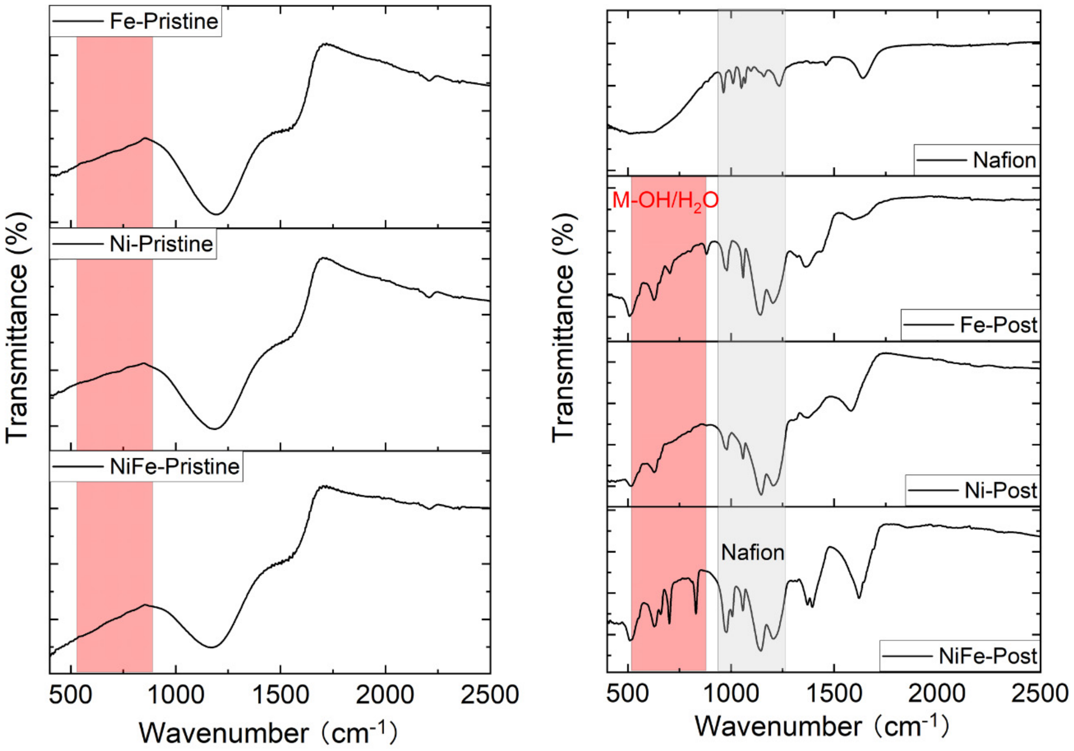

Figure S49. FTIR spectra of the dry samples and the samples after 10 CV cycles.

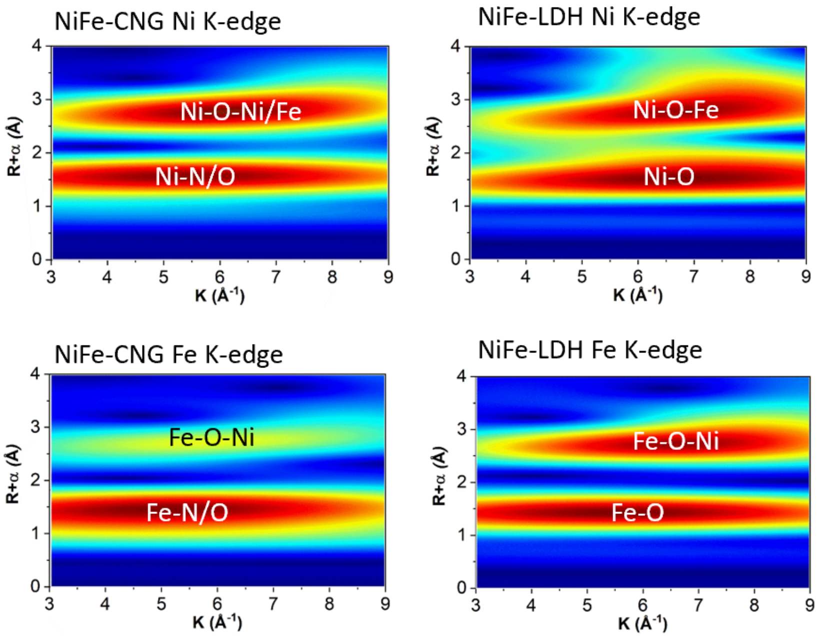

Figure S50. Wavelet transforms for the k^3-weighted EXAFS signals of NiFe-CNG (left) at applied potential of 1.5 V (vs. RHE) and newly prepared NiFe LDH (right).

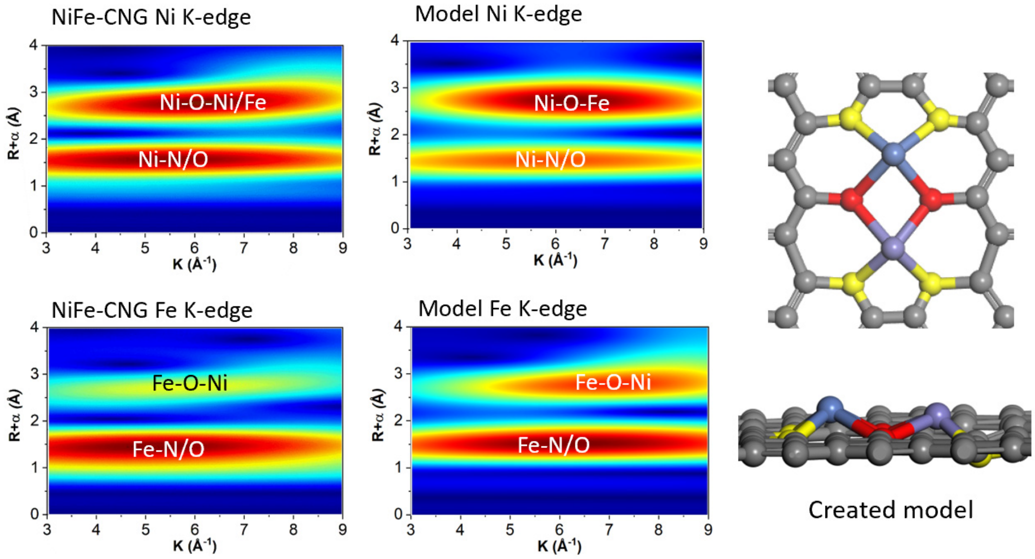

Figure S51. Wavelet transforms for the k^3-weighted EXAFS signals of NiFe-CNG (left) at applied potential of 1.5 V (vs. RHE), and of the proposed structure (right: grey, red, yellow, light blue and purple spheres represent C, O, N, Ni and Fe, respectively).

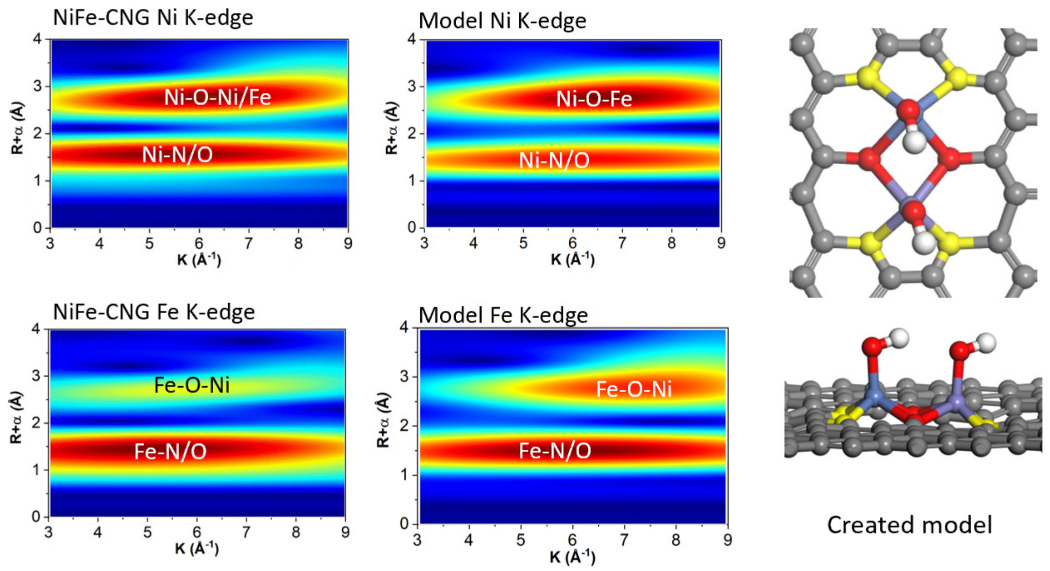

Figure S52. Wavelet transforms for the k^3-weighted EXAFS signals of NiFe-CNG (left) at applied potential of 1.5 V (vs. RHE), and of the proposed structure (right) with two OH^- anions chemisorbed at Ni and Fe metal centers (grey, red, yellow, white, light blue and purple spheres represent C, O, N, H, Ni and Fe, respectively).

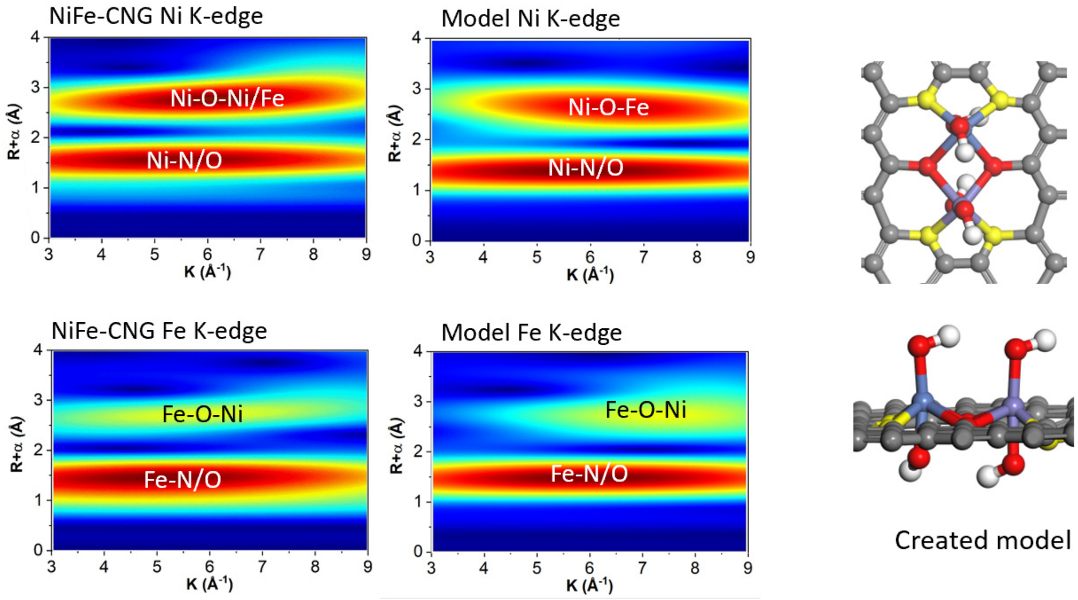

Figure S53. Wavelet transforms for the k^3-weighted EXAFS signals of NiFe-CNG at applied potential of 1.5 V (vs. RHE) (left), and of the proposed structure (right) with four OH^- chemisorbed at Ni and Fe metal centers (grey, red, yellow, white, light blue and purple spheres represent C, O, N, H, Ni and Fe, respectively).

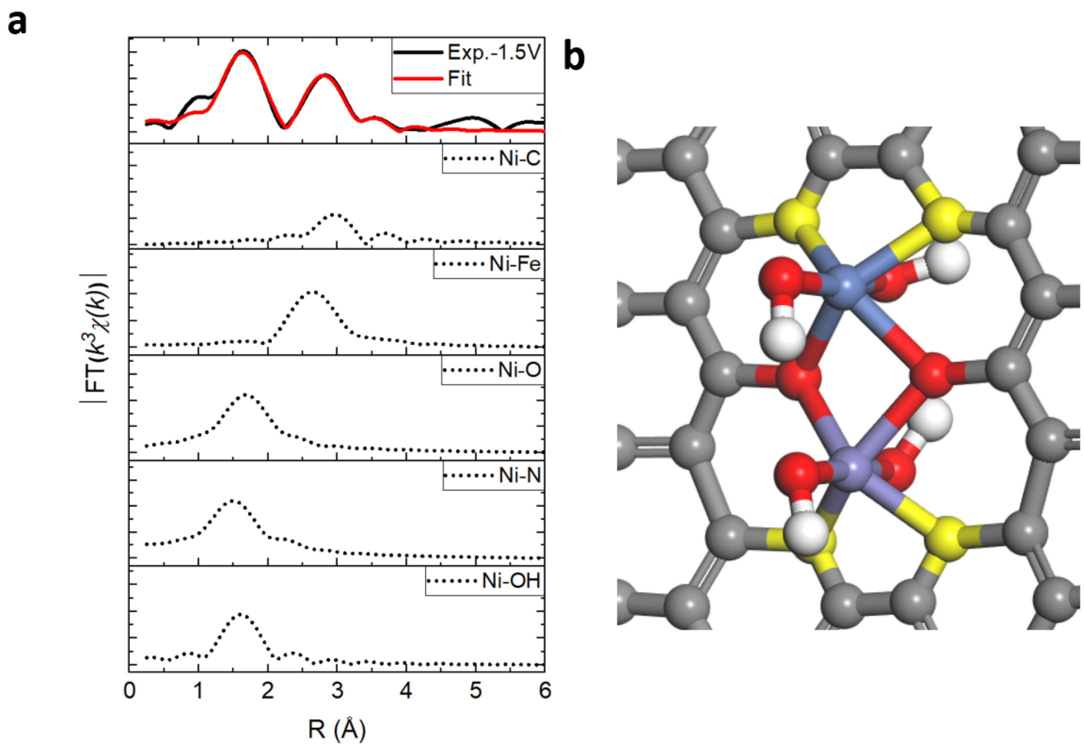

Figure S54. (a) Fitting (red) of the experimental (black) Ni K-edge EXAFS spectra $|FT(k^3\chi(k))|$ of NiFe-CNG at the potential of 1.5 V (vs. RHE) and the corresponding paths. (b) Fitting model (grey, red, yellow, white, light blue and purple spheres represent C, O, N, H, Ni and Fe, respectively).

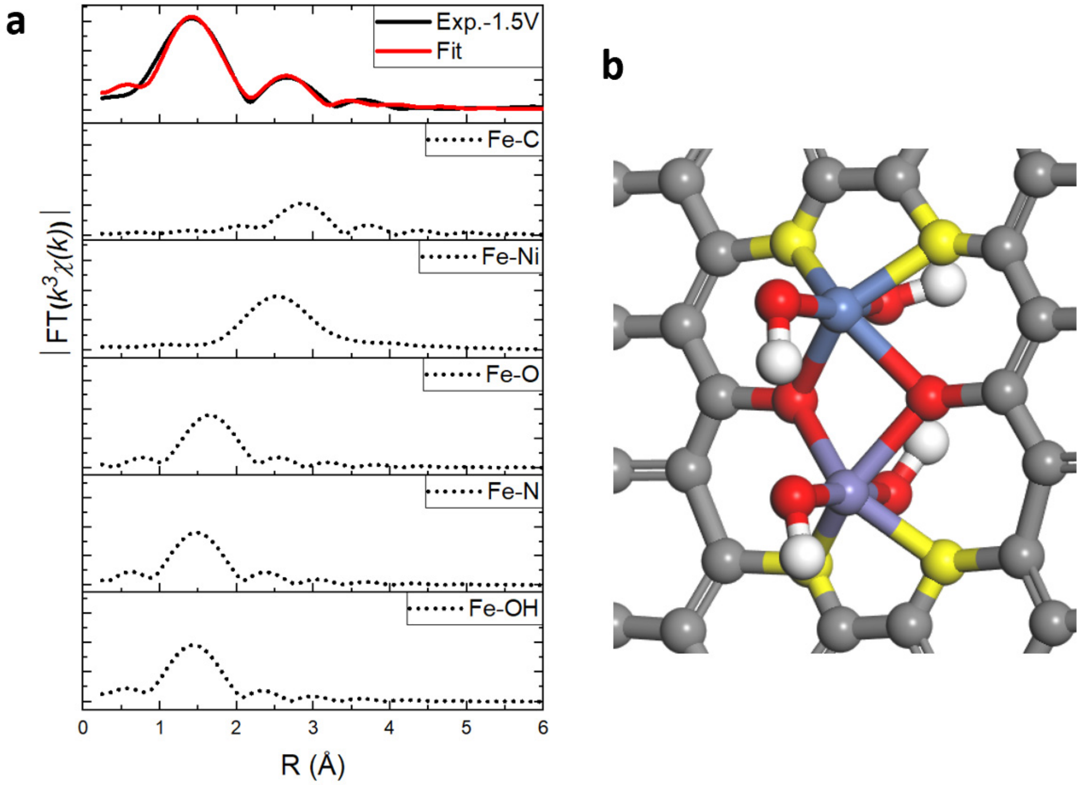

Figure S55. (a) Fitting (red) of the experimental (black) Fe K-edge EXAFS spectra $|FT(k^3\chi(k))|$ of NiFe-CNG at the potential of 1.5 V (vs. RHE) and the corresponding paths. (b) Fitting model (grey, red, yellow, white, light blue and purple spheres represent C, O, N, H, Ni and Fe, respectively).

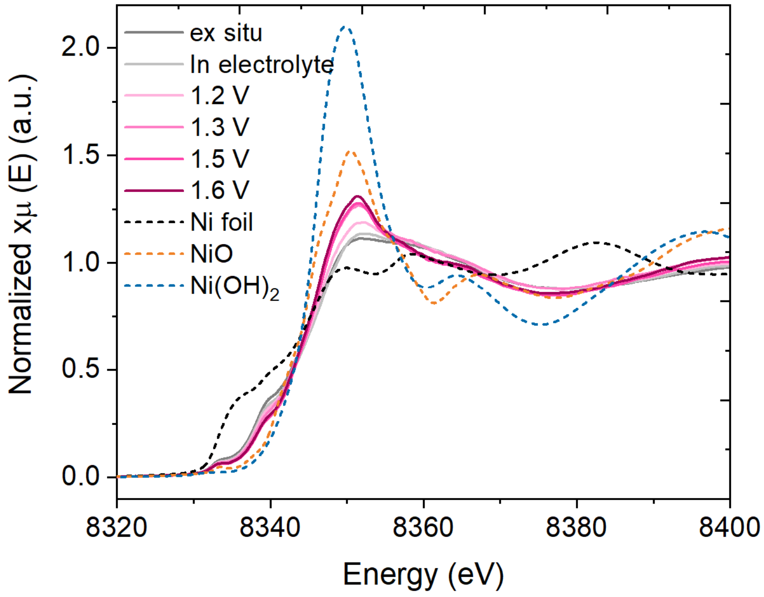

Figure S56. In situ Ni K-edge XANES spectra of Ni-CNG at different applied potentials vs. reference samples.

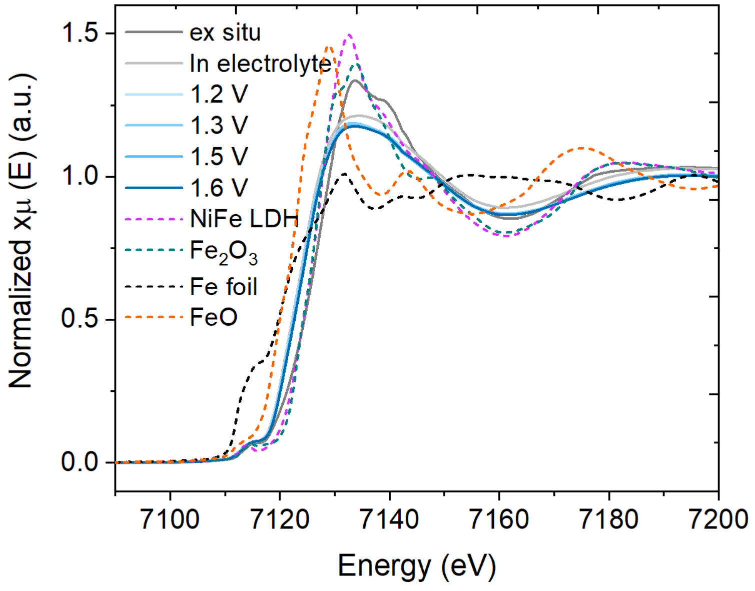

Figure S57. In situ Fe-K edge XANES spectra of Fe-CNG at different applied potentials vs. reference samples.

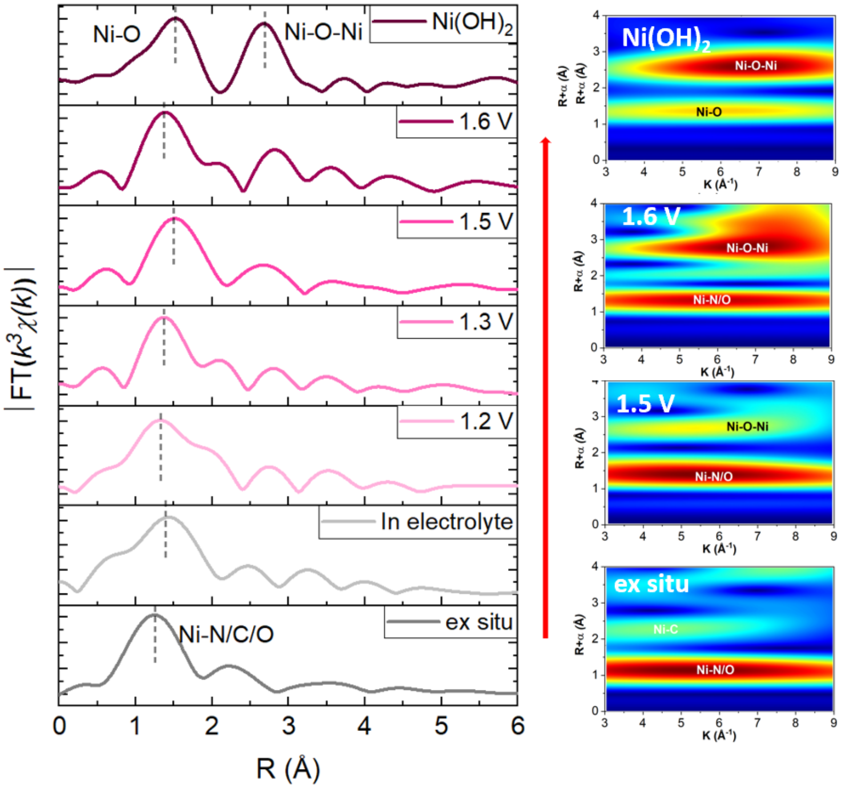

Figure S58. FT-EXAFS spectra and corresponding WT-EXAFS spectra for the k^3-weighted data of Ni-CNG at different applied potentials.

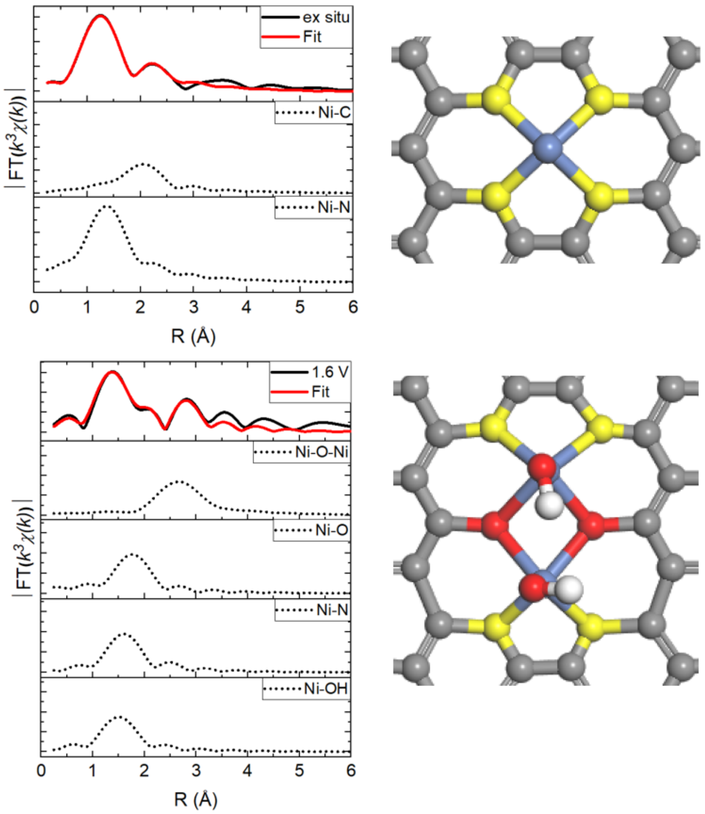

Figure S59. Fitting (red) of the experimental (black) Ni K-edge EXAFS spectra |FT($k^3\chi(k)$)| of Ni-CNG under ex situ conditions and 1.6 V vs. RHE (bottom). Right: corresponding fitting models (grey, red, yellow, white and light blue spheres represent C, O, N, H and Ni, respectively).

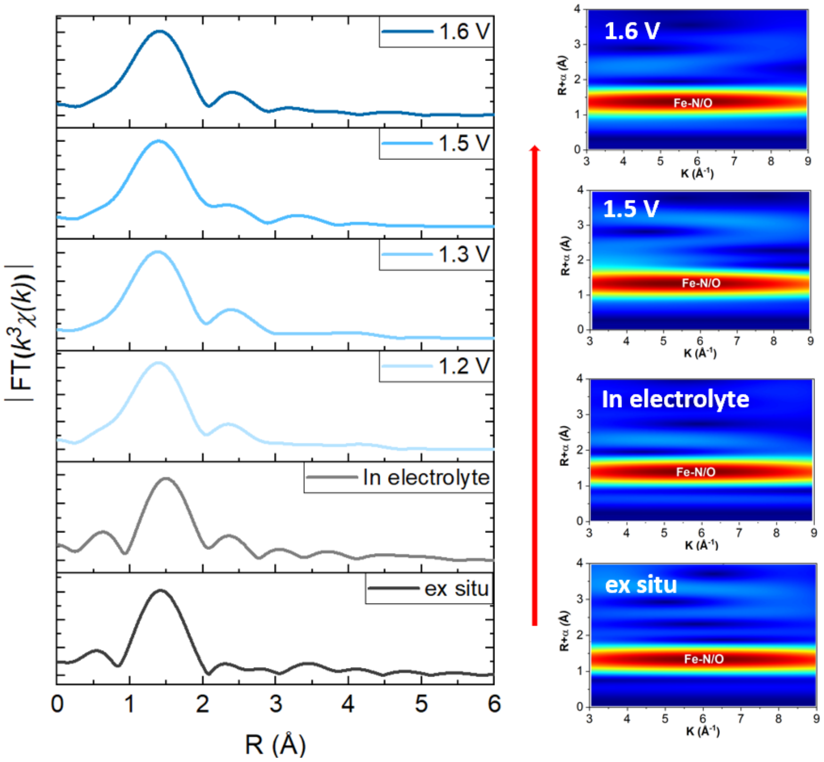

Figure S60. FT-EXAFS spectra and corresponding WT-EXAFS spectra for the k^3-weighted data of Fe-CNG at different applied potentials.

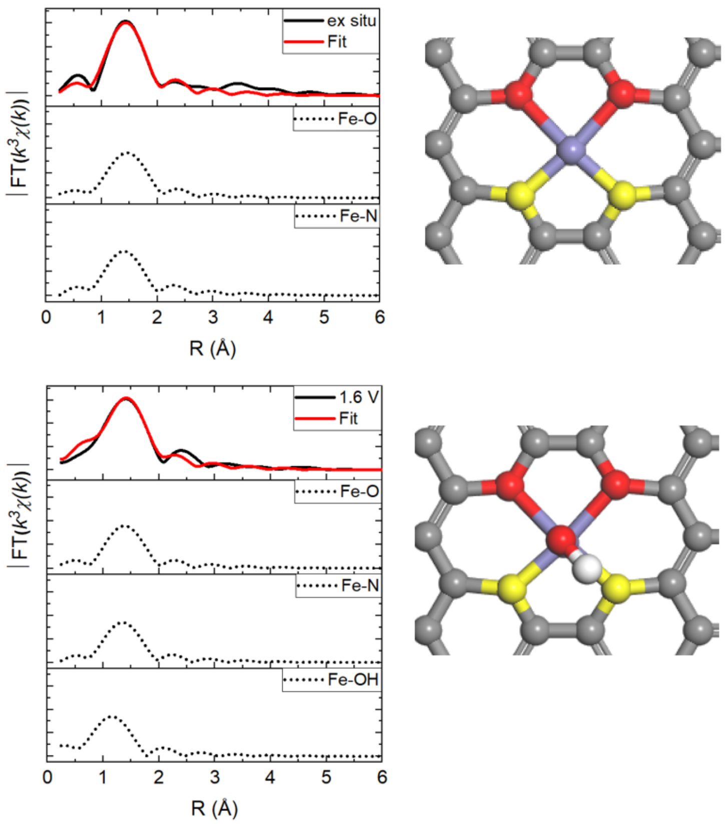

Figure S61. Fitting (red) of the experimental (black) Fe K-edge EXAFS spectra $|FT(k^3\chi(k))|$ of Fe-CNG under ex situ conditions and 1.6 V vs. RHE (bottom) with corresponding fitting models (right; grey, red, yellow, white, and purple spheres represent C, O, N, H, and Fe, respectively).

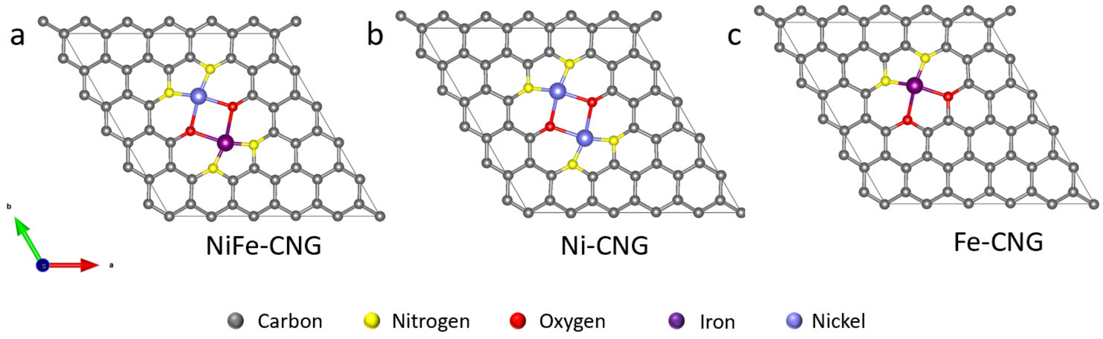

Figure S62. DFT models for the NiFe-CNG, Ni-CNG, and Fe-CNG catalysts.

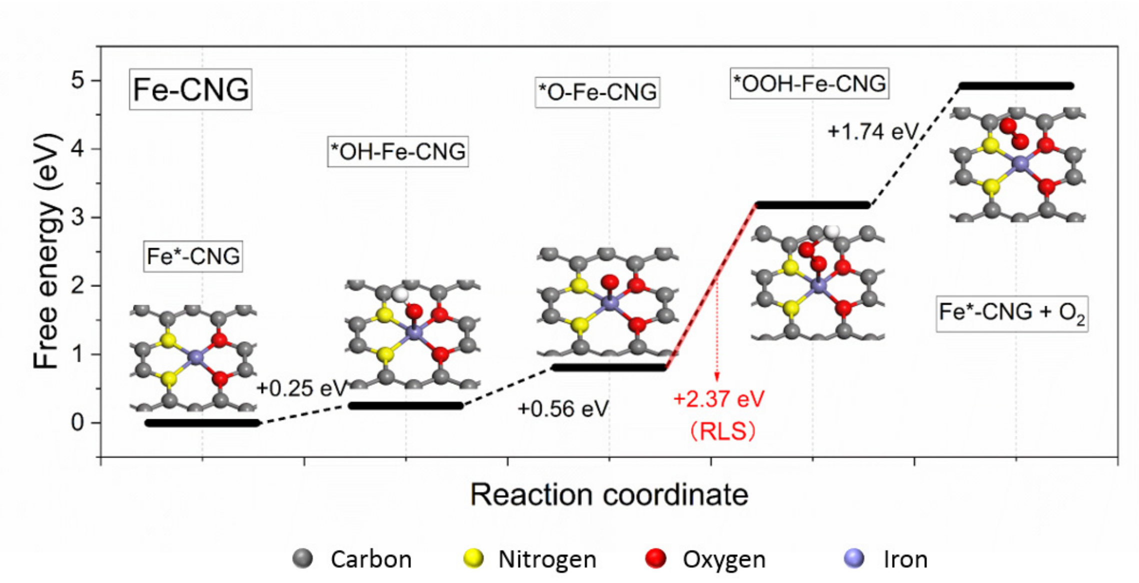

Figure S63. Free energy diagram of OER cycling at the Fe site of the Fe-CNG model.

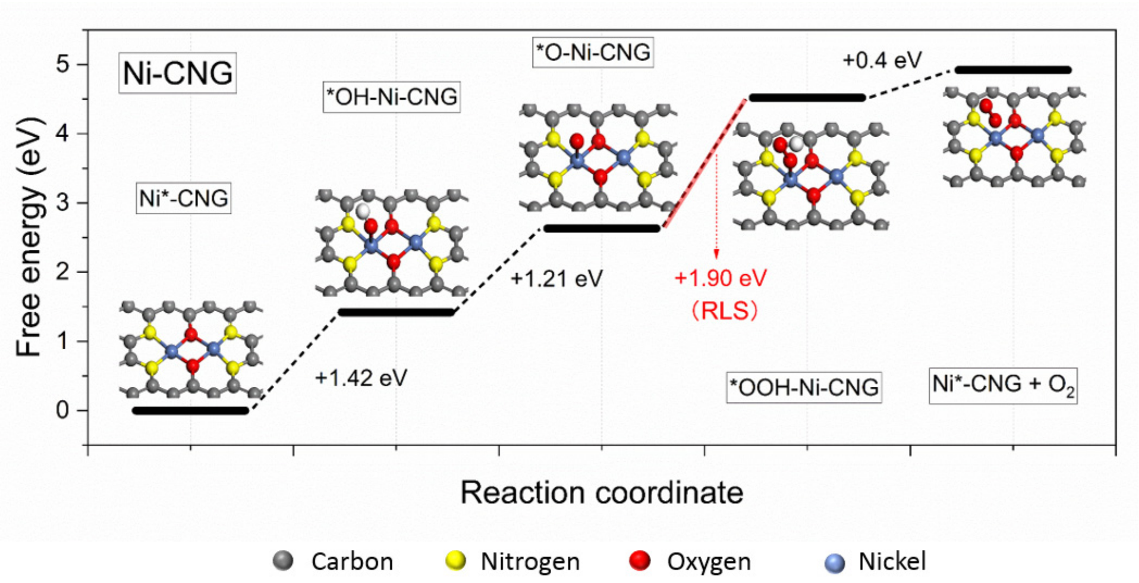

Figure S64. Free energy diagram of OER cycling at the Ni site of the Ni-CNG model.

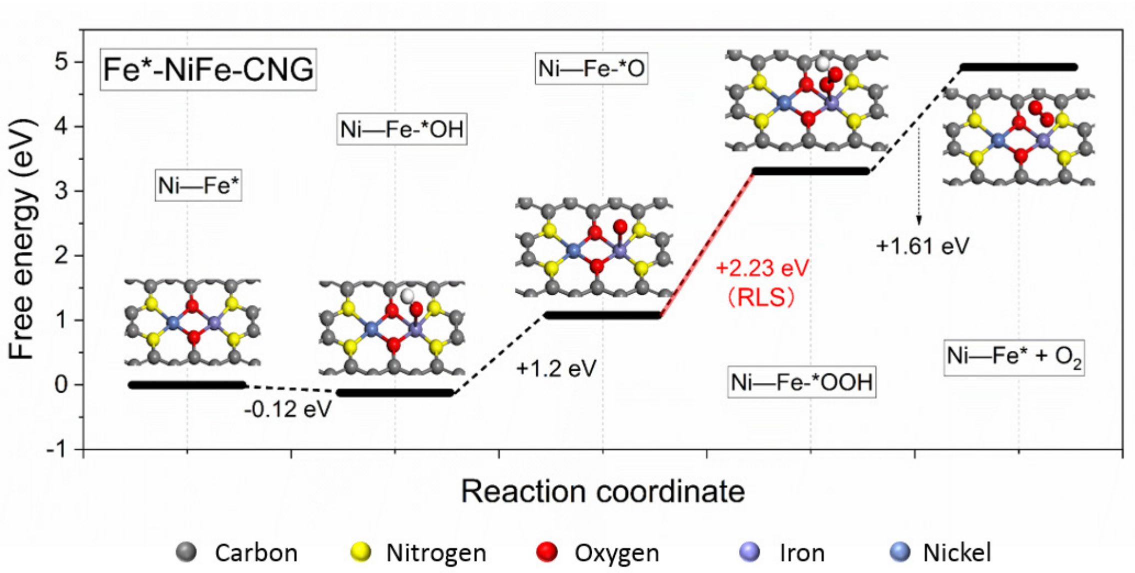

Figure S65. Free energy diagram of OER cycling at the Fe site of the NiFe-CNG model.

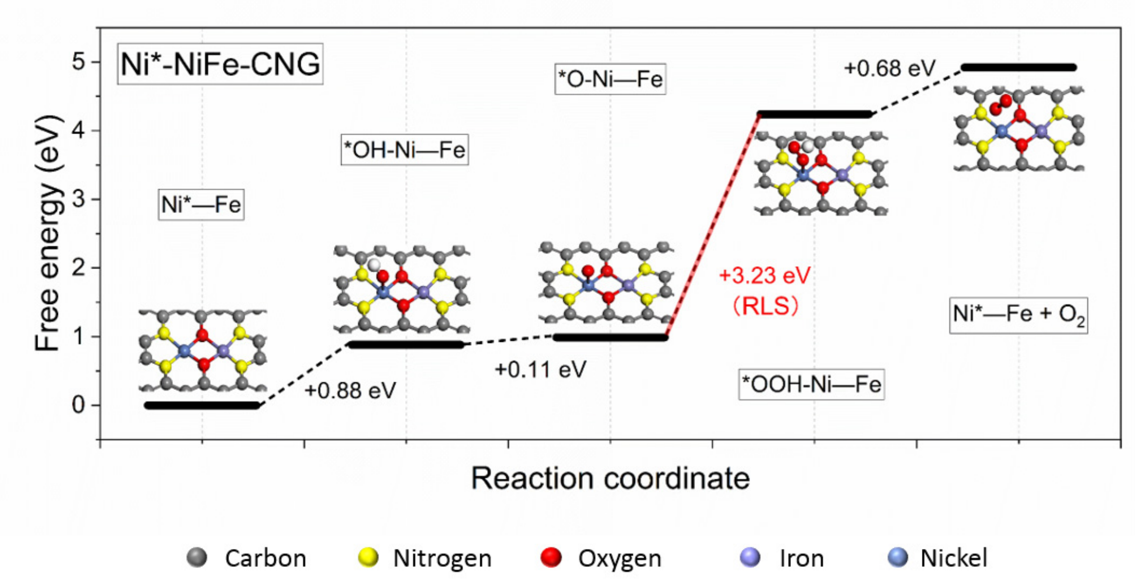

Figure S66. Free energy diagram of OER cycling at the Ni site on the NiFe-CNG model.

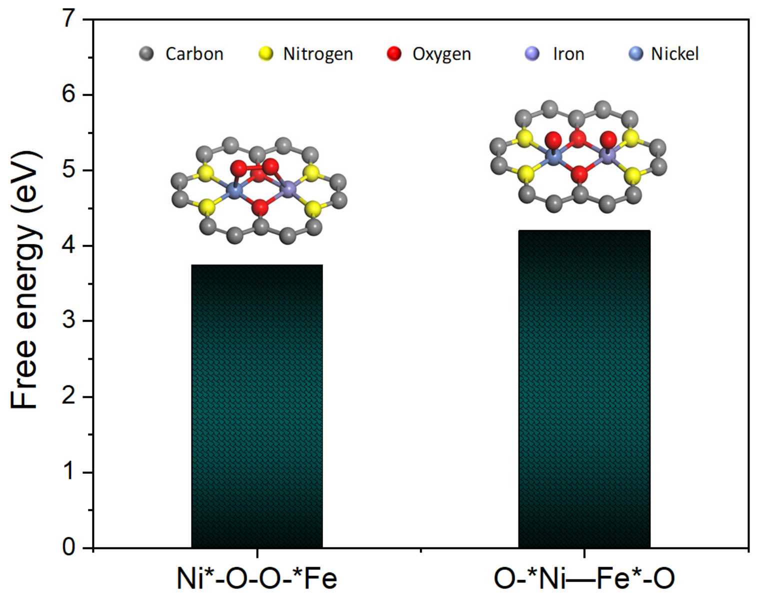

Figure S67. Free energy comparison of bridging O-O and *O on the Ni-O-Fe site in the NiFe-CNG model.

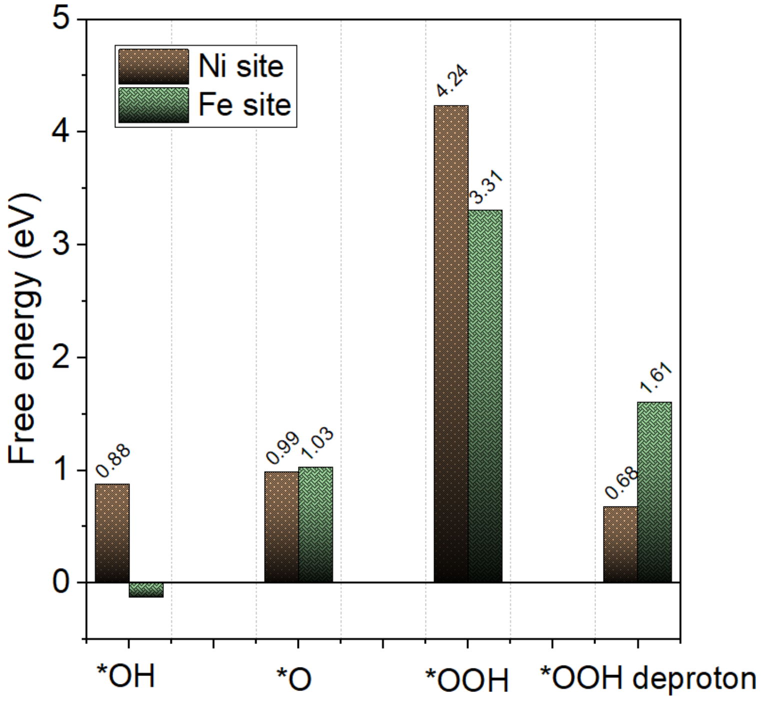

Figure S68. Free energy comparison of *OH, *O, *OOH adsorption, and *OOH deprotonation at the Ni and Fe sites of the NiFe-CNG model.

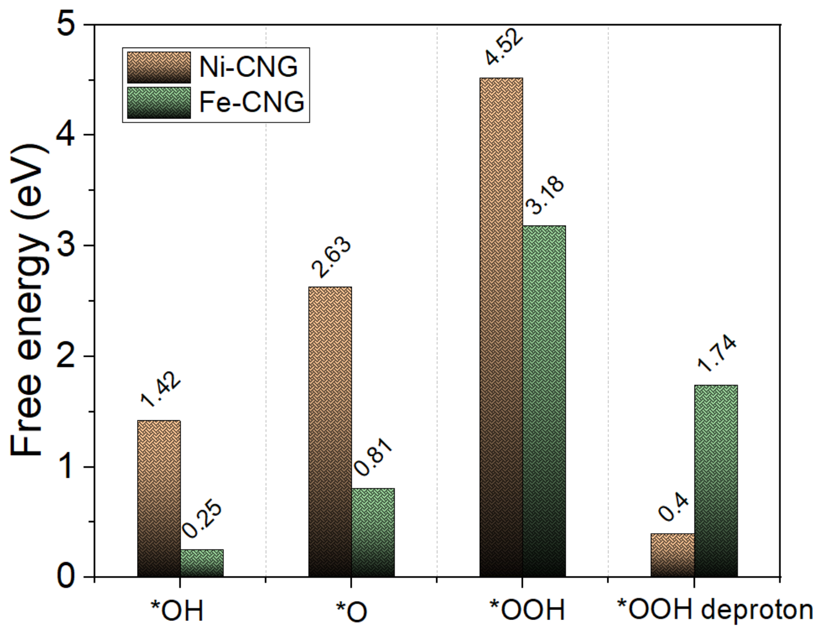

Figure S69. Free energy comparison of *OH, *O, *OOH adsorption and *OOH deprotonation at the Ni and Fe sites of the Ni-CNG and Fe-CNG models.

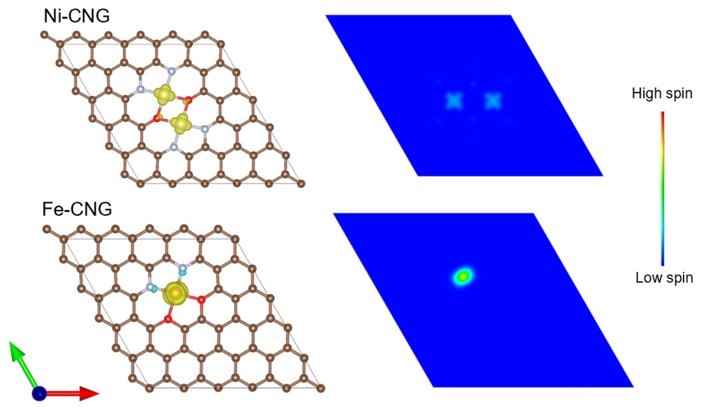

Figure S70. Spin density pattern (left) and spin channels (right) of the Ni and Fe sites in Ni-CNG and Fe-CNG. The isosurfaces in yellow and blue represent spin-up and spin-down densities, respectively.

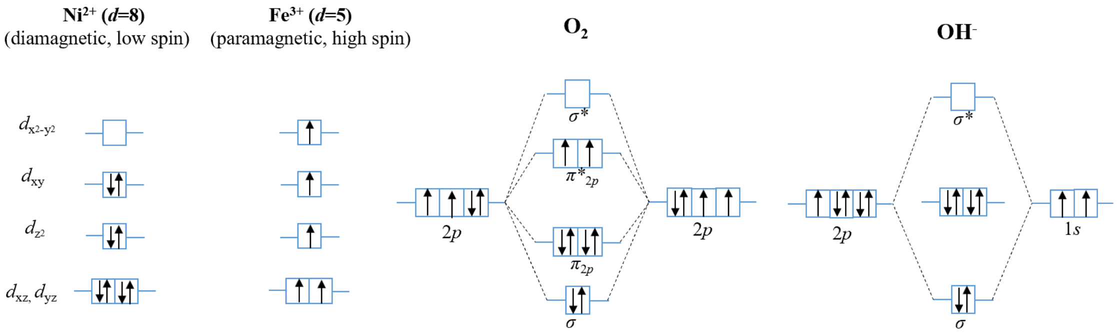

Figure S71. Diagram of of *d*-electron configurations (square planar) of iron and nickel cations in Ni-CNG, Fe-CNG and NiFe-CNG, and the MO diagrams of O_2 and OH^-.

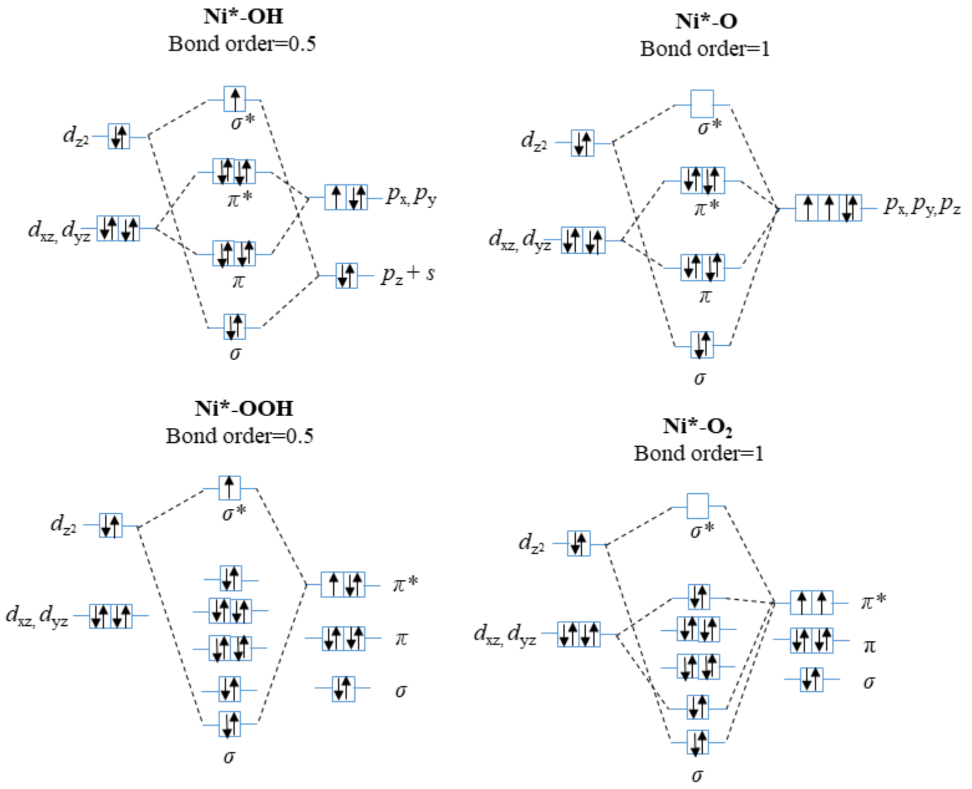

Figure S72. Diagram of the orbital interactions between Ni and *OH, *O, *OOH and O_2.

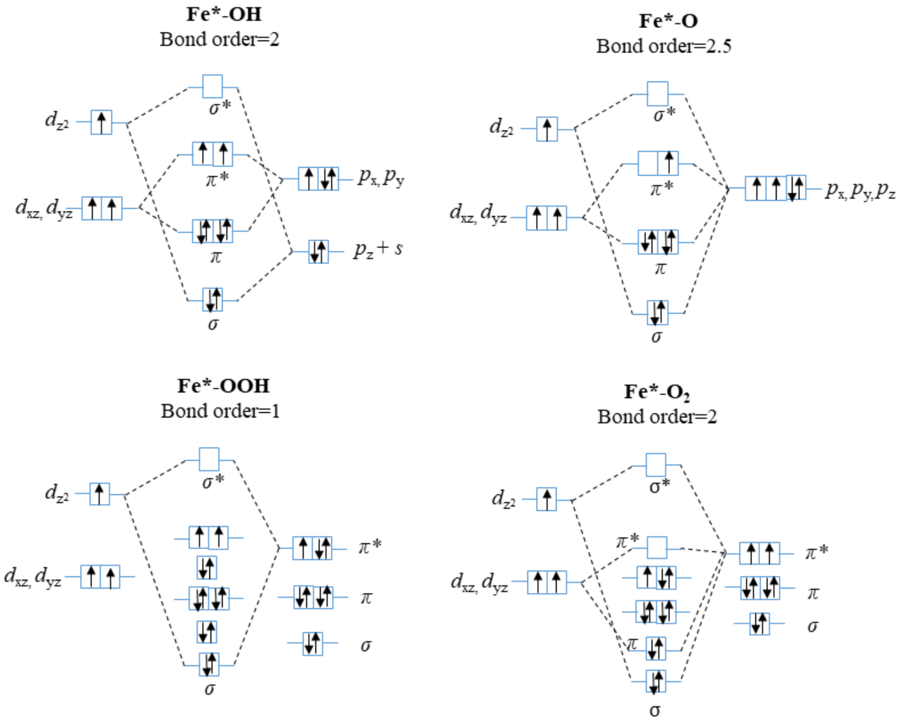

Figure S73. Diagram of the orbital interactions between Fe and *OH, *O, *OOH and O_2.

The spin distribution (Figure S71) suggests a spin channel around the Fe sites, while no such channel can be propagated through the Ni sites. This indicates that the stable spin configurations of Fe and Ni cations are in their high spin state and low spin state, respectively. Due to the square planar structures of the synthesized SACs here, the electrons in the five 3*d*-orbitals of the metal are rearranged based on the square planar configuration. The high spin state of Fe^{3+} permits each of the t_{2g} and e_g orbitals to be occupied by one unpaired electron with a single spin, thus enabling the whole *d*-shell to act as selective gate to promote the transfer of local spin currents. In principle, the low spin configuration of Ni^{2+} initially has no unpaired spin, however, the selected models are the structures after OER. The oxidation state of Ni species is slightly increased during and after OER as confirmed by both XANES and CV measurements. Therefore, the Ni atoms in the calculated models may have a slight fraction of unpaired electrons. Regarding the orbital interaction analyses, due to the symmetry conservation, the interactions between the d_{x2-y2} and d_{xy} orbitals of Ni and Fe cations and the orbitals of the adsorbed intermediates are negligible. As the catalysts exhibit square planar geometries, the intermediates are usually adsorbed at the metal centers along the *z*-axis.[17,18] Therefore, neither d_{x2-y2} nor d_{xy} orbitals are shown in the diagrams (Figure S71-73).

Table S1. Entropic contributions to free energies.

Species	TS (eV)
*OH	0
*O	0
*OOH	0
H_2	0.41
O_2	0.64
H_2O	0.58

Table S2. Zero-point energy corrections to free energies (single site).

	*OH (eV)	*O (eV)	*OOH (eV)
Ni-CNG	0.31	0.07	0.42
Fe-CNG	0.33	0.11	0.43
Ni*-Fe-CNG	0.31	0.07	0.42
Ni-Fe*-CNG	0.31	0.06	0.41

Table S3. Zero-point energy corrections to free energies (dual sites).

	ZPE (eV)
OH-*Ni-Fe*-OH	0.65
O-*Ni-Fe*-OH	0.39
OH-*Ni-Fe*-O	0.39
O-*Ni-Fe*-O	0.13
Ni*-O-O-*Fe	0.14

Table S4. Free energies of *OH, *O and *OOH adsorbed on the selected active sites (single site).

	*OH (eV)	*O (eV)	*OOH (eV)
Ni-CNG	1.42	2.63	4.52
Fe-CNG	0.25	0.81	3.18
Ni*-Fe-CNG	0.88	0.99	4.24
Ni-Fe*-CNG	-0.12	1.08	3.31

Table S5. Free energies of *OH, *O and *O-O adsorbed on the selected active sites (dual sites).

	Free energy (eV)
OH-*Ni-Fe*-OH	2.06
O-*Ni-Fe*-OH	3.44
OH-*Ni-Fe*-O	2.79
O-*Ni-Fe*-O	4.20
Ni*-O-O-*Fe	3.75

Table S6. Mass and atomic content of the metallic elements in Ni-CNG, Co-CNG, FeCNG, and NiFe-CNG measured by ICP-MS and XPS, respectively.

	Ni-CNG			Co-CNG			Fe-CNG			NiFe-CNG			
	Ni	N	O	Co	N	O	Fe	N	O	Ni	Fe	N	O
ICP-MS (wt. %)	7.5	-	-	7.6	-	-	10.5	-	-	2.2	5.3	-	-
XPS (wt. %)	5.6	14.1	5.0	6.1	12.4	6.3	6.6	9.6	6.2	3.4	2.2	13.4	5.8
XPS (at. %)	1.22	13.03	3.05	1.37	11.68	4.59	1.57	9.0	5.1	0.74	0.47	12.41	4.71

Table S7. ICP-MS measurements of the electrolytes (0.1 M KOH) before and after the long-term OER tests for NiFe-CNG.

	Ni [ng mL^{-1}]	Co [ng mL^{-1}]	Fe [ng mL^{-1}]
Fresh KOH	43	1.6	45
OER	45	1.4	43

Table S8. Main interatomic distances, atomic coordination numbers (*N*) and Debye-Waller factors (σ^2) and energy shift (ΔE_0) calculated from Artemis fitting of the experimental FT|$k^3\chi(k)$| spectra of Ni-CNG, Co-CNG, and Fe-CNG.

	Path	R (Å)	*N*	σ^2 (Å^2)
Ni-CNG-ex-situ	Ni-N	1.91	4.0	0.017
ΔE_0≈ -4.73 eV	Ni-C	2.78	4.0	0.015
R-factor=0.003	Ni-Ni	-	-	-
Ni-CNG-1.6 V	Ni-N	2.11	2.0	0.003
ΔE_0≈ 2.189 eV	Ni-O	2.27	2.0	0.004
R-factor=0.009	Ni-OH	1.97	2.0	0.004
	Ni-Ni	3.12	1.0	0.002
Co-CNG	Co-N	1.92	3.0	0.002
ΔE_0≈ -9.8 eV	Co-O	2.03	1.0	0.003
R-factor=0.009	Co-C	-	-	-
Fe-CNG-ex-situ	Fe-N	1.95	2.0	0.0043
ΔE_0≈ -4.448 eV	Fe-O	1.98	2.0	0.0040
R-factor=0.005	Fe-C	-	-	-
Fe-CNG-1.6 V	Fe-N	1.70	2.0	0.0021
ΔE_0≈ -9.80 eV	Fe-O	1.91	2.0	0.0025
R-factor=0.021	Fe-OH	1.86	2.0	0.0023

Note: The ex situ samples of Ni-CNG, Co-CNG, and Fe-CNG were fitted based on the models of Ni-N4, Co-N3-O and Fe-N2-O2 as shown in Figure S59 and Figure S61. The sample of Ni-CNG at 1.6 V was fitted based on the model of Ni-O-Ni-2OH shown in Figure S59. The sample of Fe-CNG at 1.5 V was fitted based on the model of Fe-N2-O2 with OH- adsorbed at the metal center. All the models are included in the supporting materials.

Table S9. Main interatomic distances, atomic coordination numbers (*N*) and Debye-Waller factors (σ^2) and energy shift (ΔE_0) calculated from Artemis fitting of the experimental operando FT|$k^3\chi(k)$| spectra of NiFe-CNG.

	Path	R (Å)	*N*	σ^2 (Å^2)
ex-situ	Ni-N	1.87	4.0	0.007
NiFe-CNG (Ni *K*-edge)	Ni-O	-	-	-
$\Delta E_0 \approx 2.616$ eV	Ni-C	-	-	-
R-factor=0.020	Ni-O-Ni	-	-	-
1.3 V	Ni-N	1.86	2.0	0.009
NiFe-CNG (Ni *K*-edge)	Ni-O	2.02	2.0	0.003
$\Delta E_0 \approx 6.334$ eV	Ni-OH	2.00	1.5	0.008
R-factor=0.0077	Ni-Ni	3.01	1.0	0.003
	Ni-C	3.50	4.0	0.002
1.5 V	Ni-N	1.98	2.0	0.002
NiFe-CNG (Ni *K*-edge)	Ni-O	2.11	2.0	0.051
$\Delta E_0 \approx 9.8$ eV	Ni-OH	2.04	1.5	0.006
R-factor=0.0410	Ni-Ni/Fe	3.04	1.5	0.003
	Ni-C	3.50	4.0	0.002
EX-situ	Fe-N	1.90	2.0	0.003
NiFe-CNG (Fe *K*-edge)	Fe-O	2.01	2.0	0.002
$\Delta E_0 \approx 3.891$ eV	Fe-Fe/Ni	-	-	-
R-factor=0.038				
**1.3 V**	Fe-N	1.89	2.0	0.003
**NiFe-CNG (Fe *K*-edge)**	Fe-O	1.92	2.0	0.002
$\boldsymbol{\Delta E_0 \approx -7.427}$ **eV**	Fe-OH	1.70	2.0	0.002
**R-factor=0.012**	Fe-Fe	-	-	-
**1.5 V**	Fe-N	1.98	2.0	0.00034
**NiFe-CNG (Fe *K*-edge)**	Fe-O	2.12	2.0	0.00035
$\boldsymbol{\Delta E_0 \approx 4.527}$ **eV**	Fe-OH	1.89	2.0	0.00037
**R-factor=0.005**	Fe-Fe/Ni	2.98	1.0	0.00030
	Fe-C	3.56	3.0	0.00032

Note: The ex situ samples were fitted based on the models of Ni-N4 and Fe-N2-O2 as shown in Figure S59 and Figure S61. The samples at 1.3 V were fitted based on the same models with ex situ samples but with added -OH on the metal centers. For samples at 1.5 V, the fittings were conducted based on the model proposed in Figure S54 and Figure S55**.**

Table S10. Comparison of ORR performance and stability of the as-prepared catalysts with recent representative SACs.

SAC	$E_{1/2}$ (V vs. RHE)	Limiting current at 1600 rpm (mA cm^{-2})	Stability (CV cycle)	Loading mass (mg cm^{-2})	Electrolyte	Ref.
Ni–NHGF	0.86	3.5	-	0.275 mg cm^{-2}	0.1 M KOH	[19]
Co-SAS/HOPNC	0.89	6.0	5000	0.6 mg cm^{-2}	0.1 M KOH	[20]
Co-ISAS/p-CN	0.83	5.0	5000	-	0.1 M KOH	[21]
Co@N-C	0.82	6.0	-	-	0.1 M KOH	[22]
Fe_1-HNC-500-850	0.842	5.8	5000	0.2 mg cm^{-2}	0.1 M KOH	[23]
Co−N/CNFs	0.82	5.3	10000	0.1 mg cm^{-2}	0.1 M KOH	[24]
Fe_3C@N-CNT	0.85	5.8	-	0.25 mg cm^{-2}	0.1 M KOH	[25]
Fe@Aza-PON	0.83	6.0	-	-	0.1 M KOH	[26]
Fe-N-C	0.78	5.8	10000	0.4 mg cm^{-2}	0.1 M $HClO_4$	[27]
SA-Fe-HPC	0.89	5.5	3000	0.1 mg cm^{-2}	0.1 M KOH	[28]
SA-Fe/NG	0.88	5.5	5000	0.24 mg cm^{-2}	0.1 M KOH	[29]
FeCoNx/C	0.86	5.8	5000	-	0.1 M $HClO_4$	[30]
FeNCS	0.88	5.8	-	0.5 mg cm^{-2}		[31]
Fe−N−C	0.91	5.7	5000	0.3 mg cm^{-2}	0.1 M KOH	[32]
Ni-N_4/GHSs/Fe-N_4	0.83	5.8	-	0.26 mg cm^{-2}	0.1 M KOH	[33]
Mn−N_2C_2	0.915	4.6	20000	0.102 mg cm^{-2}	0.1 M KOH	[34]
Cu-S_1N_3	0.918	5.4	5000	0.102 mg cm^{-2}	0.1 M KOH	[35]
Mo-O/N-C	0.85	5.0	5000	0.204 mg cm^{-2}	0.1 M KOH	[36]
FePc-GO	0.89	5.2	5000	0.2 mg cm^{-2}	0.1 M KOH	[37]
Fe-CNG	0.89	6.0	20000	0.2 mg cm^{-2}	0.1 M KOH	**This work**
CoFe-CNG	0.85	5.6	-	0.2 mg cm^{-2}	0.1 M KOH	**This work**
NiFe-CNG	0.82	5.3	-	0.2 mg cm^{-2}	0.1 M KOH	**This work**
Co-CNG	0.84	5.6	-	0.2 mg cm^{-2}	0.1 M KOH	**This work**

Table S11. Comparison of OER performance and stability of the as-prepared catalysts with recent representative SACs.

SACs	Overpotential (mV) at 10 mA cm^{-2}	Tafel slope (mV dec^{-1})	Stability (h)	Loading mass (mg cm^{-2})	Refs.
S|NiNx−PC/EG	280	45	10 h	0.15 mg cm^{-2}	38
$NiSO_4$-GF	300	80	20 h		39
CoFe dual site SAC	309	58	16 h	-	40
NiPc-GO	320	61	17 h	0.1 mg cm^{-2}	37
NiN_4C_4	331	63	20 h	0.275 mg cm^{-2}	19
Mn-NG	337	55	120 h	-	41
$Mn{-}N_2C_2$	350	75.6	22 h	0.102 mg cm^{-2}	34
S,N-Fe/N/C-CNT	370	82	-	0.6 mg cm^{-2}	42
FeCo-Nx-CN	370	57	0.84 h	0.1 mg cm^{-2}	43
$Co{-}C_3N_4$/CNT	380	82	-	2.0 mg cm^{-2}	44
Co@NG	386	73	20 h	0.28 mg cm^{-2}	45
Ni-N_4/GHSs/Fe-N_4	390	81	2.8 h	0.26 mg cm^{-2}	33
NiFe-CNG	270	69	72 h	0.2 mg cm^{-2}	**This work**
Ni_8Fe_2-CNG	310	109	-	0.2 mg cm^{-2}	**This work**
Ni_6Fe_4-CNG	340	62	-	0.2 mg cm^{-2}	**This work**
Ni_5Fe_5-CNG	350	61	-	0.2 mg cm^{-2}	**This work**

Table S12. Comparison of Zn-air battery performance of the as-prepared catalysts with recent representative SACs.

Catalysts	Maximum power density (mW cm^{-2})	Refs.
Mn-N_2C_2	258	[34]
CoSAs-NGST	247	[46]
3DOM Fe-N-C	235	[47]
Fe-N_4 SAs/NPC	232	[48]
Fe-NSDC	225.1	[49]
S-Cu-ISA/SNC	225	[35]
Mn-SAS/CN	220	[50]
FeCu-N-HC	209.4	[51]
Fe/N-CNRs	181.8	[52]
CoSA/N,S-HCS	173.1	[53]
SACe-N/PC	155.0	[54]
SA-FeCo-NC	148.2	[55]
Fe/N-G-SAC	120	[56]
Fe-CNG	239.1	**This work**
NiFe-CNG	185.9	**This work**
20 wt.% Pt/C	152.1	**This work**

Supplementary Note 1 | Detailed discussion of XPS results regarding the determination of the coordination environments.

To provide insights into the coordination environment of the metal species, N 1*s* and O 1*s* spectra were compared with those of the metal-free samples (CNG). The binding energy intervals highlighted in Figure S31 indicate the expected ranges of the peaks associated with nitrogen or oxygen atoms bonded to metal ions. Details on how these ranges were defined are reported in the following.

N1*s* region. The range reported in Figure S32a - 398.7 to 399.3 eV - was defined by considering the results presented in XPS studies on the interaction between transition metals and N-donor ligand-containing compounds, such as porphyrins and phthalocyanines,[57] or N-doped graphitic materials.[58,59]

Clear insights on the variation of the chemical state of nitrogen upon addition of iron, cobalt or nickel can be readily obtained by the comparison of the N1*s* spectra for metal-containing and metal-free samples. All the spectra of metal-containing samples show a variation of the relative intensity of the two main peaks and a concurrent increase of intensity in the valley between the two peaks. Notably, this increase of intensity is mostly localized in the BE interval expected for nitrogen bonded to metals. It is also worth noting that the M-N (M=Ni, Co, and Fe) signal appears to grow at the expenses of the high-BE peak, and that it partially overlaps with the low-BE peak. This is qualitatively analogous to what has been observed in the direct synthesis of metalloporphyrin complexes, which also supports the assignment of the two main peaks to pyrrole- and pyridine-like nitrogen.[57,60]

O1*s* region. The O 1*s* spectra of metal-containing samples as depicted in Figure S32b were analyzed based on previous O1*s* spectra recorded on transition metal oxides, hydroxides and oxyhydroxides, and on carbonates as well as hydrogencarbonates of both transition metals and alkali metals. Overall, two ranges of binding energy are defined, namely metal bonded to oxygen in interstitial lattice sites of oxides from 529.6 to 530.0 eV, and the metal bonded to oxygen-containing species from 531.0 to 531.8 eV.[61]

Fe, Co, Ni 2*p* and 3*p* regions. 3*p* signals are broader and less intense than the 2*p* signals. For this reason, they are not typically discussed in the literature. On the other hand, the severe overlap of the Ni LMM Auger signal with Fe 2*p* in NiFe-CNG (Figure S74) significantly complicates the chemical state analysis of the 2*p* region in this sample.

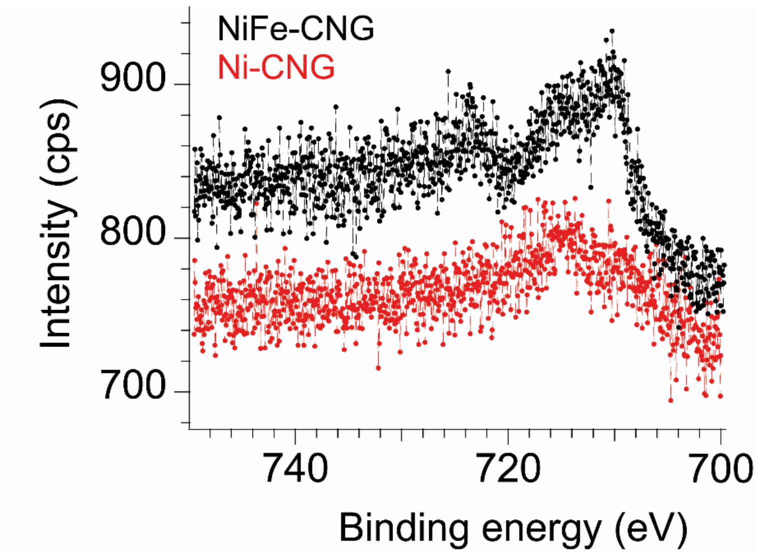

Figure S74. Ni LMN Auger signal of Ni-CNG (red) and Fe 2*p* signal (black) of NiFe-CNG.

Another overlap is also observed in the case of Co-CNG, between the photoelectron 2*p* and the Auger LMM signals (Figure S30 and S31), though in this case the effect is less pronounced. In contrast, no overlap of signals is observed in the 3*p* spectral region, therefore, the comparison of 3*p* signals can be effectively used for the analysis of chemical states, as well as for the quantitative analysis. Based on the above discussion, we recorded both 2*p* and 3*p* high-resolution spectra of Fe, Co, and Ni signals from Fe-CNG, Co-CNG, Ni-CNG, and NiFe-CNG as presented in Figure 30b.

It is worth comparing the Fe 2*p* and Co 2*p* signals with the corresponding signals from model complexes exhibiting a square planar geometry and only N-donor ligands, such as metallated porphyrins or phthalocyanines. A binding energy of 709.0 eV has been reported for the Fe^{2+} $2p_{3/2}$ signal in phthalocyanine-coordinated iron,[57] which is significantly lower than the BE of the Fe $2p_{3/2}$ signal measured for Fe-CNG (710.8 eV). This large variation clearly indicates that the chemical state of iron in Fe-CNG differs from that of the model compounds. In line with evidence collected from the analysis of the N1*s* and O1*s* spectral regions, the high binding energy value is likely related to the coordination of the metal by O-donor ligands. Similarly, a binding energy of 780.0 eV has been reported for the Co^{2+} $2p_{3/2}$ signal of phorphyrin-coordinated cobalt,[60] which is somewhat lower than the BE (781.0 eV) of Co $2p_{3/2}$ from Co-CNG. This finding suggests that the Co species in Co-CNG are not bonded solely to N-donor ligands. Unfortunately, drawing a detailed conclusion on the coordination geometries, on the numbers and types of ligand, and on oxidation states of the transition metal only from XPS analyses of Fe-CNG and Co-CNG is not as straightforward as in the case of Ni-CNG. The conclusions presented in the manuscript were made by integrating this information with XAS analyses.

The shape and position on the binding energy scale of the Fe 3*p* and Ni 3*p* signals of NiFe-CNG are indistinguishable from those of the corresponding signals of Ni-CNG and Fe-CNG (Figure S75). This

suggests that the chemical states of the metals in the mixed sample are very similar to those of the samples containing a single metal.

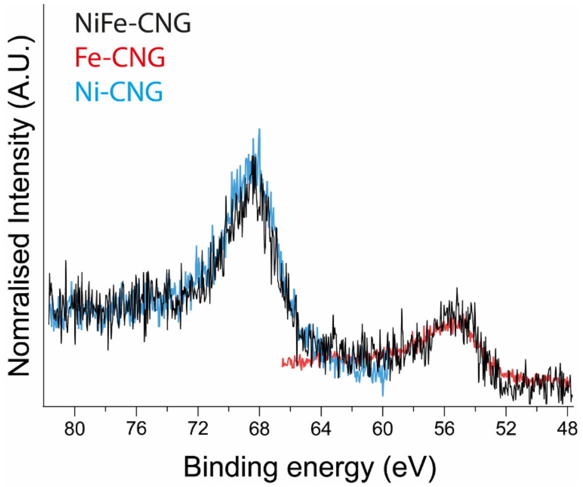

Figure S75. Ni and Fe 3*p* signals of NiFe-CNG compared with Ni-CNG and Fe-CNG.

Supplementary Note 2 | Exclusion of the formation of NiFe oxyhydroxide clusters.

To exclude the formation of NiFe oxyhydroxide, we further compared the Ni K-edge k^3-weighted EXAFS spectrum of the post catalytic NiFe-CNG sample under 1.5 V vs. RHE with those of $Ni(OH)_2$ and NiOOH reported in previous works.[62] The oscillations in the K-space are the direct reflection of the local structure around the photoabsorbing atoms. As shown in Supplementary Figure S76 the EXAFS signal of NiFe-CNG in the K-space after OER at the potential of 1.5 V vs. RHE is completely different from those of $Ni(OH)_2$ and NiOOH, respectively, thus suggesting a different local structural environment around the metal centers in NiFe-CNG.

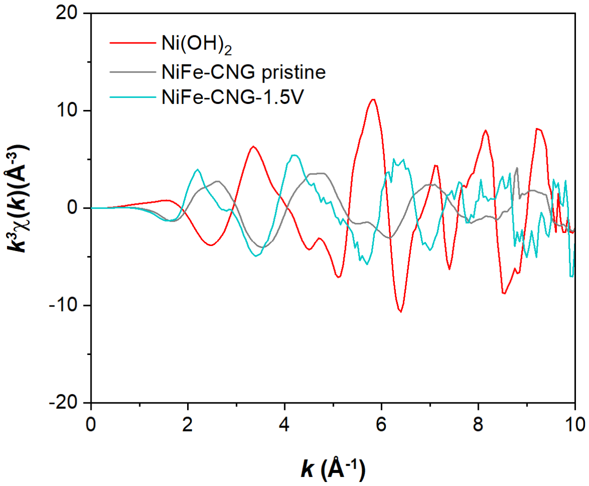

Figure S76. Ni K-edge k^3-weighted EXAFS oscillations of pristine NiFe-CNG, NiFe-CNG under 1.5 V vs. RHE vs. $Ni(OH)_2$.

To further support our conclusion, we newly recorded TEM images of NiFe-CNG and Ni-CNG at different positions after OER (Figure S77-78). The TEM images of the post-catalytic samples are very similar to the samples before OER (Figures S5-8). The dark and blurry areas in the TEM images are the overlapping graphene layers or areas covered with Nafion solution. No apparent nanoparticles or oxyhydroxide clusters were observed. We further conducted HAADF-STEM measurements for the NiFe-CNG sample after OER for 3 hours at a current density of 10 mA cm^{-2} (Figure S79). From those new HAADF-STEM images it is very clear that the metal species are still atomically dispersed on the carbon support. No obvious NPs or clusters were observed, and the macroscopic measurements suggest that no apparent oxyhydroxide clusters were formed on the surface of carbon layers after OER.

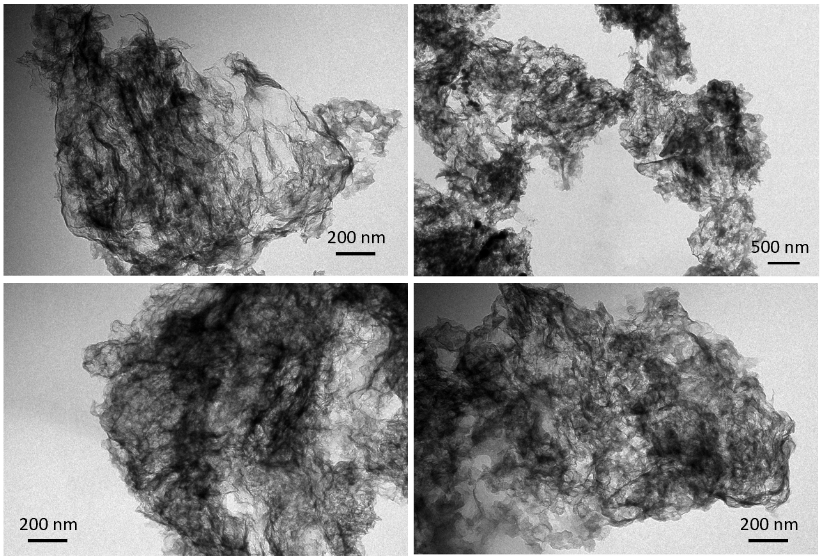

Figure S77. TEM images of NiFe-CNG after the OER for 3 hours at a current density of 10 mA cm^{-2}.

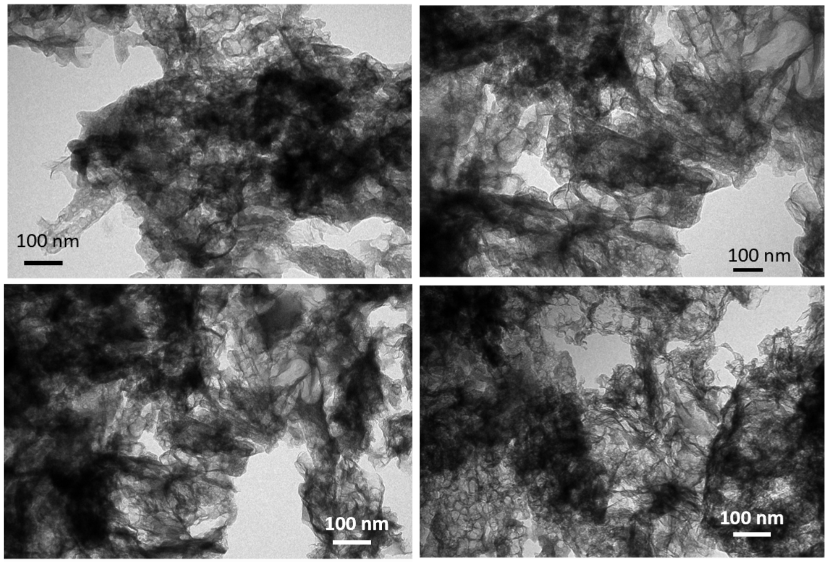

Figure S78. TEM images of Ni-CNG after 1000 CV cycles at the potential range of 0-1.7 V vs. RHE. **Note**: Ni-CNG is chemically inactive toward OER, therefore we were not able to measure it at a current density of 10 mA cm^{-2}.

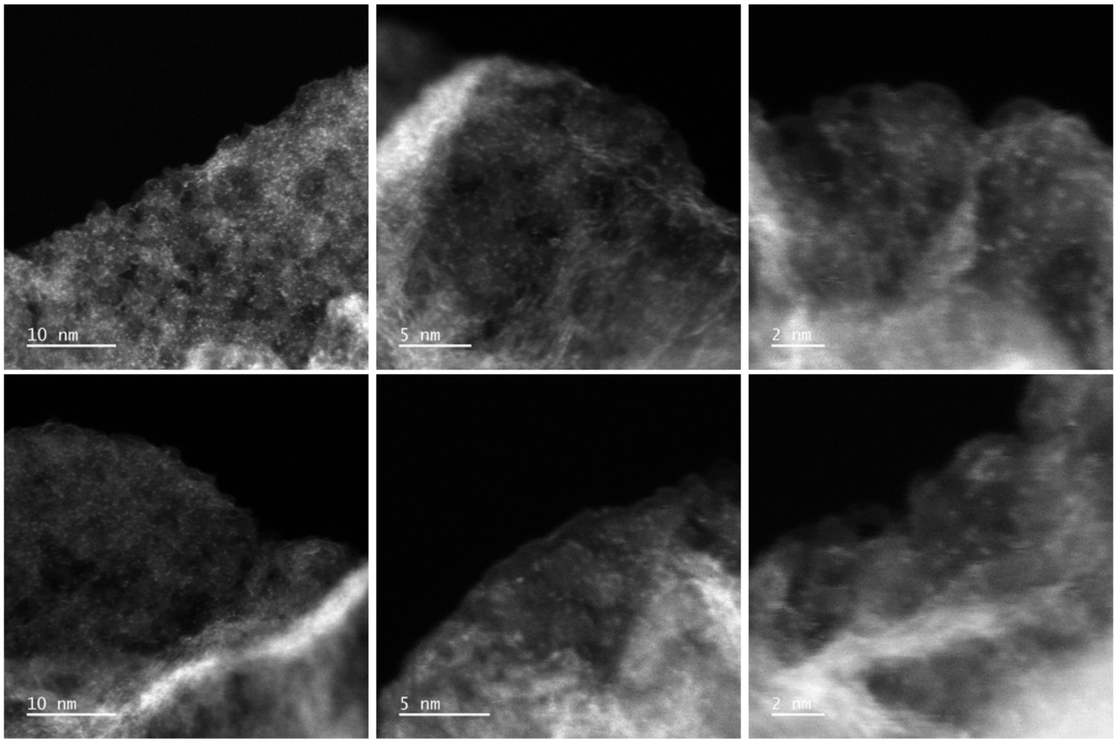

Figure S79. HAADF-STEM measurement of NiFe-CNG after OER for 3 hours at a current density of 10 mA cm^{-2}. **Note:** Due to the influence of Nafion on the surface, the contrast of the images is not as clear as in the fresh sample. However, the bright dots arising from Ni and Fe metal atoms are clearly visible.

We also recorded the Raman spectra of the NiFe-CNG and Ni-CNG samples after OER (Figure S80) and compared them with the spectra of commercial $Ni(OH)_2$ and NiOOH reported in previous works.[63,64] Both the spectra of $Ni(OH)_2$ and of NiOOH show the typical signals of Ni-O around 300-700 cm^{-1}, which were not detected in our NiFe-CNG and Ni-CNG samples. All in all, the above discussion and analyses suggest that the formation of oxyhydroxide species did not take place in our case.

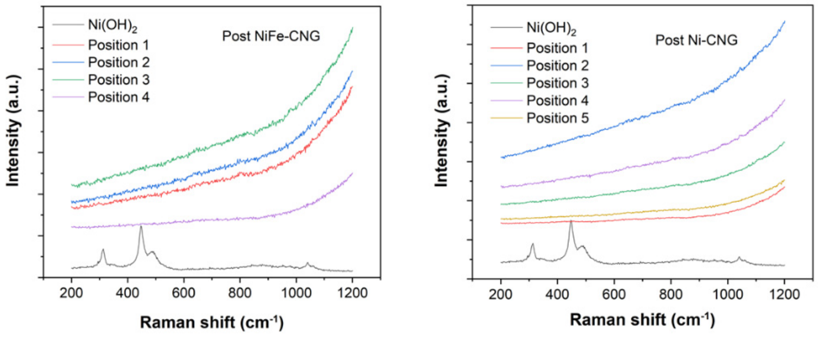

Figure S80. Raman spectra of post-catalytic NiFe-NG, Ni-CNG and a commercial $Ni(OH)_2$ reference.

To further confirm the presence of Ni-O-Fe moieties within the carbon layers, we recorded the N 1*s* and O1*s* XP spectra of a representative post-catalytic NiFe-CNG sample (Figure S81). Comparison with the metal-free CNG reference shows that the binding energy in the N 1*s* spectrum of the post-catalytic NiFe-CNG is slightly shifted to higher energy, suggesting the presence of M-N bonds (~399 eV). The changes in the O 1s spectrum around 531 eV also indicate the formation of M-O bonds in the sample. The content of Nafion in the post-catalytic NiFe-CNG sample gives rise to some additional O signals. Importantly, the presence of both M-N and M-O bonds clearly shows that the metal species are not only coordinated to O atoms but also to N atoms from the carbon layers. These results strongly support our conclusion that the Ni-O-Fe moieties (at least their majority) are located within the carbon lattice.

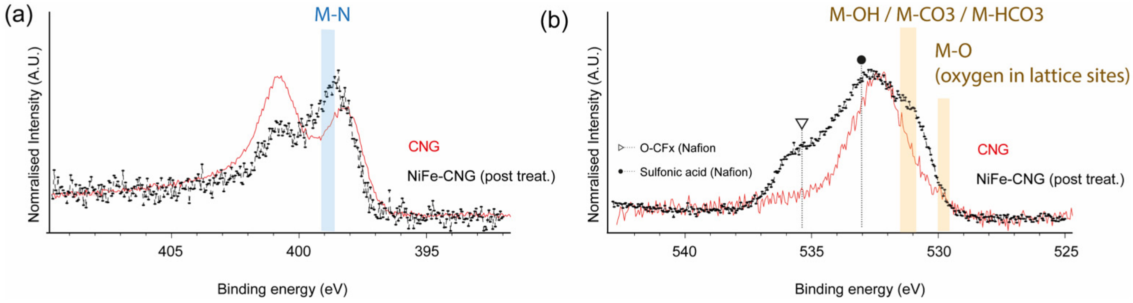

Figure S81. High-resolution XP spectra of CNG and post-catalytic NiFe-CNG: (a) N 1*s* spectra and (b) O 1*s* spectra.

It is worth noting that the structure of carbon-supported SACs is not based on perfect carbon layers. Single atoms are usually coordinated at sites with defects or edges arising from unsaturated bonds of C/N atoms (Figure S82). In such areas, some metal atoms are not perfectly coordinated with four non-metal atoms. These unsaturated bonds of metal atoms are more likely to move around the edges, which has been reported in previous works on Au single atoms[65] and Cu atoms[66] at graphene edges. Moreover, previous works showed that single atoms and metal pairs in the graphene plane can also move around within the carbon lattice via the defects/vacancies on the graphene layers.[67,68] As is evident from Figure S82, a considerable fraction of metal atoms in our catalyst is located at the defects or edges. Therefore, it is reasonable to assume that these atoms are mobile under applied potentials during OER. While a four-fold coordination of the metal atoms was derived from EXAFS fitting, we have to keep in mind that XAS is generally a bulk method that provides the average coordination numbers for the entire material.

Based on the above discussion, we propose three possible pathways for the structural reconstruction in our catalysts according to the observed characteristics of our materials:

(1) First, the edges and defective areas in our materials can provide the required space for the movement of the metal atoms during the OER. The unsaturated metal atoms can then be partially coordinated by OH^- species from the electrolyte, and further connect to the nearest neighboring Ni/Fe sites, given that

many of them are located not far away. Noteworthy, we did not conclude in our study that all of the single atoms in the NiFe-CNG were engaging in the formation of Ni-O-Ni/Fe bonds, because some separate metal atoms with a larger distance from their neighboring atoms or single atoms in the plane of the carbon lattice with perfect four-fold N/C coordination may not be able to form these M-O-M species. However, once the M-O-M species are present, their signal appears clearly in the EXAFS spectrum.

(2) Although the metal atoms are atomically dispersed within the carbon layers prior to OER, the distances between two metal atoms are not very large in many areas as can be seen from the STEM images (Figure S82), i.e. many of them are in fact within a 3 Å range. In this case, it is plausible that the metal atoms first move around and then form the M-O-M bonds with their neighboring atoms. The exposure to OH^- enriched electrolyte of such atom pairs near defects facilitates their bonding to OH^- species while they are only partially connected to the carbon support.

(3) Third, the catalysts are not consisting of single carbon layers and are in fact connected by a lot of small carbon fragments as visible from the STEM images (Figure S82). These random carbon fragments should be able to move around under the applied OER voltage. This movement of the carbon fragments, especially when resulting in contact between two edges, may also lead to the formation of M-O-M bonds in the presence of OH^- species in the electrolyte.

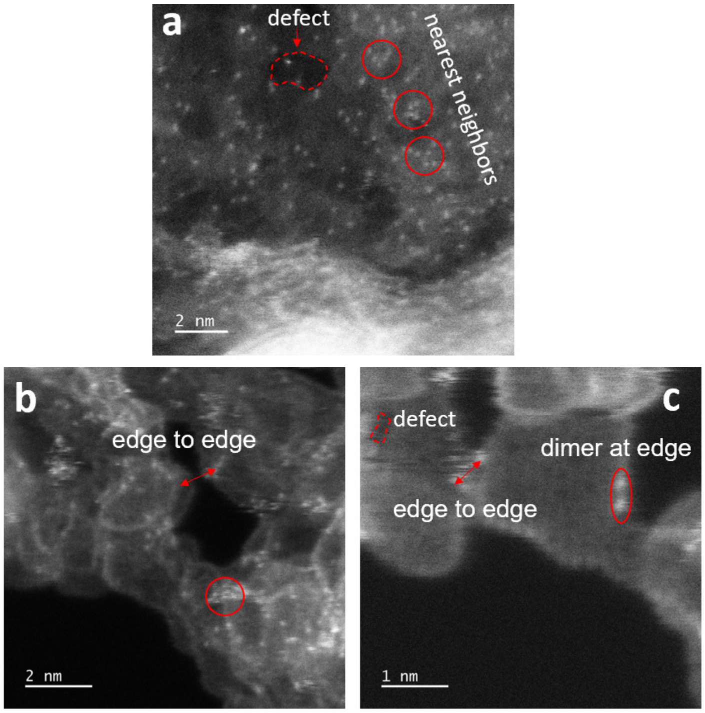

Figure S82. Representative HAADF-STEM images of a fresh NiFe-CNG sample.

References

1. Seah, M., Gilmore, I. & Spencer, S. Quantitative XPS: I. Analysis of X-ray photoelectron intensities from elemental data in a digital photoelectron database. *J. Electron. Spectrosc. Relat. Phenom.* **120,** 93–111 (2001).

2. Ayiania, M. *et al.* Deconvoluting the XPS spectra for nitrogen-doped chars: An analysis from first principles. *Carbon* **162,** 528–544 (2020).

3. Ravel, B. & Newville, M. ATHENA, ARTEMIS, HEPHAESTUS: data analysis for X-ray absorption spectroscopy using IFEFFIT. *J. Synchrotron Radiat.* **12,** 537–541 (2005).

4. Ankudinov, A. L., Ravel, B., Rehr, J. J. & Conradson, S. D. Real-space multiple-scattering calculation and interpretation of x-ray-absorption near-edge structure. *Phys. Rev. B* **58,** 7565–7576 (1998).

5. Funke, H., Chukalina, M. & Scheinost, A. C. A new FEFF-based wavelet for EXAFS data analysis. *J. Synchrotron Radiat.* **14,** 426–432 (2007).

6. Funke, H., Scheinost, A. C. & Chukalina, M. Wavelet analysis of extended x-ray absorption fine structure data. *Phys. Rev. B* **71,** 94110 (2005).

7. Muñoz, M., Argoul, P. & Farges, F. Continuous Cauchy wavelet transform analyses of EXAFS spectra: A qualitative approach. *American Mineralogist* **88,** 694–700 (2003).

8. Kresse, G. & Furthmüller, J. Efficient iterative schemes for ab initio total-energy calculations using a plane-wave basis set. *Phys. Rev. B* **54,** 11169–11186 (1996).

9. Kresse G. & Hafner, J. Ab initio molecular-dynamics simulation of the liquid-metal-amorphous-semiconductor transition in germanium. *Phys. Rev. B* **49,** 14251–14269 (1994).

10. Perdew, J. P., Burke, K. & Ernzerhof, M. Generalized gradient approximation made simple. *Phys. Rev. Lett.* **77,** 3865–3868 (1996).

11. Grimme, S. Semiempirical GGA-type density functional constructed with a long-range dispersion correction. *J. Comput. Chem.* **27,** 1787–1799 (2006).

12. Nørskov, J. K. *et al.* Origin of the overpotential for oxygen reduction at a fuel-cell cathode. *J. Phys. Chem. B* **108,** 17886–17892 (2004).

13. Valdés, Á., Qu, Z.-W., Kroes, G.-J., Rossmeisl, J. & Nørskov, J. K. Oxidation and photo-oxidation of water on TiO_2 surface. *J. Phys. Chem. C* **112,** 9872–9879 (2008).

14. Zhang, N. *et al.* Lattice oxygen activation enabled by high-valence metal sites for enhanced water oxidation. *Nat. Commun.* **11,** 4066 (2020).

15. Lyons, M. E. & Brandon, M. P. The significance of electrochemical impedance spectra recorded during active oxygen evolution for oxide covered Ni, Co and Fe electrodes in alkaline solution. *J. Electroanal. Chem.* **631,** 62–70 (2009).

16. Wang, H.-Y. *et al.* In operando identification of geometrical-site-dependent water oxidation activity of spinel Co_3O_4. *J. Am. Chem. Soc.* **138,** 36–39 (2016).

17. Li, X.-X., Cho, K.-B. & Nam, W. A theoretical investigation into the first-row transition metal–O_2 adducts. *Inorg. Chem. Front.* **6,** 2071–2081 (2019).

18. Bockris, J. O. & Otagawa, T. The electrocatalysis of oxygen evolution on perovskites. *J. Electrochem. Soc.* **131,** 290–302 (1984).

19. Fei, H. *et al.* General synthesis and definitive structural identification of MN_4C_4 single-atom catalysts with tunable electrocatalytic activities. *Nat. Catal.* **1,** 63–72 (2018).

20. Sun, T. *et al.* Single-atomic cobalt sites embedded in hierarchically ordered porous nitrogen-doped carbon as a superior bifunctional electrocatalyst. *Proc. Natl. Acad. Sci. U. S. A.* **115,** 12692–12697 (2018).

21. Han, A. *et al.* A polymer encapsulation strategy to synthesize porous nitrogen-doped carbon-nanosphere-supported metal isolated-single-atomic-site catalysts. *Adv. Mater.* **30,** 1706508 (2018).

22. Zhang, M., Dai, Q., Zheng, H., Chen, M. & Dai, L. Novel MOF-derived Co@N-C bifunctional catalysts for highly efficient Zn-air batteries and water splitting. *Adv. Mater.* **30,** 1705431; 10.1002/adma.201705431 (2018).

23. Zhang, X. *et al.* A general method for transition metal single atoms anchored on honeycomb-like nitrogen-doped carbon nanosheets. *Adv. Mater.* **32,** 1906905 (2020).

24. Cheng, Q. *et al.* Single cobalt atom and N codoped carbon nanofibers as highly durable electrocatalyst for oxygen reduction reaction. *ACS Catal.* **7,** 6864–6871 (2017).

25. Guan, B. Y., Le Yu & Lou, X. W. A dual-metal–organic-framework derived electrocatalyst for oxygen reduction. *Energy Environ. Sci.* **9,** 3092–3096 (2016).

26. Kim, S.-J. *et al.* Defect-free encapsulation of Fe0 in 2D fused organic networks as a durable oxygen reduction electrocatalyst. *J. Am. Chem. Soc.* **140,** 1737–1742 (2018).

27. Xiao, M. *et al.* Microporous framework induced synthesis of single-atom dispersed Fe-N-C acidic ORR catalyst and its in situ reduced Fe-N_4 active site identification revealed by X-ray absorption Spectroscopy. *ACS Catal.* **8,** 2824–2832 (2018).

28. Zhang, Z., Sun, J., Wang, F. & Dai, L. Efficient oxygen reduction reaction (ORR) catalysts based on single iron atoms dispersed on a hierarchically structured porous carbon framework. *Angew. Chem. Int. Ed.* **57,** 9038–9043 (2018).

29. Yang, L. *et al.* Unveiling the high-activity origin of single-atom iron catalysts for oxygen reduction reaction. *Proc. Natl. Acad. Sci. U. S. A.* **115,** 6626–6631 (2018).

30. Xiao, M. *et al.* Climbing the apex of the ORR volcano plot via binuclear site construction: electronic and geometric engineering. *J. Am. Chem. Soc.* **141,** 17763–17770 (2019).

31. Li, F. *et al.* Revealing isolated M-N_3C_1 active sites for efficient collaborative oxygen reduction catalysis. *Angew. Chem. Int. Ed.* **59,** 23678–23683 (2020).

32. Li, J.-C. *et al.* Stabilizing single-atom iron electrocatalysts for oxygen reduction via ceria confining and trapping. *ACS Catal.* **10,** 2452–2458 (2020).

33. Chen, J. *et al.* Dual single-atomic Ni-N_4 and Fe-N_4 sites constructing Janus hollow graphene for selective oxygen electrocatalysis. *Adv. Mater.* **32,** 2003134 (2020).

34. Shang, H. *et al.* Engineering isolated Mn-N_2C_2 atomic interface sites for efficient bifunctional oxygen reduction and evolution reaction. *Nano Lett.* **20,** 5443–5450 (2020).

35. Shang, H. *et al.* Engineering unsymmetrically coordinated Cu-S_1N_3 single atom sites with enhanced oxygen reduction activity. *Nat. Commun.* **11,** 3049 (2020).

36. Wang, C. *et al.* Engineering the coordination environment enables molybdenum single-atom catalyst for efficient oxygen reduction reaction. *J. Catal.* **389,** 150–156 (2020).

37. Wan, W. *et al.* Bifunctional single atom electrocatalysts: Coordination-performance correlations and reaction pathways. *ACS nano* **14,** 13279–13293 (2020).

38. Hou, Y. *et al.* Atomically dispersed nickel-nitrogen-sulfur species anchored on porous carbon nanosheets for efficient water oxidation. *Nat. Common* **10,** 1392 (2019).

39. Liu, Z. *et al.* Ultrafast construction of oxygen-containing scaffold over graphite for trapping Ni_{2+} into single atom catalysts. *ACS nano* **14,** 11662–11669 (2020).

40. Bai, L., Hsu, C.-S., Alexander, D. T. L., Chen, H. M. & Hu, X. A cobalt-iron double-atom catalyst for the oxygen evolution reaction. *J. Am. Chem. Soc.* **141,** 14190–14199 (2019).

41. Guan, J. *et al.* Water oxidation on a mononuclear manganese heterogeneous catalyst. *Nat. Catal.* **1,** 870–877 (2018).

42. Chen, P. *et al.* Atomically dispersed iron-nitrogen species as electrocatalysts for bifunctional oxygen evolution and reduction reactions. *Angew. Chem. Int. Ed.* **129,** 625–629 (2017).

43. Li, S., Cheng, C., Zhao, X., Schmidt, J. & Thomas, A. Active salt/silica-templated 2D mesoporous FeCo-Nx-carbon as bifunctional oxygen electrodes for zinc-air batteries. *Angew. Chem. Int. Ed.* **57,** 1856–1862 (2018).

44. Zheng, Y. *et al.* Molecule-level g-C_3N_4 coordinated transition metals as a new class of electrocatalysts for oxygen electrode reactions. *J. Am. Chem. Soc.* **139,** 3336–3339 (2017).

45. Zhang, Q., Duan, Z., Li, M. & Guan, J. Atomic cobalt catalysts for the oxygen evolution reaction. *Chem. Commun.* **56,** 794–797 (2020).

46. Ban, J. *et al.* Dual evolution in defect and morphology of single-atom dispersed carbon based oxygen electrocatalyst. *Adv. Funct. Mater.* **31,** 2010472 (2021).

47. Zhang, X. *et al.* Atomically dispersed hierarchically ordered porous Fe–N–C electrocatalyst for high performance electrocatalytic oxygen reduction in Zn-Air battery. *Nano Energy* **71,** 104547 (2020).

48. Pan, Y. *et al.* A bimetallic Zn/Fe polyphthalocyanine-derived single-atom Fe-N_4 catalytic site: a superior trifunctional catalyst for overall water splitting and Zn-Air batteries. *Angew. Chem. Int. Ed.* **57,** 8614–8618 (2018).

49. Zhang, J. *et al.* Single Fe atom on hierarchically porous S, N-codoped nanocarbon derived from porphyra enable boosted oxygen catalysis for rechargeable Zn-Air batteries. *Small* **15,** 1900307 (2019).

50. Han, X. *et al.* Oxygen reduction reaction: Mn-N_4 oxygen reduction electrocatalyst: operando investigation of active sites and high performance in Zinc–air battery. *Adv. Energy Mater.* **11,** 2170025 (2021).

51. Sun, H. *et al.* Boosting oxygen dissociation over bimetal sites to facilitate oxygen reduction activity of zinc-air battery. *Adv. Funct. Mater.* **31,** 2006533 (2021).

52. Gong, X. *et al.* Self-templated hierarchically porous carbon nanorods embedded with atomic Fe-N_4 active sites as efficient oxygen reduction Electrocatalysts in Zn-Air Batteries. *Adv. Funct. Mater.* **31,** 2008085 (2021).

53. Zhang, Z. *et al.* Single-atom catalysts: atomically dispersed cobalt trifunctional electrocatalysts with tailored coordination environment for flexible rechargeable Zn–air battery and self-driven water splitting. *Adv. Energy Mater.* **10,** 2070195 (2020).

54. Li, J.-C. *et al.* Highly dispersive cerium atoms on carbon nanowires as oxygen reduction reaction electrocatalysts for Zn-air batteries. *Nano letters* **21,** 4508–4515 (2021).

55. Wang, Y. *et al.* Hierarchical peony-like FeCo-NC with conductive network and highly active sites as efficient electrocatalyst for rechargeable Zn-air battery. *Nano Res.* **13,** 1090–1099 (2020).

56. Xiao, M. *et al.* Preferentially engineering FeN_4 edge sites onto graphitic nanosheets for highly active and durable oxygen electrocatalysis in rechargeable Zn-air batteries. *Adv. Mater.* **32,** 2004900 (2020).

57. Bai, Y. *et al.* Direct metalation of a phthalocyanine monolayer on Ag(111) with coadsorbed iron atoms. *J. Phys. Chem. C* **112,** 6087–6092 (2008).

58. Tang, C., Wang, B., Wang, H.-F. & Zhang, Q. Defect engineering toward atomic Co-N_x-C in hierarchical graphene for rechargeable flexible solid Zn-Air batteries. *Adv. Mater.* **29,** 1703185 (2017).

59. Artyushkova, K. *et al.* Density functional theory calculations of XPS binding energy shift for nitrogen-containing graphene-like structures. *Chem. Commun.* **49,** 2539–2541 (2013).

60. Bai, Y. *et al.* Adsorption of cobalt (II) octaethylporphyrin and 2H-octaethylporphyrin on Ag(111): new insight into the surface coordinative bond. *New J. Phys.* **11,** 125004 (2009).

61. Grosvenor, A. P., Kobe, B. A., Biesinger, M. C. & McIntyre, N. S. Investigation of multiplet splitting of Fe 2p XPS spectra and bonding in iron compounds. *Surf. Interface Anal.* **36,** 1564–1574 (2004).

62. Bediako, D. K. *et al.* Structure-activity correlations in a nickel-borate oxygen evolution catalyst. *J. Am. Chem. Soc.* **134,** 6801–6809 (2012).

63. Lee, S., Bai, L. & Hu, X. Deciphering iron-dependent activity in oxygen evolution catalyzed by nickel-iron layered double hydroxide. *Angew. Chem. Int. Ed.* **59,** 8072–8077 (2020).

64. Mavrič, A., Fanetti, M., Lin, Y., Valant, M. & Cui, C. Spectroelectrochemical tracking of nickel hydroxide reveals its irreversible redox states upon operation at high current density. *ACS Catal.* **10,** 9451–9457 (2020).

65. Wang, H. *et al.* Interaction between single gold atom and the graphene edge: a study via aberration-corrected transmission electron microscopy. *Nanoscale* **4,** 2920–2925 (2012).

66. Furnival, T. *et al.* Anomalous diffusion of single metal atoms on a graphene oxide support. *Chem. Phys. Lett.* **683,** 370–374 (2017).

67. Robertson, A. W. *et al.* Dynamics of single Fe atoms in graphene vacancies. *Nano Lett.* **13,** 1468–1475 (2013).

68. He, Z. *et al.* Atomic structure and dynamics of metal dopant pairs in graphene. *Nano Lett.* **14,** 3766–3772 (2014).
